# Spatiotemporal cellular map of the developing human reproductive tract

**DOI:** 10.1038/s41586-025-09875-2

**Published:** 2025-12-17

**Authors:** Valentina Lorenzi, Cecilia Icoresi-Mazzeo, Charlotte Cassie, Nadav Yayon, Elias R. Ruiz-Morales, Carmen Sancho-Serra, Ryan Colligan, Frederick C. K. Wong, Magda Marečková, Elizabeth Tuck, Kenny Roberts, Tong Li, Marc-Antoine Jacques, James Ashcroft, Xiaoling He, Berta Crespo, Batuhan Cakir, Simon Murray, Yong Gu, Alexander V. Predeus, Martin Prete, Iva Kelava, Roger Barker, Luz Garcia-Alonso, John C. Marioni, Roser Vento-Tormo

**Affiliations:** 1 Wellcome Sanger Institute, Cambridge, UK; 2 European Bioinformatics Institute–European Molecular Biology Laboratory, Cambridge, UK; 3 Wellcome–MRC Cambridge Stem Cell Institute, University of Cambridge, Cambridge, UK; 4 John van Geest Centre for Brain Repair, Cambridge University, Cambridge, UK; 5 HDBR, Great Ormond Street Institute of Child Health, University College London, London, UK

**Keywords:** Differentiation, Developmental biology, Reproductive biology

## Abstract

The human reproductive tract is essential for species perpetuation and overall health. Its development involves complex processes of sex specification, tissue patterning and morphogenesis, the disruption of which can cause lifelong issues, including infertility^1–5^. Here we present an extensive single-cell and spatial multi-omic atlas of the human reproductive tract during prenatal development to provide insights beyond those that are possible with smaller-scale, organ-focused studies. We describe potential regulators of sexual dimorphism in reproductive organs and pinpoint previously unknown genes involved in Müllerian duct emergence and regression and urethral canalization of the penis. By combining histological features with gene expression and chromatin accessibility data, we define transcription factors and signalling events potentially involved in the regionalization of the Müllerian and Wolffian ducts. We also refine how the HOX code is established in distinct reproductive organs and reveal that the expression of thoracic HOX genes is increased in the rostral mesenchyme of the fallopian tube and epididymis. Our findings further indicate that epithelial regionalization of the fallopian tube and epididymis, which probably contribute to sperm maturation and capacitation, is established during development. By contrast, later events are necessary for regionalization of the uterocervical canal epithelium. Finally, on the basis of single-cell data and fetal-derived organoids, we show that the fetal uterine epithelium is vulnerable to oestrogen-mimicking endocrine disruptors. By mapping sex-specific reproductive tract regionalization and differentiation at the cellular level, our study provides valuable insights into causes and potential treatments of developmental reproductive disorders.

## Abstract (web summary)

A detailed spatiotemporal roadmap of the human female and male reproductive tracts during key periods of sexual differentiation provides new cellular and molecular insights into how early axial gradients lead to specific cell lineages and tissue structures.

## Main

The development of the human reproductive tract is a complex morphogenetic process orchestrated by paracrine interactions^6^ and hormonal signalling^7^. The internal genitalia (with the exception of the gonads) originate from the Müllerian and Wolffian ducts (derived from intermediate mesoderm) and the urogenital sinus (derived from endoderm). In genetically female individuals (XX), the Müllerian ducts develop into the fallopian tubes, uterus, cervix and upper vagina, whereas the urogenital sinus forms the lower vagina^1,8^. In genetically male individuals (XY), the Wolffian ducts give rise to the epididymis, vas deferens and seminal vesicles, whereas the urogenital sinus becomes the prostate^2,9^. The genital tubercle, derived from lateral plate mesoderm, endoderm and surface ectoderm, gives rise to the external genitalia: the clitoris in female individuals and the penis in male individuals^3^.

Initially, embryonic reproductive tissue precursors (that is, Müllerian and Wolffian ducts, urogenital sinus and genital tubercle) comprise an undifferentiated epithelial inner layer and surrounding mesenchyme. As development progresses, the sexually dimorphic differentiation of the mesenchyme precedes and dictates the differentiation of the epithelium^6^. Müllerian and Wolffian duct differentiation is particularly complex, as precise spatial boundaries must be established between the resulting organs^10,11^.

Both Müllerian and Wolffian ducts are present in genetically female and male embryos until approximately 9–10 post-conceptional weeks (PCW). If the embryonic gonads differentiate into testes under the control of the Y chromosome-linked *SRY* gene^12^, Sertoli cells in the testes produce anti-Müllerian hormone (AMH), which causes the Müllerian ducts to regress^13^. Leydig cells in the testes secrete testosterone, which promotes the development of the Wolffian ducts into the male upper reproductive tract^9^, and which is further converted into dihydrotestosterone, leading to the development of the male lower reproductive tract^14^. In the absence of *SRY* and these hormones, as in XX embryos, the Wolffian ducts regress and the Müllerian ducts, urogenital sinus and genital tubercle develop into the female reproductive tract.

Genetic and environmental disruptions to reproductive tract development can lead to congenital anomalies, infertility and cancer^4,5^. For example, approximately 7% of women have congenital uterine anomalies, a figure that increases to around 17% among those who experience recurrent miscarriages^4^. However, the cellular and molecular mechanisms that mediate human reproductive tract development remain poorly studied and have primarily been inferred from rodent and chicken loss-of-function studies or human histological observations^15–17^. Recently, we and others have used single-cell transcriptomics to study the developing human reproductive tract, primarily focusing the gonads^18,19^ and, to a limited extent, the upper portions of the Müllerian and Wolffian ducts^20^. However, we lack a holistic study of development of the entire reproductive tract in both sexes. Notably, how the ducts are specified and patterned along their rostrocaudal axis in humans is largely unknown.

Here we generate a highly resolved, spatiotemporal, multi-omic map of the entire human reproductive tract (excluding the gonads) during development, profiling more than half a million cells spanning the first and second trimesters. We detail the cellular and molecular features of the female and male reproductive tracts throughout the critical stages of sexual differentiation and reveal how sex-specific signals drive the dimorphic development of reproductive organs and the regression of sexually unmatched ducts. We also resolve the cascade of gene-expression changes from tissue-wide gradients into lineage-specific compartments in the Müllerian and Wolffian ducts. Specifically, we define the key transcription factors and cell–cell communication events that drive their differentiation into final organ derivatives. Finally, we harness our atlas to pinpoint cell types and developmental windows that are probably affected by endocrine-disrupting chemicals (EDCs) and clinically approved drugs, and we validate the effect of two EDCs using fetal-derived human uterine organoids.

## Single-cell resolved spatiotemporal map

**Fig. 1:**
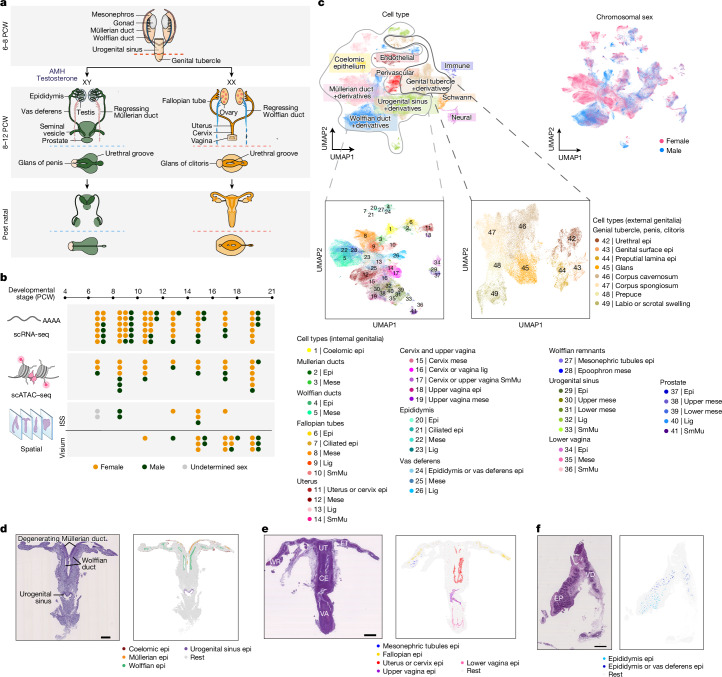
Single-cell resolved spatiotemporal atlas. **a**, Schematic of human reproductive development showing the main anatomical structures in XX and XY embryos and fetuses. **b**, Diagram summarizing the stage and sex composition of our donors along with the technologies used to characterize them. **c**, Top, batch-corrected uniform manifold approximation and projection (UMAP) embedding of the scRNA-seq dataset (*n* = 538,742 cells) coloured by major developmental cell lineages (left) and chromosomal sex (right). Bottom, batch-corrected UMAP embedding of reproductive-specific scRNA-seq cells from the internal genitalia (left; *n* = 379,663 cells) and external genitalia (right; *n* = 70,027 cells) coloured by cell type. **d**, Left, Image of a representative 10 PCW male fetus stained with haematoxylin and eosin (H&E) and profiled by ISS (*n* =  3 biologically independent samples). Scale bar, 1,000 μm. Right, inferred cell-type labels for selected cell types from the scRNA-seq dataset in the ISS slide. **e**, Left, H&E-stained image of a representative 17 PCW female fetus profiled by ISS (*n*  =  4 biologically independent samples). Scale bar, 2 mm. Right, inferred cell-type labels for selected cell types from the scRNA-seq dataset in the ISS slide. **f**, Left, H&E-stained image of a representative 16 PCW male fetus profiled by ISS (*n*  =  1 biologically independent sample) Scale bar, 500 μm. Right, inferred cell-type labels for selected cell types from the scRNA-seq dataset in the ISS slide. CE, cervix; EP, epididymis; Epi, epithelium; FT, fallopian tube; Lig, ligament; Mese, mesenchyme; SmMu, smooth muscle; UT, uterus; VA, vagina; VD, vas deferens; WR, Wolffian remnants. Illustrations in **a** and **b** created by A. García.

**Extended Data Fig. 1:**
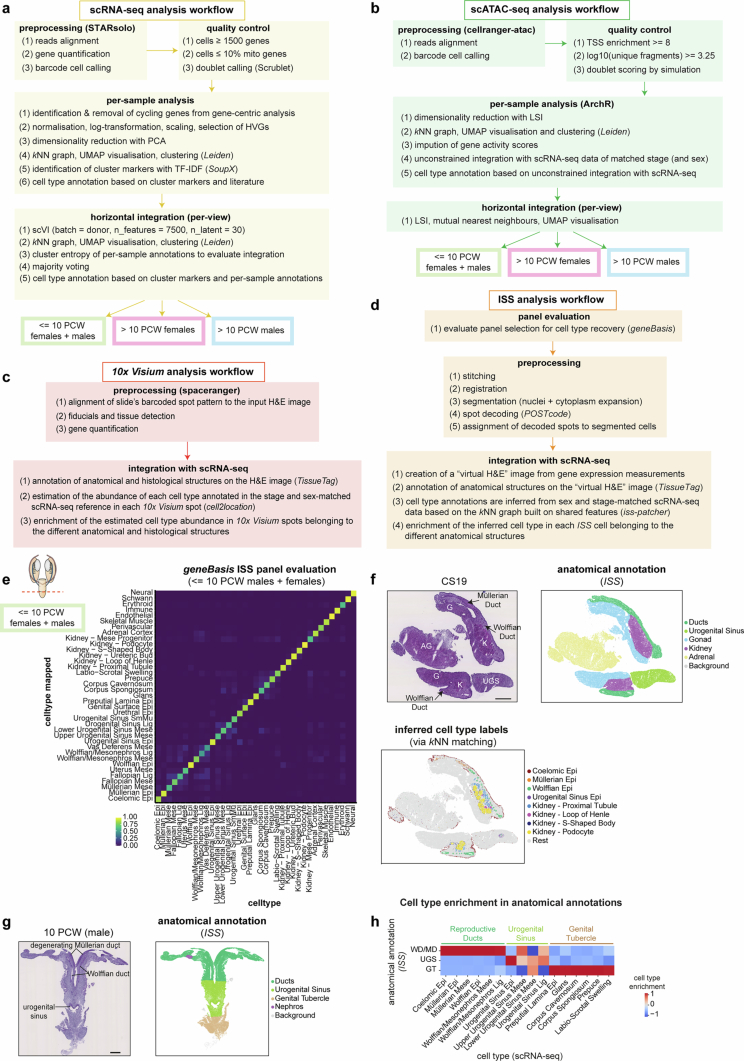
Computational workflows and spatial mapping of cell types in early (<= 10 PCW) female and male reproductive tract samples. **a**, Schematic representation of the computational workflow used to analyse scRNA-seq data. **b**, Schematic representation of the computational workflow used to analyse scATAC-seq data. **c**, Schematic representation of the computational workflow used to analyse *10x Visium* data and integrate it with scRNA-seq data. **d**, Schematic representation of the computational workflow used to analyse In Situ Sequencing (*ISS*) data and integrate it with scRNA-seq data. **e**, Confusion matrix evaluating the completeness of the gene panel selected for *ISS* at the cell type level for early (<= 10 PCW) male and female samples in our scRNA-seq data. **f**, (top left) Hematoxylin and eosin (H&E) stained image of a representative Carnegie stage (CS) 19 sample profiled with *ISS* alongside (top right) annotations of the anatomical structures and (bottom left) inferred cell type labels for selected cell types from the early (<= 10 PCW) scRNA-seq dataset in the *ISS* slide (n = 3 biologically independent samples). Scale bar: 500 μm. **g**, (left) H&E stained image of a representative 10 PCW male sample profiled with *ISS* alongside (right) annotations of the anatomical structures (n = 3 biologically independent samples). Scale bar: 1000 μm. **h**, Heatmap showing the z-score enrichment of each epithelial, mesenchymal, smooth muscle, and ligament cell type annotated in early male and female scRNA-seq samples <= 10 PCW (x-axis) in *ISS* cells corresponding to each anatomical annotation (y-axis). AG: adrenal gland; G: gonad; K: kidney; UGS: urogenital sinus.

We profiled 89 reproductive tract samples from fetuses aged 6–21 PCW, covering stages of sex specification and differentiation of the internal and external genitalia and the regression of the unmatched reproductive ducts. We used single-cell RNA sequencing (scRNA-seq; 538,742 cells), single-cell chromatin accessibility with sequencing (scATAC–seq; 226,668 cells), spatially resolved gene-expression profiling through in situ sequencing (ISS; 11 slides, 1,853,342 cells) and 10x Visium (36 slides) to generate the data (Fig. 1a,b and Supplementary Tables 1–3). By mapping the dissociated single-cell data onto stage-matched ISS and 10x Visium data, we enhanced the resolution and stringency of cell-type definitions, which led to the identification of 52 distinct reproductive-tract-specific cell types (Fig. 1c and Extended Data Fig. 1a–d). The integration of spatially resolved data was crucial for cell annotation, as unique markers for many of the identified cell types had not been previously described (Methods, Supplementary Note 1 (which also reports the references for markers previously reported in the literature and used to guide our annotations) and Supplementary Table 4).

In the early stages of development (until around 9 or 10 PCW), we identified coelomic epithelial (*UPK3B*^*+*^*LRRN4*^*+*^), Müllerian duct cells (*WNT7A*^*+*^*SOX17*^*+*^ epithelium and *AMHR2*^*+*^*CNTN1*^*+*^ mesenchyme) and Wolffian duct cells (*WNT9B*^*+*^*GATA3*^*+*^ epithelium and *PLAC1*^*+*^*HTR2B*^*+*^ mesenchyme) (Fig. 1c,d, Extended Data Fig. 1e–h and Supplementary Fig. 1a–c). We also detected cells from the urogenital sinus (*FOXA1*^*+*^*SHH*^*+*^ epithelium and *GAP43*^*+*^*TNC*^*+*^ upper and *FOXF1*^*+*^*FENDRR*^+^ lower mesenchyme) and genital tubercle (*UPK1A*^*+*^*PSCA*^*+*^ epithelium and *TBX4*^*+*^*TBX5*^*+*^ mesenchyme) (Fig. 1c,d, Extended Data Fig. 1e–h and Supplementary Fig. 1a–c). In samples from <8 PCW embryos, various cell types from adjacent kidney (*TMEM52*^+^ distal tubule, *SLC12A1*^+^ loop of Henle, *TM4SF4*^*+*^ S-shaped body, *GLYAT*^+^ proximal tubule and *NPHS1*^+^ podocyte) and adrenal glands (*SHISA3*^+^ adrenal cortex) were also present, as accurate tissue microdissection for this developmental stage is challenging (Extended Data Fig. 1f and Supplementary Fig. 1a–c).

**Extended Data Fig. 2:**
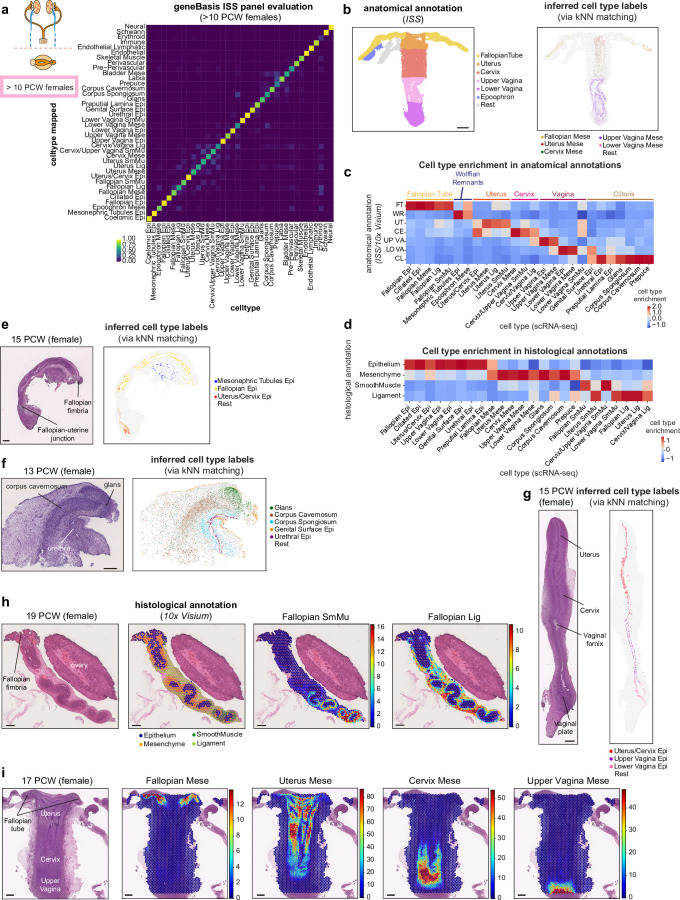
Spatial mapping of cell types in late (>10 PCW) female reproductive tract samples. **a**, Confusion matrix evaluating the completeness of the gene panel selected for *ISS* at the cell type level for late (>10 PCW) female samples in our scRNA-seq data. **b**, (left) Representative section of a 17 PCW female sample profiled with In Situ Sequencing (*ISS*) showing the annotations of the anatomical structures and (right) the inferred cell type labels for mesenchymal cell types from the scRNA-seq dataset in the *ISS* slide (n = 4 biologically independent samples). Scale bar: 2 mm. **c**, Heatmap showing the combined z-score enrichment of each epithelial, mesenchymal, smooth muscle and ligament cell type annotated in female scRNA-seq samples > 10 PCW (x-axis) in *10x Visium* spots and *ISS* cells corresponding to each anatomical annotation (y-axis). **d**, Heatmap showing the z-score enrichment of each epithelial, mesenchymal, smooth muscle and ligament cell type annotated in female scRNA-seq samples >10 PCW (x-axis) in *10x Visium* spots corresponding to each histological annotation (y-axis). **e**, (left) Hematoxylin and eosin (H&E) stained image of a representative 15 PCW female fallopian tube sample profiled with *ISS* alongside (right) the inferred cell type labels for epithelial cell types from the scRNA-seq dataset in the *ISS* slide (n = 3 biologically independent samples). Scale bar: 500 μm. **f**, (left) H&E stained image of a representative 13 PCW female external genitalia sample profiled with *ISS* alongside (right) the inferred cell type labels for selected cell types from the scRNA-seq dataset in the *ISS* slide (n = 1 biologically independent sample). Scale bar: 500 μm. **g**, (left) H&E stained image of a representative 15 PCW female uterovaginal canal sample profiled with *ISS* alongside (right) the inferred cell type labels for epithelial cell types from the scRNA-seq dataset in the *ISS* slide (n = 3 biologically independent samples). Scale bar: 500 μm. **h**, (left) H&E stained image of a representative 19 PCW female sample profiled with *10x Visium*. (middle) Annotations of the histological compartments and (right) spatial mapping of fallopian tube smooth muscle and ligament cells from the scRNA-seq dataset onto the corresponding *10x Visium* slide. Estimated cell type abundance (colour intensity) in each *10x Visium* spot is shown over the H&E image (n = 6 biologically independent samples). Scale bar: 500 μm. **i**, (left) H&E stained image of a representative 17 PCW female sample profiled with *10x Visium* alongside (right) spatial mapping of fallopian tube, uterine, cervical and upper vagina mesenchymal cells from the scRNA-seq dataset onto the corresponding *10x Visium* slide. Estimated cell type abundance (colour intensity) in each *10x Visium* spot is shown over the H&E image (n = 6 biologically independent samples). Scale bar: 500 μm.

**Extended Data Fig. 3:**
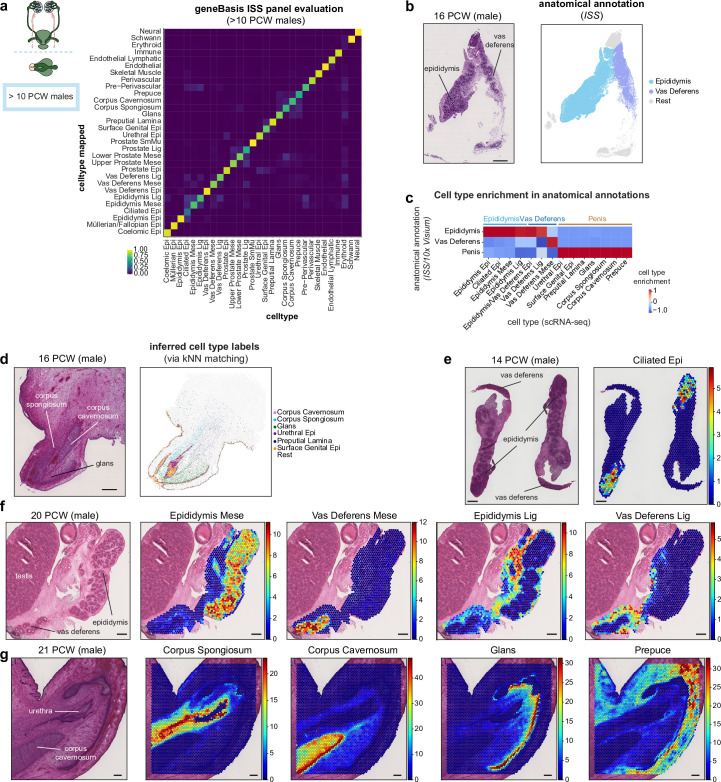
Spatial mapping of cell types in late (>10 PCW) male reproductive tract samples. **a**, Confusion matrix evaluating the completeness of the gene panel selected for *ISS* at the cell type level for late (>10 PCW) male samples in our scRNA-seq data. **b**, (left) Hematoxylin and eosin (H&E) stained image of a representative section of a 16 PCW male epididymis and vas deferens sample profiled with In Situ Sequencing (*ISS*) alongside (right) annotations of the anatomical structures (n = 1 biologically independent sample). Scale bar: 500 μm. **c**, Heatmap showing the combined z-score enrichment of each epithelial, mesenchymal, smooth muscle and ligament cell type annotated in male scRNA-seq samples > 10 PCW (x-axis) in *10x Visium* spots and *ISS* cells corresponding to each anatomical annotation (y-axis). **d**, (left) H&E stained image of a representative 16 PCW male external genitalia sample profiled with *ISS* alongside (right) the inferred cell type labels for selected cell types from the scRNA-seq dataset in the *ISS* slide (n = 1 biologically independent sample). Scale bar: 1000 μm. **e**, (left) H&E stained image of a representative 14 PCW male epididymis and vas deferens sample profiled with *10x Visium* alongside (right) spatial mapping of ciliated epithelial cells from the scRNA-seq dataset onto the corresponding *10x Visium* slide. Estimated cell type abundance (colour intensity) in each *10x Visium* spot is shown over the H&E image (n = 3 biologically independent samples). Scale bar: 500 μm. **f**, H&E stained image of a representative 20 PCW male epididymis and vas deferens sample profiled with *10x Visium* alongside (right) spatial mapping of mesenchymal and ligament cells from the scRNA-seq dataset onto the corresponding *10x Visium* slide. Estimated cell type abundance (colour intensity) in each *10x Visium* spot is shown over the H&E image (n = 3 biologically independent samples). Scale bar: 500 μm. **g**, H&E stained image of a representative 21 PCW male external genitalia sample profiled with *10x Visium* alongside (right) spatial mapping of selected cell types from the scRNA-seq dataset onto the corresponding *10x Visium* slide. Estimated cell type abundance (colour intensity) in each *10x Visium* spot is shown over the H&E image (n = 2 biologically independent samples). Scale bar: 500 μm.

As gestation progresses (9–21 PCW), female-specific cells emerged in the fallopian tubes (*PNOC*^*+*^*ERP27*^*+*^ non-ciliated and *DNAH12*^*+*^ ciliated epithelium and *ITGBL1*^*+*^*CD36*^*+*^ mesenchyme), uterocervix (*UCA1*^*+*^*DLX5*^*+*^ epithelium and *ITGA4*^*+*^*RORB*^*+*^ mesenchyme) and vagina (Müllerian-derived *DLX5*^*+*^*TP63*^*+*^ epithelium and *SRD5A2*^*+*^*GAP43*^*+*^ mesenchyme, urogenital sinus-derived *FOXA1*^*+*^*PRAC1*^*+*^ epithelium and *SRD5A2*^*+*^*FENDRR*^*+*^ mesenchyme) in XX fetuses (Fig. 1c,e, Extended Data Fig. 2a–i and Supplementary Fig. 2a–c). Notably, the uterus and cervix showed a highly similar cell-type composition, which indicated that further regionalization probably occurs after 21 PCW (Extended Data Fig. 2c). In the same time window (9–21 PCW), male-specific epididymis (*SPAG11B*^*+*^ non-ciliated and *DNAH12*^*+*^ ciliated epithelium and *PLAC1*^*+*^*HTR2B+* mesenchyme), vas deferens (*WNT9B*^*+*^*MUC6*^*+*^ epithelium and *RAI2*^*+*^*CHD7*^*+*^ mesenchyme) and prostate (*FOXA1*^*+*^ epithelium^21^ and *SRD5A2*^*+*^*GAP43*^*+*^ upper and *SRD5A2*^*+*^*FENDRR*^*+*^ lower mesenchyme) cells were detected in XY fetuses (Fig. 1c,f, Extended Data Fig. 3a–g and Supplementary Fig. 3a–c). Surrounding each organ of the developing female and male internal genitalia, there was also a layer of smooth muscle (*MYH11*^*+*^) and ligament (*PTGER3*^*+*^) (Extended Data Figs. 2d,h and 3f and Supplementary Note 1).

Our atlas further captured the remnants of sexually unmatched reproductive ducts that persisted in both sexes. Wolffian-like mesenchymal (*PLAC1*^*+*^) and epithelial (*FXYD2*^*+*^) cells were apparent near the fallopian tubes (epoophoron) in female fetuses until 21 PCW, whereas fallopian-like epithelial cells (*PNOC*^*+*^*ERP27*^*+*^) were observed in some male fetuses between 10 and 14 PCW. This finding indicates that Müllerian duct differentiation can occur in male individuals before its regression orchestrated by AMH concludes (Fig. 1c,e, Extended Data Fig. 2c,e and Supplementary Figs. 2a–c and 3a,b).

Consistent with studies of mice^22,23^, we did not identify sex-specific cell types in the developing penis or clitoris. Across all developmental stages and in both sexes, we identified the urethral epithelium (*FOXA1*^*+*^*PSCA*^*+*^), erectile tissues (corpus cavernosum (*SOX9*^*+*^*PRR16*^*+*^) and corpus spongiosum (*FOXF1*^*+*^*SALL1*^*+*^)), glans (*SP9*^*+*^*DLX5*^*+*^), prepuce (*SIX1*^*+*^*SHOX2*^*+*^), preputial lamina epithelium (*KRT14*^*+*^*WNT3*+) and surface genital epidermis (*KRT14*^*+*^*KRTDAP*^*+*^) (Extended Data Figs. 1h, 2c,f and 3c,d,g and Supplementary Figs. 1a–c, 2a–c and 3a–c).

In summary, our spatiotemporal, single-cell resource represents a highly comprehensive and unbiased characterization of the reproductive epithelia and surrounding mesenchyme during human prenatal development. It covers the progression from undifferentiated precursors to differentiated sex-specific organs, and is accessible at www.reproductivecellatlas.org.

## Müllerian emergence and regression

**Fig. 2:**
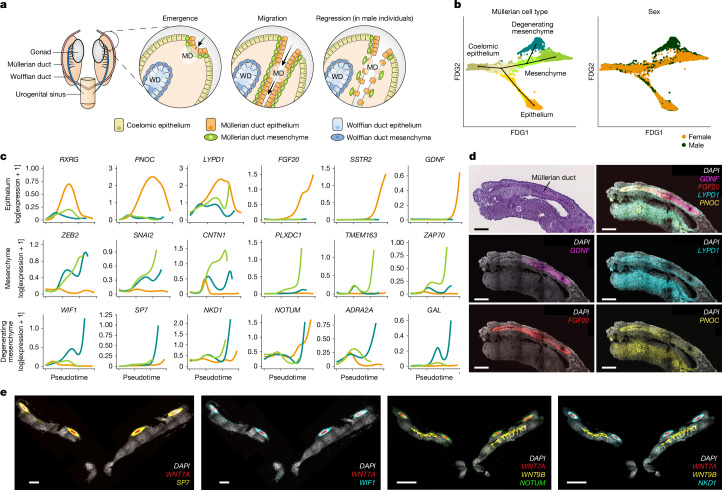
Müllerian ontology, migration and regression. **a**, Schematic of the major steps of Müllerian duct formation along with the cell types involved. **b**, Batch-corrected force directed graph (FDG) visualization of scRNA-seq data from 6–8 PCW fetuses, specifically the coelomic epithelium, Müllerian duct epithelium and Müllerian duct mesenchyme, coloured by cell type (left) and chromosomal sex (right). Reconstructed trajectories are overlaid on the embedding. **c**, Smoothed splines of key temporally variable genes involved in each differentiation trajectory of the Müllerian duct epithelium and mesenchyme (including male degenerating mesenchyme) from the coelomic epithelium. **d**, H&E-stained image and high-resolution, large-area images of a representative section of a Carnegie stage 19 embryo showing Müllerian epithelial emergence and migration. smFISH signals for *FGF20* (red, migrating Müllerian duct epithelium), *PNOC* (yellow, rostral Müllerian duct epithelium), *LYPD1* (cyan, rostral Müllerian duct epithelium) and *GDNF* (magenta, caudal migrating Müllerian duct epithelium) are highlighted (*n* = 3 biologically independent samples). Scale bars, 200 μm. G, gonad. **e**, High-resolution, large-area images of a representative section of a Carnegie stage 22 male fetus showing Müllerian mesenchymal regression (male-specific). smFISH signals for *WNT7A* (red, Müllerian duct epithelium), *SP7* (yellow, regressing Müllerian duct mesenchyme), *WIF1* (cyan, regressing Müllerian duct mesenchyme), *WNT9B* (yellow, Wolffian duct epithelium), *NOTUM* (green, regressing Müllerian duct mesenchyme) and *NKD1* (cyan, regressing Müllerian duct mesenchyme) are highlighted (*n* = 2 biologically independent samples). Scale bars, 200 μm (left two panels), 500 μm (right two panels). Illustrations in **a** created by A. García.

The Müllerian ducts initially consist of simple mesoepithelial tubes that are specified from the extra-gonadal coelomic epithelium^24^ around 6 PCW. According to studies in rodents, these cells migrate caudally in response to signalling from the Wolffian ducts^25^ (which emerge earlier, around 4 PCW, a developmental stage difficult to access and therefore not included in our study), and eventually fuse at the urogenital sinus (Fig. 2a).

We reconstructed cellular trajectories in the cell types that have key roles in the emergence, migration and initial regression of the Müllerian duct (between 6 and 8 PCW): the anterior mesonephric coelomic epithelium and the undifferentiated Müllerian epithelium and mesenchyme (Fig. 2a). We recovered two trajectories from the progenitor coelomic epithelium population to the Müllerian epithelium and mesenchyme. Notably, we also identified a male-specific degenerating mesenchymal lineage branching off the Müllerian mesenchyme (Fig. 2b).

**Extended Data Fig. 4:**
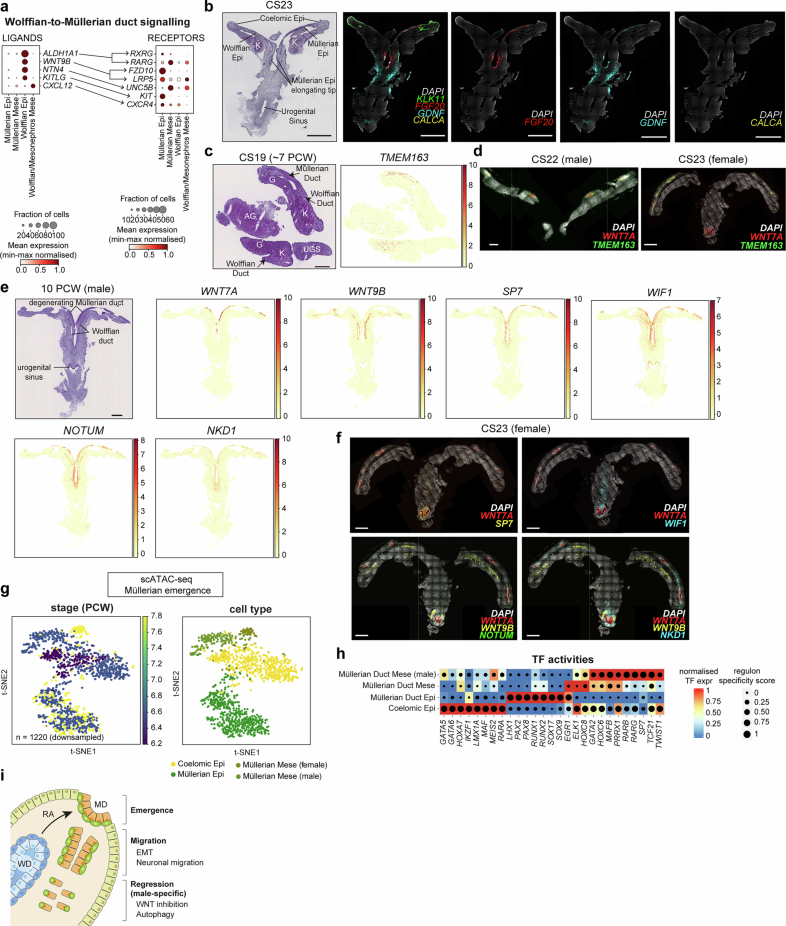
Ontology, migration and regression of the Müllerian duct. **a**, Dotplot showing the log-transformed, min-max normalised expression of Wolffian ligands and Müllerian receptors (y-axis) in each cell type (x-axis). Interacting ligands and receptors are connected with an arrow. **b**, Haematoxylin and eosin (H&E) stained image and high-resolution, large-area imaging of a representative section of a Carnegie Stage (CS) 23 sample with smFISH signal for *KLK11* (green, coelomic epithelium), *FGF20* (red, migrating Müllerian duct epithelium), *GDNF* (cyan, caudal migrating Müllerian duct epithelium), *CALCA* (yellow, caudal migrating Müllerian duct epithelium) (n = 3 biologically independent samples). Scale bar: 1000 μm. **c**, H&E image and *ISS* data of a representative section of a CS19 sample showing the measured expression of *TMEM163* in the Müllerian duct mesenchyme (n = 3 biologically independent samples). Scale: 500 μm. **d**, High-resolution, large-area imaging of a representative section of a (left) CS22 male sample and (right) CS23 female sample with smFISH signal for *WNT7A* (red, Müllerian duct epithelium) and *TMEM163* (green, migrating Müllerian duct mesenchyme) (n = 4 biologically independent samples, 2 males and 2 females). Scale bar: 200 μm (left panel), 500 μm (right panel). **e**, H&E image and *ISS* data of a representative section of a 10 PCW male sample showing the measured expression of *WNT7A*, *WNT9B*, *SP7*, *WIF1*, *NOTUM*, and *NKD1* (n = 1 biologically independent sample). Scale bar: 1000 μm. **f**, High-resolution, large-area imaging of a representative section of a CS23 female sample with smFISH signal for *WNT7A* (red, Müllerian duct epithelium), *SP7* (yellow, regressing Müllerian duct mesenchyme), *WIF1* (cyan, regressing Müllerian duct mesenchyme), *WNT9B* (yellow, Wolffian duct epithelium), *NOTUM* (green, regressing Müllerian duct mesenchyme), *NKD1* (cyan, regressing Müllerian duct mesenchyme) (n = 2 biologically independent samples). Scale bar: 500 μm. **g**, Batch corrected t-distributed Stochastic Neighbour Embedding (t-SNE) embedding of downsampled 6-8 PCW scATAC-seq cells (n = 1220) from the coelomic epithelium, Müllerian duct epithelium and Müllerian duct mesenchyme coloured by developmental stage (measured in PCW) and cell type. **h**, Heatmap showing the inferred regulon activity (from both scRNA-seq and scRNA-seq data 6-8 PCW samples) of the TFs (x-axis) involved in the formation of the Müllerian duct in each relevant cell type (y-axis). The colour scale is proportional to the expression of the TF while the size of the dot reflects the importance of the TF regulon in each cell type. **i**, Schematic illustration summarising the putative mechanisms underpinning the formation of the human Müllerian duct as found in our analysis. AG: adrenal gland; G: gonad, K: kidney; UGS: urogenital sinus. Illustrations in **i** created by A. García.

In the Müllerian epithelial lineage, genes such as *RXRG*,* PNOC* and *LYPD1* are transiently upregulated at the onset of mesothelial to epithelial cell differentiation (Fig. 2c and Supplementary Table 5). *ALDH1A1* expressed by the Wolffian epithelium is the probable source of retinoic acid signalling through the *RXRG–RARG* axis (Extended Data Fig. 4a and Supplementary Table 6). As the trajectory progresses, migratory genes such as *FGF20*, *SSTR2*, *GDNF*, *LGI1* and *CALCA*, which are known for their roles in neuronal migration and axonal outgrowth^26,27^, became upregulated (Fig. 2c and Supplementary Table 5). We validated the expression of *PNOC*,* LYPD1*,* FGF20*,* GDNF* and *CALCA* by multiplexed single-molecule fluorescence in situ hybridization (smFISH) (Fig. 2d and Extended Data Fig. 4b). smFISH imaging further revealed a coordinated patterning of these genes along the Müllerian duct epithelium. In detail, *FGF20* marked the length of the migrating ductal epithelium, whereas the expression of *PNOC* and* LYPD1* and of *GDNF* and* CALCA* were restricted to the rostral and caudal portions of the duct, respectively (Fig. 2d and Extended Data Fig. 4b).

The Müllerian mesenchymal lineage in turn was initially characterized by the upregulation of epithelial-to-mesenchymal transition markers such as *CNTN1*, *ZEB2* and *SNAI2*^28^ (Fig. 2c and Supplementary Table 5). The migratory genes *PLXDC1*, *ZAP70* and *TMEM163* were also upregulated, and *TMEM163* was confirmed by ISS and smFISH analyses (in both male and female embryos) (Fig. 2c and Extended Data Fig. 4c,d). By contrast, the male-specific degenerating branch showed increased expression of the autophagy modulators *ADRA2A*, *LAMP5* and* GAL*^29,30^, the WNT signalling inhibitors *NOTUM* and *NKD1* and two previously reported markers from the mouse literature, *SP7*^31^ and *WIF1* (also a WNT inhibitor)^32^ (Fig. 2c and Supplementary Table 5). The cell-type specificity of *NOTUM*, *NKD1*, *SP7* and *WIF1* was validated by ISS and smFISH, which revealed that these genes are not expressed in female fetuses (Fig. 2c,e and Extended Data Fig. 4e,f). The relevance of *SP7* in the degenerating male Müllerian mesenchyme was further supported by computationally inferring transcription factor activities from scATAC–seq and scRNA-seq data (Extended Data Fig. 4g,h).

Overall, our multimodal approach shows that human Müllerian duct formation probably involves the coordinated expression of migration genes in both mesenchyme and epithelium, alongside male-specific upregulation of WNT inhibitors and autophagy markers in the mesenchyme during Müllerian degeneration (Extended Data Fig. 4i).

## Müllerian and Wolffian patterning

We then investigated the differentiation and patterning of the Müllerian and Wolffian ducts into their final organ derivatives^8^. In rodents, once the migration process is complete, the Müllerian ducts in female animals are regionalized along the rostrocaudal axis, with cells in distinct segments acquiring specific identities to form the fallopian tubes, uterus, cervix and the upper part of the vagina^24^. Similarly, the Wolffian ducts regionalize along the rostrocaudal axis to give rise to the epididymis, vas deferens and seminal vesicle^9^.

**Fig. 3:**
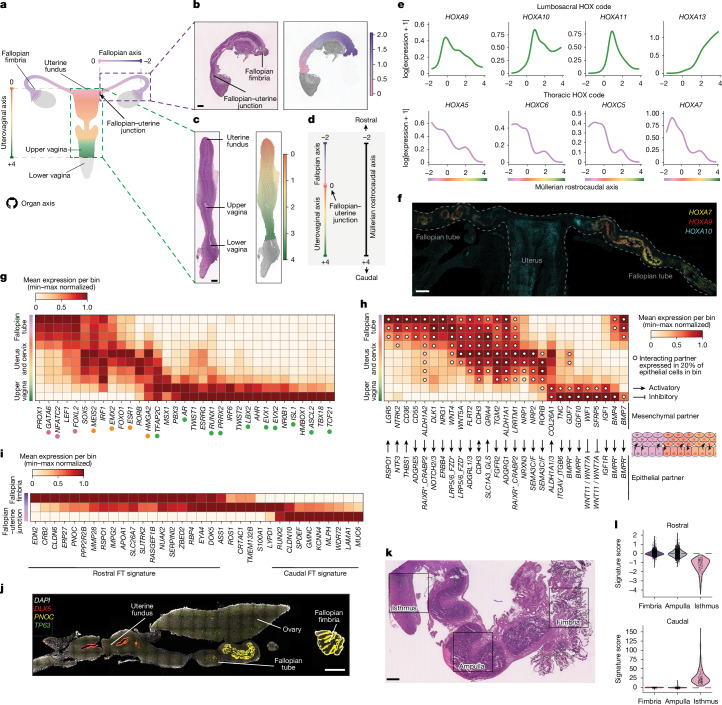
Müllerian and Wolffian patterning. **a**, Schematic of the fallopian and uterovaginal axes in >10 PCW female embryos. **b**, Left, H&E-stained image of a representative 15 PCW fallopian tube profiled with 10x Visium. Right, fallopian axis values overlaid per spot (*n* = 3 biologically independent samples). Scale bar, 500 μm. **c**, Left, stitched H&E-stained images of two consecutive sections from a representative 15 PCW uterovaginal canal profiled with 10x Visium. Right, uterovaginal axis values overlaid per spot (*n* = 2 biologically independent samples). Scale bar, 500 μm. **d**, Schematic of the Müllerian rostrocaudal axis. **e**, Smoothed splines of HOX transcription factors along the imputed Müllerian rostrocaudal axis in mesenchymal cells (scRNA-seq). **f**, High-resolution, large-area image of a representative 17 PCW female fetus with smFISH signals for *HOXA7* (yellow), *HOXA9* (red) and *HOXA10* (cyan) (*n* = 2 biologically independent samples). Scale bar, 500 μm. **g**, Heatmap showing minimum and maximum (min–max) normalized expression of prioritized spatially variable mesenchymal transcription factors (beyond the HOX code) in scRNA-seq data (*x* axis) along the binned imputed Müllerian rostrocaudal axis (*y* axis). Dots mark transcription factors identified by scATAC–seq and RNA-seq analyses (Extended Data Fig. 6b). **h**, Heatmap showing min–max normalized expression of prioritized spatially variable mesenchymal ligands and receptors in scRNA-seq data (*x* axis) along the binned imputed Müllerian rostrocaudal axis (*y* axis). Arrows show interacting epithelial partners. In epithelial partners, _ marks multimeric or cofactor-dependent receptors, / marks cases where both protein family members interact with the mesenchymal partner, and * marks cases where more than two interact. Dots mark expression in ≥20% of epithelial cells per bin. **i**, Heatmap showing min–max normalized expression of selected spatially variable genes in 10x Visium data in non-ciliated epithelium (*x* axis) along the binned fallopian tube (FT) axis (*y* axis). **j**, High-resolution, large-area images of a representative 21 PCW female fetus with smFISH signals for *DLX5* (red), *PNOC* (yellow) and *TP63* (green) (*n* = 2 biologically independent samples). Scale bar, 1,000 μm. **k**, H&E-stained image of a fallopian tube sample from a 37-year-old individual, highlighting three regions profiled by 10x Visium (*n* = 1 biologically independent sample). Scale bar, 2 mm. **l**, Violin plot of rostral (top) and caudal (bottom) signature scores from **i** in the epithelial 10x Visium spots across regions from **k**. Significance, Jonckheere’s trend test (*P* = 5 × 10^4^). Illustrations in **a** created by A. García.

**Extended Data Fig. 5:**
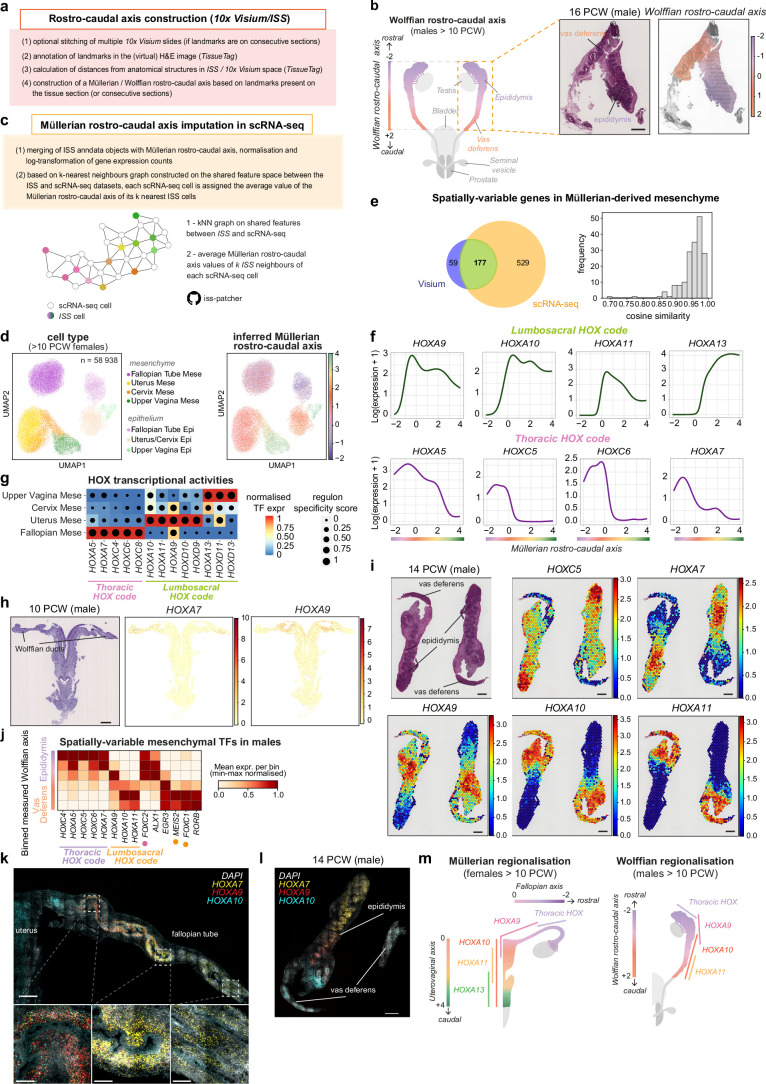
Mesenchymal patterning of the Müllerian and Wolffian ducts. **a**, Schematic representation of the computational workflow used to construct the *Müllerian* and *Wolffian rostro-caudal axes* from *10x Visium* and In Situ Sequencing (*ISS*) samples > 10 PCW. **b**, (left) Schematic illustration of the *Wolffian rostro-caudal axis*, which spans the developing epididymis and vas deferens and is computed from the haematoxylin and eosin (H&E) images of *10x Visium* male samples > 10 PCW. (middle) H&E image of a representative 16 PCW male sample profiled with *10x Visium* with annotated epididymis and vas deferens and with (right) *Wolffian rostro-caudal axis* values overlaid in each *10x Visium* (n = 3 biologically independent samples). Scale bar: 500 μm. **c**, Schematic representation of the computational workflow used to impute the *Müllerian rostro-caudal axis* in scRNA-seq data from *ISS* female samples > 10 PCW based on a shared k-nearest neighbours embedding (Supplementary Note 3). **d**, Batch-corrected Uniform Manifold Approximation and Projection (UMAP) embedding of epithelial and mesenchymal cells (n = 58,938) in female scRNA-seq samples > 10 PCW coloured by (left) cell type and (right) imputed *Müllerian rostro-caudal axis* value. **e**, (left) Venn diagram showing the intersection of genes that are identified as spatially-variable along the measured (in *10x Visium*) and imputed (in scRNA-seq) *Müllerian rostro-caudal axes* for mesenchymal spots. (right) Distribution of cosine similarities between the smoothing splines of the genes that are identified by both data modalities for mesenchymal spots/cells. **f**, Smoothed splines of *HOX* TFs along the measured *Müllerian rostro-caudal axis* in mesenchymal spots (*10x Visium*). **g**, Inferred regulon activity (from scRNA-seq/scATAC-seq data) of the *HOX* TFs (x-axis) in the differentiating Müllerian duct mesenchyme (y-axis). Colour reflects TF expression; dot size reflects regulon importance per cell type. **h**, (left) H&E image of a representative 10 PCW male sample profiled with *ISS* alongside (right) the measured expression of *HOXA7* and *HOXA9* in the Wolffian mesenchyme (n = 3 biologically independent samples). Scale bar: 1000 μm. **i**, H&E stained image and *10x Visium* data of a representative section of a 14 PCW male sample showing the measured expression of *HOXC5*, *HOXA7*, *HOXA9*, *HOXA10*, *HOXA11* (n = 3 biologically independent samples). Scale bar: 500 μm. **j**, Heatmap showing the min-max normalised expression measured in *10x Visium* data of prioritised spatially variable mesenchymal TFs (x-axis) along the binned measured *Wolffian rostro-caudal axis* (y-axis). TFs marked with a dot are also identified by our scATAC/RNA-seq analysis (as reported in Extended Data Fig. 6d). **k**, High-resolution, large-area imaging of a representative section of a 17 PCW female sample with smFISH signal for *HOXA7* (yellow, rostral fallopian tube mesenchyme), *HOXA9* (red, caudal fallopian tube mesenchyme), *HOXA10* (cyan, uterocervical mesenchyme) (n = 2 biologically independent samples, also shown in Fig. 3f). Dashed-lined boxes highlight fallopian tube regions magnified at the bottom of the panel. Scale bar: 500 μm (top panel) and 100um for figure zoom-ins. **l**, High-resolution, large-area imaging of a representative section of a 14 PCW male sample with smFISH signal for *HOXA7* (yellow, rostral epididymis), *HOXA9* (red, caudal epididymis), *HOXA10* (cyan, vas deferens) (n = 2 biologically independent samples). Scale bar: 500 μm. **m**, Schematic illustration summarising the mesenchymal patterning by *HOX* code genes along the differentiating Müllerian and Wolffian ducts found by our analysis. Illustrations in **b** and **m** created by A. García.

To explore how regional gene expression in Müllerian-derived and Wolffian-derived cells controls organ formation along the developing human reproductive tract, we used our spatially resolved transcriptomic data to generate a computational representation of the rostrocaudal axis^33^ in female fetuses (from the fallopian fimbriae to the end of the upper vagina) and male fetuses (from the efferent ductules to the initial segment of the vas deferens) (Fig. 3a–d, Extended Data Fig. 5a,b and Supplementary Notes 2 and 3). For female fetuses, for which we have several ISS samples available, we also projected the spatial rostrocaudal axis values from ISS onto our scRNA-seq data, assigning pseudospace coordinates to dissociated cells (Extended Data Fig. 5c,d and Supplementary Note 2). Finally, we modelled gene-expression changes along each axis using ISS-imputed scRNA-seq data (only for Müllerian ducts) and 10x Visium data (for both Müllerian and Wolffian ducts; Extended Data Fig. 5e, Supplementary Notes 2 and 3 and Supplementary Table 7).

We first examined the expression of the four lumbosacral HOX genes (*HOXA9*,* HOXA10*,* HOXA11* and* HOXA13*) that orchestrate the rostrocaudal regionalization of the Müllerian and Wolffian mesenchyme in rodents^11^. In female fetuses, *HOXA10* and* HOXA11* were upregulated in the uterocervical mesenchyme, whereas *HOXA13* was upregulated in the cervicovaginal mesenchyme, a result that aligns with previous studies^11^ (Fig. 3e and Extended Data Fig. 5f). However, although literature suggests that *Hoxa9* is upregulated throughout the fallopian tubes in mice^11^, human *HOXA9* exhibited increased expression in the caudal fallopian tube and uterocervical mesenchyme but was absent in the rostral fallopian tube mesenchyme (Fig. 3e and Extended Data Fig. 5f). This discrepancy prompted us to investigate other HOX genes that may be involved in patterning the rostral region of fallopian tubes in humans.

Thoracic HOX code members (including *HOXA5*, *HOXC5*, *HOXC6* and *HOXA7*) showed increased expression in the rostral fallopian tube mesenchyme, with a gradual decrease along the caudal axis (Fig. 3e and Extended Data Fig. 5f). Integrative analysis of scRNA-seq and scATAC–seq data further confirmed the activity of thoracic HOX regulons in the fallopian tube mesenchyme, whereas the lumbosacral HOX regulons were active in the uterovaginal mesenchyme (Extended Data Fig. 5g). Moreover, the Wolffian mesenchyme, where the upper Müllerian ducts (corresponding to the region that gives rise to fallopian tubes) are embedded, seemed to be patterned by *HOXA7* rostrally and *HOXA9* caudally from the earliest stages of development (Extended Data Fig. 5h). Consistent with the finding that the Wolffian mesenchyme is already patterned early in development, in male embryos, the thoracic code later (around 10–21 PCW) marks the upper half of the epididymis, whereas *HOXA9* is restricted to the lower half (Extended Data Fig. 5i,j). smFISH corroborated our single-cell and spatial transcriptomic findings. That is, *HOXA7* is enriched in the rostral fallopian tube and epididymis, whereas *HOXA9* is predominantly expressed in their caudal regions (Fig. 3f and Extended Data Fig. 5k,l).

**Extended Data Fig. 6:**
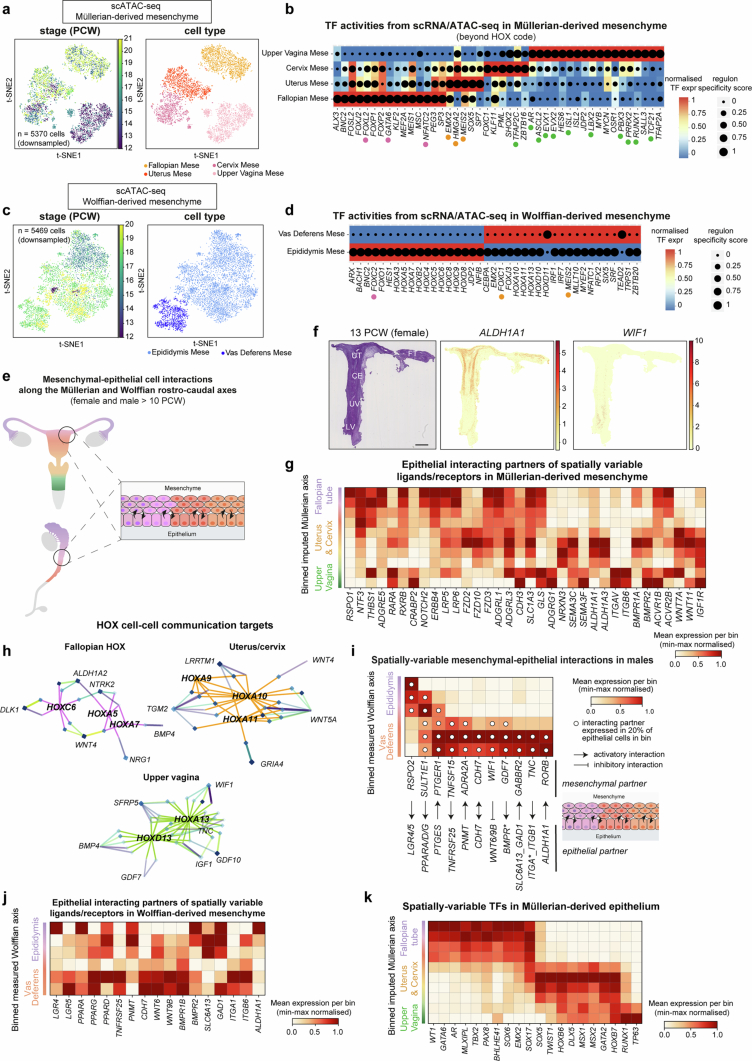
Mesenchymal patterning and mesenchymal-epithelial interaction along the differentiating Müllerian and Wolffian ducts. **a**, Batch corrected t-distributed Stochastic Neighbour Embedding (t-SNE) of downsampled Müllerian-derived mesenchymal cells from female samples > 10 PCW profiled with scATAC-seq (n = 5370 cells after downsampling) coloured by stage (measured in PCW) and cell type. **b**, Heatmap showing the inferred regulon activity (from both scRNA-seq and scRNA-seq data > 10 PCW female samples) of the TFs (x-axis) involved in the establishment of cellular diversity in the Müllerian duct-derived mesenchymal cells (y-axis). The colour scale is proportional to the expression of the TF while the size of the dot reflects the importance of the TF regulon in each cell type. **c**, Batch corrected t-SNE of downsampled Wolffian-derived mesenchymal cells from male samples > 10 PCW profiled with scATAC-seq (n = 5469 cells after downsampling) coloured by stage (measured in PCW) and cell type. **d**, Heatmap showing the inferred regulon activity (from both scRNA-seq and scRNA-seq data > 10 PCW male samples) of the TFs (x-axis) involved in the establishment of cellular diversity in the Wolffian duct-derived mesenchymal cells (y-axis). The colour scale is proportional to the expression of the TF while the size of the dot reflects the importance of the TF regulon in each cell type. **e**, Schematic illustration showing the bidirectional cell-cell communication between the mesenchyme and epithelium of the differentiating Müllerian and Wolffian ducts along their rostro-caudal axes in female and male samples > 10 PCW. **f**, (left) Hematoxylin and eosin (H&E) image of a representative 13 PCW female sample profiled with In Situ Sequencing (*ISS*) alongside (right) measured expression of *ALDH1A1* and *WIF1* (n = 3 biologically independent samples). Scale bar: 100 μm. **g**, Heatmap showing the min-max normalised expression measured in scRNA-seq data of epithelial partners of the spatially variable mesenchymal ligand/receptors (x-axis) along the binned imputed *Müllerian rostro-caudal axis* (y-axis). **h**, Network representation of the gene regulatory network for HOX code TFs only including targets that were identified as spatially-variable in the Müllerian-derived mesenchyme. **i**, Heatmap showing the min-max normalised expression measured in *10x Visium* data of prioritised spatially variable mesenchymal ligands or receptors (x-axis) along the binned measured *Wolffian rostro-caudal axis* (y-axis). Interacting partners for each spatially variable mesenchymal ligand/receptor are reported on the x-axis below the activatory or inhibitory arrows. A dot in a bin indicates that the interacting partner of the spatially variable mesenchymal ligand/receptor is expressed in at least 20% of epithelial cells in that bin. **j**, Heatmap showing the min-max normalised expression measured in *10x Visium* data of epithelial partners of the spatially variable mesenchymal ligand/receptors (x-axis) along the binned measured *Wolffian rostro-caudal axis* (y-axis). **k**, Heatmap showing the min-max normalised expression measured in scRNA-seq data of prioritised spatially variable epithelial TFs (x-axis) along the binned imputed *Müllerian rostro-caudal axis* (y-axis). CE: cervix; FT: fallopian tube; LV: lower vagina; UT: uterus; UV: upper vagina. Illustrations in **e** created by A. García.

We then explored transcription factors that might determine the regional specificity of the mesenchyme in the ducts beyond the HOX code. In the Müllerian mesenchyme, we observed a decreasing gradient of *GATA6*, *PROX1*, *NFATC2* and *FOXL2* expression and an increasing gradient of *PBX3*, *PRRX2*, *EVX1*, *EVX2*, *LBX2*, *AHR*, *AR* and *ISL1* expression along the rostrocaudal axis, some of which were also found to be active by means of scATAC–seq (Fig. 3g and Extended Data Fig. 6a,b). These transcription factors included homeobox genes (*GATA6*, *PBX3*, *PRRX2*, *EVX1*, *EVX2*, *LBX2* and *ISL1*) implicated in mesodermal rostrocaudal patterning of other organs in multiple species^34–36^. Moreover, the central portion of the Müllerian axis (corresponding to the uterus and cervix) was characterized by the upregulation of *EMX2*, *ESR1*, *FOXO1, MEIS2* and *RORB* (Fig. 3g and Extended Data Fig. 6a,b).

We observed a distinct pattern of transcription factors in the Wolffian-derived mesenchyme, with *FOXC2* and *ALX1* marking the rostral and caudal portions of the epididymis (the male analogue of the fallopian tubes), respectively (Extended Data Figs. 5j and 6c,d). The portion of the Wolffian axis corresponding to the upper vas deferens (the male analogue of the uterus) showed specific expression of *FOXC1* and upregulation of *MEIS2* and *RORB*, consistent with its female uterine counterpart (Extended Data Figs. 5j and 6c,d). Owing to damage to the vas deferens during dissections, we could not define shared and specific transcription factors in the lower part of the vas deferens and seminal vesicles.

Altogether, our work refined the HOX code that underlies mesenchymal regionalization in the differentiating human Müllerian and Wolffian ducts (Extended Data Fig. 5m) and identified previously unknown spatially variable transcription factors, some shared between the sexes and others sex-specific.

## Signals that guide ductal patterning

Heterotypic co-culturing of epithelial and mesenchymal cells of the reproductive tract has shown that mesenchymal cells in the ducts first acquire their regional identity and then instruct the adjacent epithelium to differentiate accordingly^6^. Hence, we next performed cell–cell communication analyses to identify specific interactions between the mesenchyme and epithelium along the Müllerian and Wolffian duct rostrocaudal axes (Extended Data Fig. 6e and Supplementary Notes 2 and 3).

We found increased activity of WNT and retinoic acid signalling (mediated by the mesenchymal-expressed ligands *WNT4* and *WNT5A* and *ALDH1A1*) in the fallopian tubes and uterus (Fig. 3h and Extended Data Fig. 6f–h). This activity was opposed by an increasing gradient of WNT inhibition (driven by the upregulation of *WIF1* and *SFRP5* in the mesenchyme) in the upper vagina, results that corroborated existing mouse literature^37^ (Fig. 3h and Extended Data Fig. 6f–h). Similarly, in the caudal portion of the Wolffian duct axis (corresponding to the vas deferens), there was an increase in WNT inhibition (driven by *WIF1* expressed by the mesenchyme) (Extended Data Fig. 6i,j).

**Extended Data Fig. 7:**
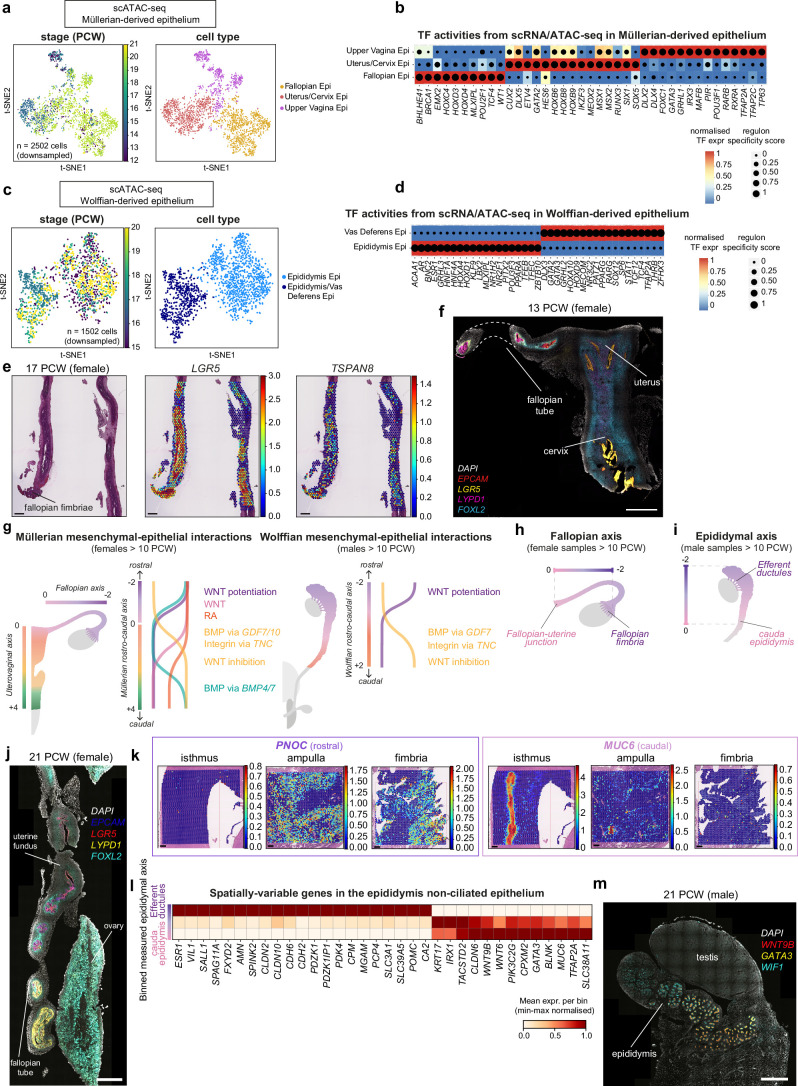
Epithelial regionalisation along the differentiating Müllerian and Wolffian ducts and within the fallopian tube and epididymis. **a**, Batch corrected t-SNE embedding of downsampled Müllerian-derived epithelial cells (n = 2502 cells after downsampling) from female samples > 10 PCW profiled with scATAC-seq coloured by stage (measured in PCW) and cell type. **b**, Heatmap showing the inferred regulon activity (from both scRNA-seq and scRNA-seq data > 10 PCW female samples) of the TFs (x-axis) involved in the establishment of cellular diversity in the Müllerian duct-derived epithelial cells (y-axis). The colour scale is proportional to the expression of the TF while the size of the dot reflects the importance of the TF regulon in each cell type. **c**, Batch corrected t-SNE embedding of Wolffian-derived epithelial cells (n = 1502 cells after downsampling) from male samples > 10 PCW profiled with scATAC-seq coloured by stage (measured in PCW) and cell type. **d**, Heatmap showing the inferred regulon activity (from both scRNA-seq and scRNA-seq data > 10 PCW male samples) of the TFs (x-axis) involved in the establishment of cellular diversity in the Wolffian duct-derived epithelial cells (y-axis). The colour scale is proportional to the expression of the TF while the size of the dot reflects the importance of the TF regulon in each cell type. **e**, (left) Hematoxylin and eosin (H&E) image of a representative 17 PCW fallopian tube sample profiled with *10x Visium* alongside (right) measured expression of *LGR5* and *TSPAN8* in each spot (n = 6 biologically independent samples). Scale bar: 500 μm. **f**, High-resolution, large-area imaging of a representative section of a 13 PCW female sample with smFISH signal for *EPCAM* (red, epithelium), *LGR5* (yellow, fallopian tube mesenchyme and uterocervical epithelium), *LYPD1* (magenta, middle fallopian tube epithelium), *FOXL2* (cyan, fallopian and uterocervical mesenchyme) (n = 3 biologically independent samples). Scale bar: 1000 μm. **g**, Schematic illustration summarising the mesenchymal-epithelial signalling present along the differentiating Müllerian and Wolffian ducts found in our analysis. **h**, Schematic illustration of the derivation of the *fallopian axis* in female samples > 10 PCW (this is part of the *Müllerian rostro-caudal axis* shown in Fig. 3a–d). **i**, Schematic illustration of the derivation of the *epididymal axis* in male samples > 10 PCW (this is part of the *Wolffian rostro-caudal axis* shown in Extended Data Fig. 5b). **j**, High-resolution, large-area imaging of a representative section of a 21 PCW female sample with smFISH signal for *EPCAM* (blue, epithelium), *LGR5* (red, fallopian tube mesenchyme and uterine epithelium), *LYPD1* (yellow, middle fallopian tube epithelium), *FOXL2* (cyan, fallopian tube and uterine mesenchyme) (n = 3 biologically independent samples). Scale bar: 1000 μm. **k**, Normalised, log-transformed expression of (left) *PNOC* and (right) *MUC6* in each *10x Visium* spot of capture areas in Fig. 3k. Scale bars: 500 μm. **l**, Heatmap showing the min-max normalised expression measured in *10x Visium* data of selected spatially variable non-ciliated epithelial genes (x-axis) along the binned *epididymal axis* (y-axis). **m**, High-resolution, large-area imaging of a representative section of a 21 PCW male sample with smFISH signal for *WNT9B* (red, caudal epididymal epithelium), *GATA3* (yellow, caudal epididymal epithelium), *WIF1* (cyan, epididymal mesenchyme) (n = 3 biologically independent samples). Scale bar: 1000 μm. Illustrations in **g**–**i** created by A. García.

In the upper vagina, we observed increased signalling through the *IGF1–IGF1R* axis, integrin pathways involving *TNC*, and BMP activity mediated by *GDF7*, *GDF10*, *BMP4* and *BMP7* through *BMPR*, with each respective ligand being expressed in the mesenchyme. BMP signalling could induce the upregulation of *RUNX1* and *TP63* in the adjacent epithelium, as previously reported in mice^38^ and in keeping with our analysis of spatially variable transcription factors in the differentiating Müllerian epithelium (Fig. 3h and Extended Data Figs. 6g,h,k and 7a,b). Although *GDF7* and *TNC* expression peaked in the upper vagina, they were already expressed in the uterocervical mesenchyme (Fig. 3h). This expression pattern was mirrored in male embryos, for which BMP signalling via *GDF7* and integrin signalling via *TNC* (both expressed by the mesenchyme) were upregulated in the initial segment of the vas deferens (Extended Data Figs. 6i,j and 7c,d).

Signalling between the mesenchyme and epithelium during ductal regionalization can be bidirectional, with signals from the epithelium also influencing the fate of the mesenchyme^6^. Consistent with this finding, we observed upregulation of the *LGR5* receptor in fallopian tube mesenchyme, which may respond to its cognate ligand *RSPO1* expressed in the adjacent epithelium (Fig. 3h). Moreover, co-expression of *LGR5* and *TSPAN8* in the fallopian mesenchyme suggests features reminiscent of a stem cell niche^39^ (Extended Data Fig. 7e,f).

In summary, by investigating mesenchymal–epithelial cell interactions along the Müllerian and Wolffian rostrocaudal axes, we identified shared and sex-specific cell communication events that are probably pivotal in determining epithelial identity during the regionalization of the reproductive ducts (Extended Data Fig. 7g).

## Fallopian and epididymal regionalization

In adulthood, the non-ciliated epithelia of the fallopian tubes and epididymis are functionally regionalized to support sperm capacitation and maturation, as reflected in marked gene-expression differences. However, it is unclear whether and when this regional differentiation occurs during fetal development^40^. To evaluate the in utero transcriptional gradients at the genome-wide level, we leveraged our Müllerian and Wolffian rostrocaudal axes analysis framework and examined intra-organ gene-expression changes in the developing fallopian tube and epididymis epithelia in fetuses between 10 and 21 PCW (Extended Data Fig. 7h,i, Supplementary Note 4 and Supplementary Table 8).

In the non-ciliated epithelial cells of the fetal fallopian tube, we identified genes (including *PNOC*, *APOA1*, *CLDN6*, *ERP27* and *ZBED2*) for which expression decreased rostrocaudally from the fimbria to the isthmus, and genes for which expression peaked in the middle of the fallopian tube (for example, *LYPD1*, *S100A1* and *CRTAC1*) (Fig. 3i,j and Extended Data Fig. 7j). Notably, *PNOC* and *LYPD1* expression was already restricted to the rostral portion of the epithelium during Müllerian duct emergence, which indicated that some degree of regionalization begins early in development (Fig. 2d). We also observed upregulation of genes such as *MUC6*, *WDR72* and *KCNN4* in the fallopian isthmus (Fig. 3i). Orthologues of these genes are involved in isthmus-specific epithelial secretions in other species^41,42^.

Some genes are known to change their expression^43^ along the rostrocaudal axis of the fallopian tubes in adults, but a comprehensive study is lacking. Thus, to determine whether the spatial gradient we identified in the fetus persists into adulthood, we generated 10x Visium spatial transcriptomic data from three regions of a human adult fallopian tube (fimbria, ampulla and isthmus) and scored epithelial spots in each region for the average expression of our fetal gene sets (Fig. 3k and Extended Data Fig. 7k). Both the rostral-biased and caudal-biased trends observed in fetal development were indeed maintained in adulthood (Fig. 3l).

In the non-ciliated epithelium of the fetal epididymis, we uncovered genes with a rostral bias, including *ESR1*, *SALL1*, *VIL1*, *SPAG11A*, *PDZK1* and *FXYD2*, which are known regulators of fluid reabsorption and sperm maturation^44^ in the adult epididymis (Extended Data Fig. 7l). Moreover, cell-adhesion genes such as the claudins (*CLDN2* and *CLDN10*) and cadherins (*CDH2* and *CDH6*) were enriched in the rostral portion of the epididymis, a result consistent with findings in adult tissues^45^. Moreover, several genes that exhibited increased expression towards the caudal epididymis—*GATA3*, *WNT9B*, *TFAP2A*, *CPXM2* and *BLNK*—have also been reported in adults and are associated with immune response regulation^45^ (Extended Data Fig. 7l,m).

Taken together, our findings indicate that the regional differentiation of the human fallopian tube and epididymis begins in utero and establishes transcriptional gradients that can persist into adulthood.

## Sexual dimorphism in the genital tubercle

**Extended Data Fig. 8:**
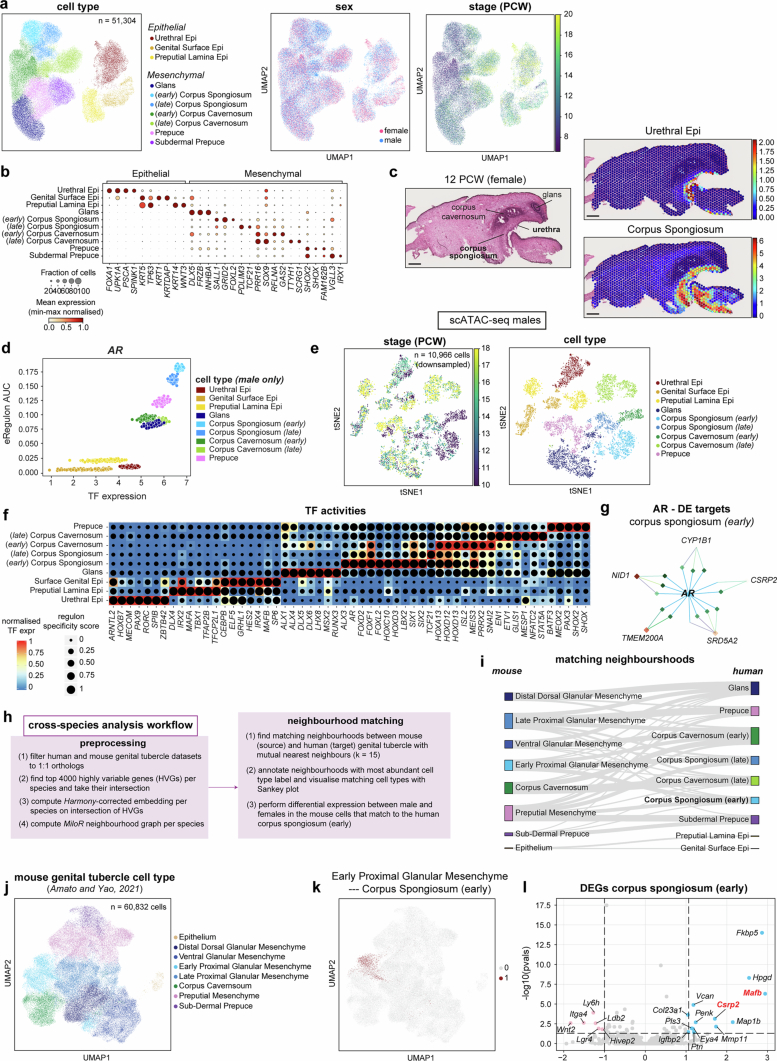
Sexual dimorphism in the external genitalia and comparison with mice. **a**, Batch-corrected Uniform Manifold Approximation and Projection (UMAP) embedding of the epithelial and mesenchymal cells in the external genitalia profiled with scRNA-seq (n = 51,304 cells) coloured by cell type, chromosomal sex, and developmental stage (measured in PCW). **b**, Dot plot showing the variance-scaled, log-transformed expression of genes (x-axis) characteristic of the annotated cell types (y-axis) detected in the external genitalia. Top-layer groups marker genes by lineage (epithelial or mesenchymal). **c**, Hematoxylin and eosin (H&E) stained image of a representative 12 PCW female sample profiled with *10x Visium* alongside spatial mapping of urethral epithelium and *corpus spongiosum* cells from the scRNA-seq dataset onto the corresponding *10x Visium* slide. Estimated cell type abundance (colour intensity) in each *10x Visium* spot is shown over the H&E image (n = 2 biologically independent samples). Scale bar: 500 μm. **d**, Scatter plot showing the area under the curve of the inferred activity of the chromatin region-based cistrome for *AR* (y-axis) as a function of its expression (x-axis) in each scATAC/RNA-seq metacell derived from the male external genitalia. Each metacell is coloured by cell type. **e**, Batch corrected t-distributed Stochastic Neighbour Embedding (t-SNE) embedding of downsampled scATAC-seq cells (n = 10,966 cells after downsampling) from the male external genitalia coloured by developmental stage (measured in PCW) and cell type. **f**, Heatmap showing the inferred regulon activity (from both scRNA-seq and scRNA-seq data of male samples) of the TFs (x-axis) involved in the establishment of cellular diversity in the external genitalia (y-axis). The colour scale is proportional to the expression of the TF while the size of the dot reflects the importance of the TF regulon in each cell type. **g**, Network representation of the gene regulatory network for *AR* only including targets that were identified as male-biased in the *early corpus spongiosum*. Nodes in green correspond to genomic regions which contain an AR binding motif, while nodes in red are the inferred target genes. **h**, Schematic representation of the computational workflow used to compare human and mouse external genitalia during the masculinisation programming window. **i**, Sankey plot representing the matching neighbourhoods between the mouse and human external genitalia labelled by the most abundant cell type in the neighbourhood. **j**, Batch corrected UMAP embedding of embryonic day (E) 14.5, E16.5, E18.5 (corresponding to the masculinisation programming window in mice) scRNA-seq cells (n = 60,832 cells) from the mouse external genitalia^23^ coloured by cell type. The cell type annotations are taken from the authors of the original study. **k**, Batch corrected UMAP embedding of embryonic day (E) 14.5, E16.5, E18.5 scRNA-seq cells from the mouse external genitalia highlighting the neighbourhoods that match the human *corpus spongiosum*. **l**, Volcano plot showing the log fold-change (x-axis) and adjusted p-value (y-axis) of the differential expression test (adjusted p-value = 0.05, |logFC| > 1) between male and female samples within the mouse *corpus spongiosum*. Source data

We next investigated how sexual dimorphism emerges in the external genitalia, where androgen action drives penile growth and canalization of the male urethra^3,16^. Although we did not identify sex-specific cell populations in the developing genital tubercle (Extended Data Fig. 8a), we observed stage-dependent differences in the mesenchymal erectile tissues that distinguished *RFLNA*^+^*GAS2*^+^ early corpus cavernosum and *TTYH1*^*+*^*SCRG1*^*+*^ late corpus cavernosum, as well as *GRIDL2*^+^*FOXL2*^*+*^ early corpus spongiosum and *PDLIM3*^+^*TCF21*^+^ late corpus spongiosum (Extended Data Fig. 8a,b). The masculinization programming window (MPW), which is estimated to occur between 8 and 14 PCW in humans, is the critical period during which disruptions in androgen signalling have the most significant phenotypic effects on newborn male individuals^46^. Studies in rodents suggest that during this window, the early corpus spongiosum, located adjacent to the invaginating urethral epithelium, has a crucial role in urethral canalization by moving medially and shaping the developing urethral canal in the penis^47^.

**Fig. 4:**
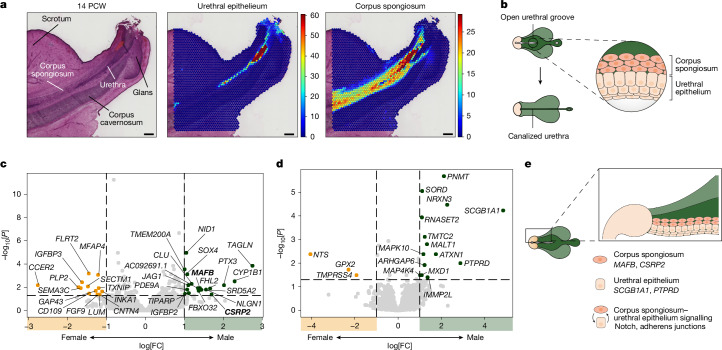
Sexual dimorphism in the genital tubercle. **a**, H&E-stained image of a representative 14 PCW male fetus profiled with 10x Visium alongside spatial mapping of urethral epithelium and corpus spongiosum cells from the scRNA-seq dataset onto the corresponding 10x Visium slide (*n* = 2 biologically independent samples). Estimated cell-type abundance (colour intensity) in each 10x Visium spot is shown over the H&E image. Scale bars, 500 μm. **b**, Schematic of the process of urethral canalization that occurs in male external genitalia during the MPW (around 8–14 PCW). **c**, Volcano plot showing the log fold change (FC) (*x* axis) and adjusted *P* value (*y* axis) of the differential expression of genes (adjusted *P* = 0.05, |log[FC]| > 1) between male and female fetuses in the human early corpus spongiosum. Genes in bold were also identified as being significantly upregulated in the male mouse early corpus spongiosium. **d**, Volcano plot showing the log fold change (*x* axis) and adjusted *P* value (*y* axis) of the differential expression of genes (adjusted *P* = 0.05, |log[FC]| > 1) between male and female fetuses in the human urethral epithelium. **e**, Schematic summarizing the putative drivers of urethral canalization identified through our analyses. Illustrations in **b** and **e** created by A. García. Source data

To investigate the molecular underpinnings of urethral canalization in humans, we first validated the identity of the urethral epithelium and surrounding corpus spongiosum in both the penis and clitoris through spatial mapping (Fig. 4a and Extended Data Fig. 8c). In the developing penis, the early corpus spongiosum showed the highest activity of androgen receptor compared with all other cell types (Extended Data Fig. 8d–f).

**Extended Data Fig. 9:**
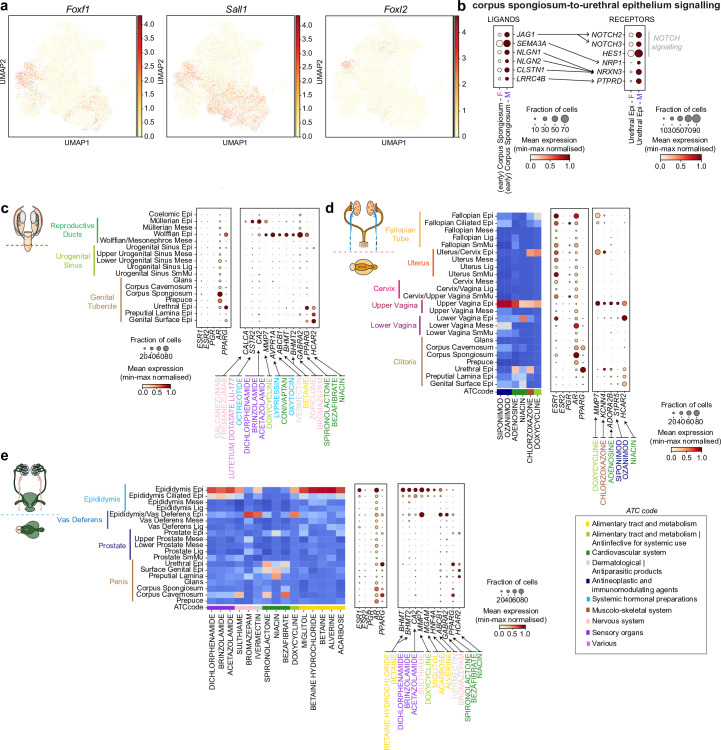
Sexual dimorphism in the external genitalia and potential disruptions to epithelial reproductive development. **a**, Batch corrected Uniform Manifold Approximation and Projection (UMAP) embedding of E14.5, E16.5, E18.5 scRNA-seq cells from the mouse external genitalia showing the expression of *early corpus spongiosum* markers *Foxf1*, *Sall1*, *Foxl2*. **b**, Dotplot showing the log-transformed, min-max normalised expression of *early corpus spongiosum* ligands and urethral epithelium receptors (y-axis) in each cell type (x-axis), separated by sex. Interacting ligands and receptors are connected by an arrow. **c**, Dot plots showing the log-transformed, variance-scaled expression of steroidogenic receptors (x-axis) and target genes (x-axis) of the clinically approved drugs in Fig. 5a in each reproductive-specific cell type (y-axis) identified in our early (<= 10 PCW) scRNA-seq dataset. **d**, (left) Heatmap showing the z-score enrichment of targets of clinically approved drugs (x-axis) found to be specifically impacting the epithelial compartment of late female (>10 PCW) reproductive tract organs among reproductive-specific cell types (y-axis) identified in our scRNA-seq dataset. (right) Dot plots showing the log-transformed, variance-scaled expression of the steroidogenic receptors (x-axis) and target genes (x-axis) of the clinically approved drugs in (right) in each reproductive-specific cell type (y-axis) identified in our late female (>10 PCW) scRNA-seq dataset. **e**, (left) Heatmap showing the z-score enrichment of targets of clinically approved drugs (x-axis) found to be specifically impacting the epithelial compartment of late male (>10 PCW) reproductive tract organs among reproductive-specific cell types (y-axis) identified in our scRNA-seq dataset. (right) Dot plots showing the log-transformed, variance-scaled expression of the steroidogenic receptors (x-axis) and target genes (x-axis) of the clinically approved drugs in (right) in each reproductive-specific cell type (y-axis) identified in our late male (>10 PCW) scRNA-seq dataset.

To identify candidate target genes downstream of androgen signalling that may be involved in urethral canalization, we performed differential expression analysis in the early corpus spongiosum between male and female fetuses during the MPW (Fig. 4b and Methods). We identified 18 genes with male-biased expression, including the known androgen targets *CSRP2*,* CYP1B1*,* TMEM200A*,* SRD5A2* and *NID1*, as inferred by scATAC–seq data and supported by existing literature^48^ (Fig. 4c and Extended Data Fig. 8g). To determine whether these genes are conserved across species, we re-analysed a mouse scRNA-seq dataset of male and female external genitalia during the MPW^23^ and found that *Mafb* and *Csrp2* also exhibited male-biased expression in the mouse equivalent of the early corpus spongiosum (Methods and Extended Data Figs. 8h–l and 9a). By contrast, the female early corpus spongiosum displayed upregulation of genes involved in organization of the extracellular matrix (for example, *MFAP4*, *SEMA3C*, *LUM* and *FLRT2*) and *IGFBP3*, which is downregulated by androgen signalling^49^, potentially explaining its increased expression in females (Fig. 4c).

In addition to mesenchymal differences, we identified genes with sexually dimorphic expression in the human urethral epithelium. *SCGB1A1* and *PTPRD*, the most upregulated genes in male individuals (Fig. 4d), have roles in the formation of canalized epithelial structures in other tissues. That is, *SCGB1A1* has been implicated in the tubular organization of human-derived in vitro bronchioids^50^, whereas *PTPRD* is recruited to epithelial adherens junctions at the time of cell–cell contact^51^.

Knowing that mesenchymal differentiation directs epithelial differentiation^6^, we next inferred sexually dimorphic cell–cell communication events between the early corpus spongiosum and the urethral epithelium in humans. Our analysis revealed a putative male-biased interaction between *JAG1* (upregulated in the early corpus spongiosum) and its receptors *NOTCH2* and* NOTCH3* (expressed in the urethral epithelium), which implies that there is increased Notch signalling in male individuals (Extended Data Fig. 9b and Supplementary Table 9). This notion was further supported by the expression of the downstream Notch effector *HES1*. Moreover, we identified potential male-biased interactions between receptors involved in adherens junctions (*NRP1*,* NRXN3* and* PTPRD*) expressed by the urethral epithelium and their ligands (*SEMA3A*,* NLGN1*,* NLGN2*,* CLSTN1* and* LRRC4B*), which were upregulated in the early corpus spongiosum (Extended Data Fig. 9b). These findings, alongside the male-specific upregulation of *SCGB1A1* and *PTPRD* in the urethral epithelium, provide support for the key role of adherens junction signalling in enabling urethral canalization in male individuals.

Altogether, our findings shed light on the establishment of sexual dimorphism in the genital tubercle by elucidating the genes and mesenchymal–epithelial interactions that potentially mediate urethral canalization in the penis (Fig. 4e).

## Disruptions to reproductive development

**Fig. 5:**
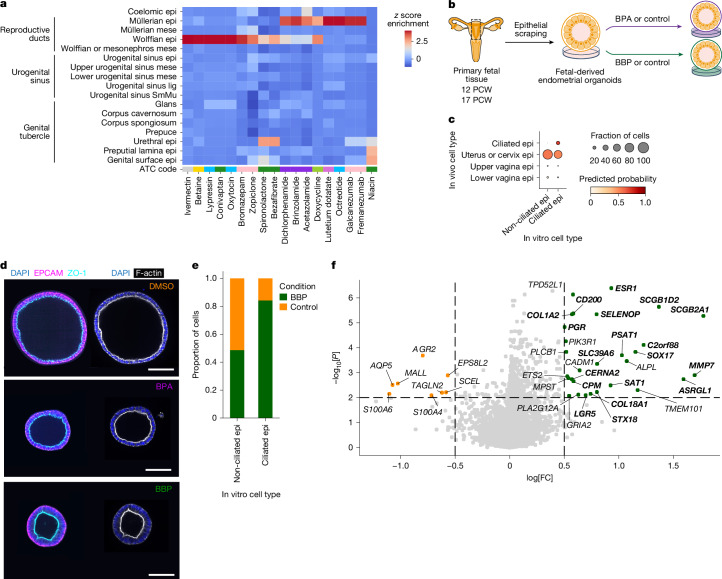
Disruptions to reproductive tract development. **a**, Heatmap showing the *z* score enrichment of targets of clinically approved drugs (*x* axis) that specifically affect the epithelial compartment of early (≤10 PCW) reproductive tract organs among reproductive-specific cell types (*y* axis) identified in our scRNA-seq dataset through drug2cell predictions. The colour of each drug represents its ATC code (details in Extended Data Fig. 9e). **b**, Schematic of the experimental design for uterine epithelial organoid derivation and exposure to the endocrine-disrupting chemicals BPA and BBP. Dimethyl sulfoxide (DMSO) was used as the control. **c**, Dot plot showing the predicted probability from each epithelial in vivo cell type (*x* axis) of the uterovaginal canal from which the organoids were derived and the non-ciliated and ciliated cells of the control organoids (*y* axis). **d**, Immunofluorescence staining of representative uterine epithelial organoids derived from a 17 PCW female fetus (Hrv277 line) at day 4 following exposure to BPA, BBP or vehicle (DMSO; Control) for EPCAM (magenta, epithelial cell marker), ZO-1 (cyan, tight junction protein indicating apicobasal polarity) and F-actin (white, cytoskeletal filament) (*n* = 2 biologically independent samples). Scale bars, 100 μm. **e**, Bar plot showing the proportion of ciliated and non-ciliated cells in fetal-derived uterine epithelial organoids treated with vehicle control (*n* = 21,905 cells) or BBP (*n*= 23,338 cells). **f**, Volcano plot showing the log fold change (*x* axis) and adjusted *P* value (*y* axis) of the differential expression of genes (adjusted *P* = 0.05, |log[FC] | > 0.5) between differentially abundant neighbourhoods in the BBP-exposed condition and all other neighbourhoods in non-ciliated G1 cells. Genes in bold are also upregulated by BPA. Illustrations in **b** created by A. García. Source data

Exogenous agents, including pharmaceutical and environmental chemicals, can interfere with developmental programs in utero and manifest as reproductive disorders later in life^52^. To identify reproductive cell types that may be susceptible to such disruptions, we focused on compounds with the potential to disrupt reproductive epithelia—a cellular compartment frequently implicated in disease—and identified 47 drugs with known anatomical therapeutic chemical (ATC) classification (Fig. 5a, Extended Data Fig. 9c–e and Supplementary Table 10).

The targets of monoclonal antibodies such as fremanezumab (which targets CALCA) and somatostatin agonists such as octreotide (which binds to SSTR2), which are used in the treatment of migraines and neuroendocrine tumours, respectively, were upregulated in the migratory Müllerian epithelium relative to all other cell types (Fig. 5a and Extended Data Fig. 9c). Conversely, the early Wolffian duct epithelium can be specifically targeted by drugs such as conivaptan (which acts on AVPR1A) and spironolactone (which targets PPARG), which are used for type 1 and 2 diabetes, respectively (Fig. 5a and Extended Data Fig. 9c). Both Müllerian and Wolffian duct epithelia, and their derivatives, particularly the uterocervical and vas deferens epithelia, also exhibited susceptibility to the antibiotic doxycycline (which binds to MMP7) (Extended Data Fig. 9c–e).

We then assessed the potential impact of EDCs, commonly found in plastics used for everyday objects, by evaluating the spatiotemporal dynamics of steroidogenic hormone receptor expression throughout gestation. Receptors for oestrogen (ESR1 and ESR2) and progesterone (PGR), the activities of which are affected by bisphenol A (BPA)^53^ and phthalate esters, androgen receptor (AR), also disrupted by phthalate esters^54^, and peroxisome proliferator-activated receptor gamma (PPARG), recently identified as a target of per- and polyfluoroalkyl substances (PFAS)^55^, are all expressed in both male and female reproductive tracts. Before 10 PCW, expression of *ESR1*,* ESR2* and *PGR* were not detected (Extended Data Fig. 9c). After 10 PCW, *ESR1* was upregulated in most Müllerian duct derivatives, in the lower vaginal epithelium and in the caput epididymis epithelium, whereas *PGR* was expressed at low levels in the ciliated fallopian tube epithelium and in the smooth muscle of the upper vagina (Extended Data Fig. 9d,e). *AR* was highly expressed in the fallopian tube epithelium, the lower vaginal mesenchyme and the epithelium of the entire epididymis after 10 PCW (Extended Data Fig. 9d,e). Upregulation of *AR* was also observed in the urogenital sinus and genital tubercle derivatives throughout gestation. *PPARG* was upregulated in the epithelial cells of the Wolffian duct early in development and later in the caudal epididymis and vas deferens, and in the urethral epithelium across all stages (Extended Data Fig. 9c–e).

**Extended Data Fig. 10:**
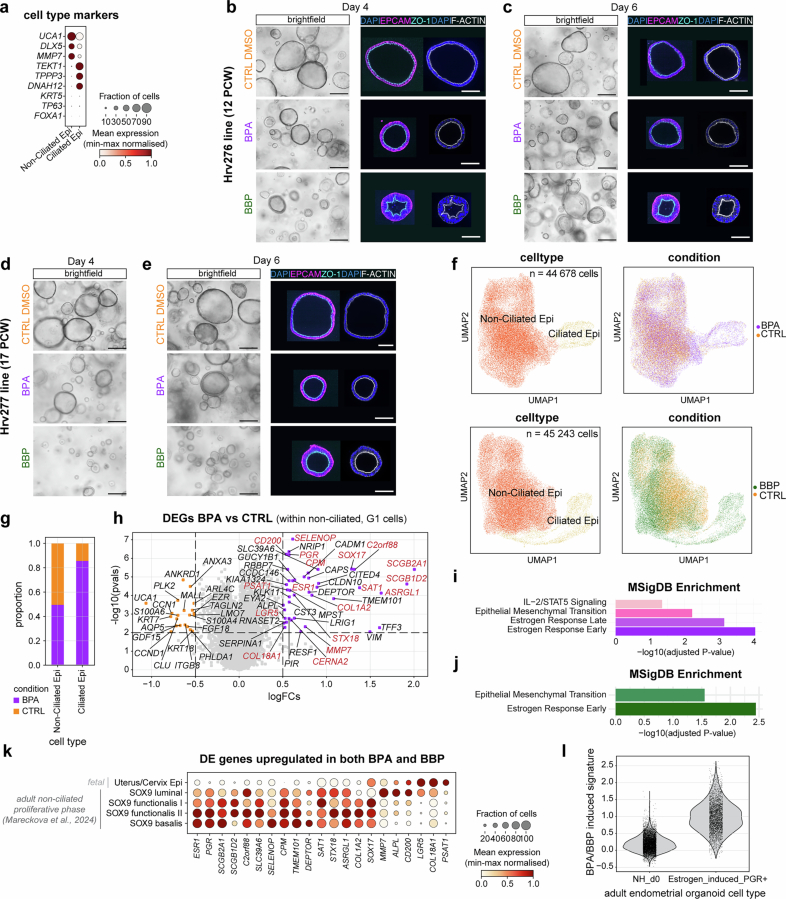
Exposure of fetal-derived uterine epithelial organoids to endocrine disrupting chemicals. **a**, Dot plot showing the log-transformed, variance-scaled expression of uterine, ciliated, and cervicovaginal epithelial markers (x-axis) in the non-ciliated and ciliated cells of the control organoids (y-axis). **b**, (left) Bright-field microscopy images of uterine epithelial organoids derived from a 12 PCW female sample at day 4 following exposure to BPA, BBP, or vehicle control (DMSO). Scale bar: 250 μm. (right) Immunofluorescence staining of representative organoids from the same experimental conditions for EPCAM (magenta, epithelial cell marker), ZO-1 (cyan, tight junction protein indicating apico-basal polarity), and F-actin (white, cytoskeletal filament) (n = 2 biologically independent samples). Scale bar: 100 μm. **c**, Same as **b**, but for the 12 PCW-derived organoid line at day 6 following exposure to BPA, BBP, or vehicle control (DMSO) (n = 2 biologically independent samples). Scale bar: 250 μm for brightfield and 100 μm for immunofluorescence. **d**, Bright-field microscopy images of uterine epithelial organoids derived from a 17 PCW female sample at day 4 following exposure to BPA, BBP, or vehicle control (DMSO) (n = 2 biologically independent samples). Scale bar: 250 μm. **e**, Same as **b**, but for the 17 PCW-derived organoid line at day 6 following exposure to BPA, BBP, or vehicle control (DMSO) (n = 2 biologically independent samples). Scale bar: 250 μm for brightfield and 100 μm for immunofluorescence. **f**, (top) Batch-corrected Uniform Manifold Approximation and Projection (UMAP) embedding of control and BPA-exposed organoids derived from 12 and 17 PCW samples profiled with scRNA-seq (n = 44,678 cells) coloured by cell type and condition. (bottom) UMAP embedding of control and BBP-exposed organoids derived from 12 and 17 PCW samples profiled with scRNA-seq (n = 45,243 cells) coloured by cell type and condition. **g**, Bar plot showing the proportion of ciliated and non-ciliated cells in control (n = 21,909 cells) and BPA-exposed (n = 22,769 cells) fetal-derived uterine epithelial organoids. **h**, Volcano plot showing the log fold-change (x-axis) and adjusted p-value (y-axis) of the differential expression test (adjusted p-value = 0.05, |logFC| > 0.5) between differentially abundant neighbourhoods in the BPA exposed condition and all other neighbourhoods. Genes colored in red are also upregulated by BBP. **i**, Gene Set Enrichment Analysis of the upregulated genes upon BPA exposure. **j**, Gene Set Enrichment Analysis of the upregulated genes upon BBP exposure. **k**, Dot plot showing the variance-scaled, log-transformed expression of the genes (x-axis) which are upregulated by both BPA and BBP in non-ciliated cells (y-axis) in in vivo fetal uterine epithelium and adult uterine epithelium in the proliferative phase of the menstrual cycle^104^. **l**, Violin plot showing the combined expression of genes upregulated by both BPA and BBP identified in fetal-derived uterine organoids in the adult endometrial organoids before (NH_d0) and after (Estrogen_induced_PGR+) the addition of estrogen in the media. Source data

To experimentally validate these predictions, we tested the effects of BPA and benzyl butyl phthalate (BBP), a representative phthalate ester, on fetal uterine epithelial organoids derived from 12 and 17 PCW fetuses (Fig. 5b–d and Extended Data Fig. 10a–f). Label transfer from in vivo fetal data (Fig. 5c) and canonical marker expression of fetal uterine cells (Extended Data Fig. 10a) confirmed the fetal uterine identity of the organoids before exposure to the chemicals. Under basal conditions, the fetal uterine epithelium expressed *ESR1* but lacked expression of *PGR* (Extended Data Fig. 9d), which suggested that either systemic oestrogen levels were too low to activate *ESR1* or that *ESR1*-expressing cells in the fetal uterus are not yet competent to respond to oestrogen signalling. Following exposure to BPA and BBP, we observed an increase in ciliated cells in response to both compounds (Fig. 5e and Extended Data Fig. 10g), a result consistent with previous reports that oestrogen promotes ciliogenesis in female reproductive tissues^56^. Moreover, the organoids showed upregulation of *PGR* and several well-characterized oestrogen-responsive genes (for example, *SCGB2A1*,* SCGB1D2*,* ASRGL1* and* SLC39A6*)^57–60^ (Fig. 5f and Extended Data Fig. 10h–j). Finally, we used the BPA-induced and BBP-induced gene signature to score adult endometrial organoids from our previous study^61^. There was higher enrichment in oestrogen-treated organoids than in oestrogen-deprived controls, a finding that further supports the oestrogen-like effects of these EDCs^58^ (Extended Data Fig. 10k,l).

In summary, our integrated atlas enabled us to predict when and where external agents, such as clinically approved drugs and EDCs, have the potential to act during gestation.

## Discussion

Congenital reproductive-tract disorders affect more than 3% of female^4^ and 0.8% of male^5^ newborns; however, our understanding of prenatal reproductive-tract development remains limited. In this study, we generated a developmental roadmap of the male and female reproductive tracts during key periods of sexual differentiation. This roadmap provides detailed temporal and spatial distributions of 52 reproductive-tract-specific cell types in 89 human samples spanning 6–21 PCW. Leveraging this dataset, we uncovered sex-specific cues that drive the divergent development of reproductive organs and the selective regression of sexually unmatched ducts. Moreover, by characterizing the progressive differentiation of epithelial and surrounding mesenchymal compartments, we provide new cellular and molecular insights into how early axial gradients are translated into defined cell lineages and distinct tissue structures (Supplementary Note 5). With this resource, researchers can contextualize known genetic variants linked to reproductive diseases by identifying when and in which cell types genes are expressed or chromatin regions are open. Moreover, our findings about gradients of transcription factors and morphogens activated during reproductive development pave the way for generating more complex in vitro models, which may facilitate the study of disease-causing perturbations.

## Methods

### Samples

Fetuses were obtained after voluntary terminations of pregnancy, which were performed either via medical or surgical procedures. The termination methods used did not compromise the integrity or morphology of the fetuses analysed in this study. Only well-preserved fetuses, without evidence of structural damage, were included. All tissue samples used for this study were obtained with written informed consent from all participants in accordance with the guidelines in The Declaration of Helsinki 2000. The human embryonic and fetal material was provided by the Joint MRC–Wellcome Trust (grant number MR/R006237/1 and MR/X008304/1) Human Developmental Biology Resource (HDBR, http://www.hdbr.org), with appropriate maternal written consent and approval from the Fulham Research Ethics Committee (REC reference 18/LO/0822 and 23-LO/0312) and Newcastle & North Tyneside 1 Research Ethics Committee (REC reference 18/NE/0290). The HDBR is regulated by the UK Human Tissue Authority (HTA; www.hta.gov.uk) and operates in accordance with the relevant HTA Codes of Practice. This research was also supported by the NIHR Cambridge Biomedical Research Centre (NIHR203312). The views expressed are those of the authors and not necessarily those of the NIHR or the Department of Health and Social Care.

### Assignment of developmental stage

Embryos up to 8 PCW were staged using the Carnegie staging method^62^. At stages beyond 8 PCW, age was estimated from measurements of foot length and heel-to-knee length and compared with the standard growth chart^63^. A piece of skin or, where this was not possible, chorionic villi tissue was collected from every sample for quantitative PCR analyses using markers for the sex chromosomes and the autosomes 13, 15, 16, 18, 21 and 22, which are the most commonly seen chromosomal abnormalities. All samples were karyotypically normal.

### Tissue processing

All tissues for sequencing and spatial work were collected in HypoThermosol FRS Preservation solution (Sigma-Aldrich) and stored at 4 °C until processing. Tissue dissociation was conducted within 24 h of tissue retrieval with the exception of tissues that were cryopreserved and stored at −80 °C (Supplementary Table 1).

We used a previously described protocol optimized for gonadal dissociation and available at protocols.io^64^. In brief, tissues were cut into <1 mm^3^ segments before digestion with a mix of trypsin–EDTA 0.25% and DNase I (0.1 mg ml^–1^) for 5–15 min at 37 °C with intermittent shaking. Samples >17 PCW were digested using a combination of collagenase and trypsin–EDTA using a previously described protocol^64,65^, but with modifications. In brief, samples were first digested with a mix of collagenase 1A (1 mg ml^–1^), DNase I (0.1 mg ml^–1^) and Liberase TM (50 µg ml^–1^) for 45 min at 37 °C with rotation. The cell solution was further digested with trypsin–EDTA 0.25% for 10 min at 37 °C with rotation. In both protocols, digested tissue was passed through a 100 µm filter and cells were collected by centrifugation (500*g* for 5 min at 4 °C). Cells were washed and resuspended in PBS–BSA 0.04% before cell counting.

### Single-nucleus suspension

Single-nucleus suspensions were isolated from dissociated cells when performing scATAC–seq, following the manufacturers’ instructions, and from frozen tissue sections when performing multi-omic snRNA-seq and scATAC–seq. For the latter, thick (300 µm) sections were cryosectioned and kept in a tube on dry ice until subsequent processing. Nuclei were released by Dounce homogenization as described in detail in the methods at protocols.io (10.17504/protocols.io.bp2l6n1xkgqe/v1).

### Tissue cryopreservation

Fresh tissue was cut into <1 mm^3^ segments before being resuspended with 1 ml ice-cold Cryostor solution (CS10, C2874-Sigma). Tissue was frozen at −80 °C, decreasing the temperature approximately 1 °C min^–1^. A detailed protocol is available at protocols.io (10.17504/protocols.io.bgsnjwde).

### Tissue freezing

Fresh tissue samples of the human developing reproductive tract were embedded in cold OCT medium and flash-frozen using a dry ice–isopentane slurry.

### H&E staining and imaging

Fresh-frozen sections were removed from −80 °C storage and air dried before being fixed in 10% neutral-buffered formalin for 5 min. After rinsing with deionized water, slides were dipped in Mayer’s haematoxylin solution (QPath) for 90 s. Slides were completely rinsed in 4–5 washes of deionized water, which also served to blue the haematoxylin. Aqueous eosin 1% (Leica) was manually applied onto sections with a pipette and rinsed with deionized water after 1–3 s. Slides were dehydrated through an ethanol series (70%, 70%, 100% and 100%) and cleared twice in 100% xylene. Slides were coverslipped and allowed to air dry before being imaged on a Hamamatsu Nanozoomer 2.0HT digital slide scanner.

### Multiplexed smFISH and high-resolution imaging

Large-tissue section staining and fluorescence imaging were conducted largely as previously described^66^. Sections were cut from fresh-frozen or fixed-frozen samples embedded in OCT at a thickness of 10 μm using a cryostat, placed onto SuperFrost Plus slides (VWR) and stored at −80 °C until stained. Tissue sections were then processed using a Leica BOND RX to automate staining with a RNAscope Multiplex Fluorescent Reagent kit v2 assay (Advanced Cell Diagnostics, Bio-Techne) according to the manufacturers’ instructions. Details of the probes used are provided in Supplementary Table 3. Before staining, human fresh-frozen sections were post-fixed in 4% paraformaldehyde in PBS for 15 min at 4 °C, then dehydrated through a series of 50%, 70%, 100% and 100% ethanol for 5 min each. Following manual pretreatment, automated processing included epitope retrieval by protease digestion with protease IV for 30 min before probe hybridization. Subsequently, the automated processing for these sections included heat-induced epitope retrieval at 95 °C for 5 min in buffer ER2 and digestion with protease III for 15 min before probe hybridization. Tyramide signal amplification with Opal 520, Opal 570 and Opal 650 (Akoya Biosciences) and TSA-biotin (TSA Plus Biotin kit, Perkin Elmer) and streptavidin-conjugated Atto 425 (Sigma Aldrich) was used to develop RNAscope probe channels.

Stained sections were imaged with a Perkin Elmer Opera Phenix High-Content Screening system in confocal mode with 1-μm *z* step size, using a ×20 (NA 0.16, 0.299 μm pixel^–1^), ×40 (NA 1.1, 0.149 μm pixel^–1^) or ×63 (NA 1.15, 0.091 μm pixel^–1^) water-immersion objective. The following channels were used: DAPI, excitation (ex.) of 375 nm and emission (em.) of 435–480 nm; Atto 425, ex. of 425 nm and em. of 463–501 nm; Opal 520 ex. of 488 nm and em. of 500–550 nm; Opal 570, ex. of 561 nm and em. of 570–630 nm; and Opal 650, ex. of 640 nm and em. of 650–760 nm.

### Image stitching

Confocal image stacks were stitched as two-dimensional maximum-intensity projections using proprietary Acapella scripts provided by Perkin Elmer.

### 10x Genomics Chromium GEX library preparation and sequencing

For the scRNA-seq experiments, cells were loaded according to the manufacturer’s protocol for the Chromium Next GEM Single Cell 5′ v2 (DUAL) kit (10x Genomics) to attain between 2,000 and 10,000 cells per reaction. Library preparation was carried out according to the manufacturer’s protocol. Libraries were sequenced, aiming at a minimum coverage of 20,000 raw reads per cell, on Illumina HiSeq 4000 or Novaseq 6000 systems using the following sequencing format: read 1, 26 cycles; i7 index, 8 cycles; i5 index, 0 cycles; read 2, 98 cycles.

For the scATAC–seq and multimodal snRNA-seq and scATAC–seq experiments, cells were loaded according to the manufacturer’s protocol for the Chromium Single Cell ATAC v2 and Chromium Next GEM Single Cell Multiome ATAC+Gene Expression (10x Genomics) to attain between 2,000 and 10,000 cells per well. Library preparation was carried out according to the manufacturer’s protocol. Libraries for scATAC–seq were sequenced on an Illumina NovaSeq 6000 system, aiming at a minimum coverage of 10,000 fragments per cell, with the following sequencing format: read 1, 50 cycles; i7 index, 8 cycles; i5 index, 16 cycles; read 2, 50 cycles.

### 10x Genomics Visium library preparation and sequencing

Cryosections (10 µm) were cut and placed on Visium slides. These were processed according to the manufacturer’s instructions. In brief, sections were fixed with cold methanol, stained with H&E and imaged on a Hamamatsu NanoZoomer 2.0HT before permeabilization (24–30 min), reverse transcription and cDNA synthesis using a template-switching protocol. Second-strand cDNA was liberated from the slide and single-indexed libraries were prepared using a 10x Genomics PCR-based protocol. Libraries were sequenced (1 per lane on a HiSeq4000), aiming for 300 million raw reads per sample, with the following sequencing format: read 1, 28 cycles; i7 index, 8 cycles; i5 index, 0 cycles; read 2, 91 cycles.

### 10x Genomics Visium CytAssist library preparation and sequencing

Cryosections (10 µm) were collected onto SuperFrost Plus slides (VWR) and processed according to the 10x CytAssist protocol (CG000614 and CG000495). In brief, sections were fixed in methanol, H&E stained and imaged on a Hamamatsu Nanozoomer 2.0HT. After destaining, human whole transcriptome probe pairs were hybridized and ligated to the tissue RNA. The ligation products were then released and captured onto Visium slides using a CytAssist instrument. The probes were then extended to incorporate the spatial barcodes from the Visium slide, eluted and prepared into a dual-indexed library. Libraries were sequenced (4 samples per Illumina Novaseq SP flow cell) aiming for a minimum 25,000 read pairs per spot, with the following sequencing format: read 1, 28 cycles; i7 index, 10 cycles; i5 index, 10 cycles; read 2S, 90 cycles.

### Customized ISS pipeline

ISS was performed using a 10x Genomics CARTANA HS Library Preparation kit (1110-02, following protocol D025) and an In Situ Sequencing kit (3110-02, following protocol D100), which comprise a commercialized version of HybISS^67^. A reproductive tract section was fixed in 3.7% formaldehyde (Merck 252549) in PBS for 30 min, washed twice in PBS for 1 min each, permeabilized in 0.1 M HCl (Fisher 10325710) for 5 min and washed twice again in PBS, all at room temperature. Following dehydration in 70% and 100% ethanol for 2 min each, a 50, 100 or 150 μl volume (depending on the size of the section) SecureSeal hybridization chamber (Grace Bio-Labs GBL621505-20EA) was adhered to the slide and used to hold subsequent reaction mixtures. Following rehydration in buffer WB3, probe hybridization in buffer RM1 was conducted for 16 h at 37 °C. The 171-plex probe panel included 5 padlock probes per gene, the sequences of which are proprietary (10x Genomics CARTANA). The section was washed with PBST (PBS with 0.05% Tween-20) twice, then with buffer WB4 for 30 min at 37 °C, and thrice again with PBST. Probe ligation in RM2 was conducted for 2 h at 37 °C and the section washed thrice with PBST, then rolling circle amplification in RM3 was conducted for 18 h at 30 °C. Following PBST washes, all rolling circle products (RCPs) were hybridized with LM (Cy5 labelling mix with DAPI) for 30 min at room temperature, the section was washed with PBST and dehydrated with 70% and 100% ethanol. The hybridization chamber was removed and the slide mounted with SlowFade Gold Antifade mountant (Thermo, S36937).

Imaging of Cy5-labelled RCPs at this stage acted as a quality-control step to confirm RCP (‘anchor’) generation and served to identify spots during decoding. Imaging was conducted using a Perkin Elmer Opera Phenix Plus High-Content Screening system in confocal mode with 1-μm *z* step size using a ×63 (NA 1.15, 0.097 μm pixel^–1^) water-immersion objective and 7% overlap between adjacent tiles. The following channels were used: DAPI, ex. of 375 nm and em. of 435–480 nm); Atto 425, ex. of 425 nm and em. of 463–501 nm; Alexa Fluor 488, ex. of 488 nm and em. of 500–550 nm; Cy3, ex. of 561 nm and em. 570–630 nm; and Cy5, ex. of 640 nm and em. of 650–760 nm.

Following imaging, the slide was de-coverslipped vertically in PBS (gently, with minimal agitation such that the coverslip fell off to prevent damage to the tissue). The section was dehydrated with 70% and 100% ethanol, and a new hybridization chamber was secured to the slide. The previous cycle was stripped using 100% formamide (Thermo AM9342), which was applied fresh each minute for 5 min, then washed with PBST. Barcode labelling was conducted using two rounds of hybridization: first, with an adapter probe pool (AP mixes AP1–AP6, in subsequent cycles), then a sequencing pool (SP mix with DAPI, customized with Atto 425 in place of Alexa Fluor 750), each for 1 h at 37 °C with PBST washes in between and after. The section was dehydrated, the chamber removed and the slide mounted and imaged as described above. This process was repeated another five times to generate the full dataset of seven cycles (anchor and six barcode bits).

### Derivation and maintenance of fetal uterine organoids

Fetal uterine organoids were derived from 12 PCW (Hrv276-ORG) and 17 PCW (Hrv277-ORG) fetal reproductive tract samples, (developing uterus, cervix and vagina) following tissue dissociation as described above. The single-cell suspensions were washed in Advanced DMEM/F12 (Gibco, 12634-010), centrifuged and the cell pellet resuspended in Matrigel (Corning, 356231) at around a 1:3 ratio (pellet volume to Matrigel volume). The organoids were cultured as previously described^68^, forming in 25 µl Matrigel domes in 48-well tissue treated plates covered by 250 µl basal endometrial organoid medium (Advanced DMEM/F12 (Gibco, 12634-010), 1% GlutaMAX (Gibco, 35050061), 1% insulin–transferrin–selenium (ITS-G) (Gibco, 41400045), 100 µg ml^–1^ primocin (Invivogen, ant-pm-1), 1× B27-vitamin A (Life Technologies, 12587010), 1× N2 (Life Technologies, 17502048), 1.25 mM *N*-acetyl-l-cysteine (Sigma-Aldrich, A9165-5G), 2 mM nicotinamide (Sigma, N0636-100G), 2 ng ml^–1^ recombinant human FGF-basic (154 amino acids) (Peprotech, 100-18B), 500 ng ml^–1^ recombinant human R-spondin-1 (R&D Systems, 4645-RS-01M/CF), 10 µM SB202190 (p38i) (StemCell Technologies, 72632), 500 nM A83-01 (Tocris, 2939), 50 ng ml^–1^ recombinant human EGF (Peprotech, AF-100-15), 10 ng ml^–1^ recombinant human FGF-10 (Peprotech, 100-26) and 100 ng ml^–1^ recombinant human Noggin (Peprotech, 120-10 C))^68^. The medium was supplemented with 10 µM of the ROCK inhibitor Y-27632 (Millipore, 688000) for the first 2 days of organoid line establishment, with full medium changes every 2–3 days.

All organoid lines were split and passaged approximately every 5–7 days depending on their size and density. TrypLE Express Enzyme (Gibco, 12604013) was added to each well, and domes were detached with either a 1,000 µl tip or cell scraper before being transferred to a 15 ml Falcon tube. The organoids were dissociated into cell clumps by forcefully pipetting the solution 15–30 times using a 1,000 µl tip, followed by incubation at 37 °C for 6–8 min. Advanced DMEM/F12 was added at 1:1 ratio to quench TrypLE Express Enzyme and pipetted up and down 10 more times with the 1,000 µl tip. Cell suspensions were centrifuged at 800*g* for 2 min at 4 °C. The supernatant was removed as close to the pellet as possible. Next, 30 µl cold Matrigel per desired dome were added and the pellet was slowly resuspended to evenly distribute the cells. A volume of 30 µl domes was seeded into 6-well, 12-well or 24-well tissue culture-treated plates depending on whether the organoids were being expanded or set-up for drug treatment. The domes were placed in an incubator for 10 min at 37 °C, followed by the addition of basal endometrial organoid medium supplemented with 10 µM Y-27632.

### Treatment of fetal uterine organoids with BPA or BPP

For all drug treatment experiments, organoids were passaged 48 h before addition of the compound. Organoids were plated in 30 µl domes as described above in two technical replicates, each containing two organoid domes. For testing the effect of endocrine disruptors on fetal reproductive organoids, Hrv276-ORG and Hrv277-ORG lines were treated with either 10 μM of BPA (Sigma, 239658) or 100 μM BBP (Sigma, 308501), with controls receiving the same volume of DMSO as the compound administered. For the endocrine-disrupter experiments BPA, BBP or DMSO were added to basal endometrial organoid medium (as described above), but with phenol-red-free DMEM/F12 as the base medium (Merck, D6434). Organoids were treated with BPA or BBP for a total of 96 or 144 h, with full medium change every 48 h. After 96 or 144 h of drug treatment, organoids were dissociated into a single-cell suspension. In brief, organoids were collected and washed in ice-cold phenol-red-free DMEM/F12 (BPA, BBP or DMSO control) and centrifuged at 600*g* for 6 min at 4 °C. The supernatant was removed and replaced with TrypLE Express Enzyme at a ratio of 500 µl per 30 µl dome, and pipette-mixed with a p1,000 tip for 30 times. Organoid suspensions were incubated at 37 °C for 15–25 min, with manual shaking every 2 min. Cells were checked at 15 min then every 5 min until an adequate single-cell suspension was achieved of about 70% single cells. Once the cells were sufficiently digested, TrypLE was quenched with phenol-red-free DMEM/F12. Cells were re-centrifuged and medium was aspirated to leave around 50 µl of medium. The cell suspensions were then vigorously pipette-mixed with a p20 tip 30–60 times. To this suspension, 200 μl of 1% PBS–BSA was added, thoroughly mixed and passed through a 70 μm filter. Live cells were counted using Trypan blue. The cells were loaded into a 10x Genomics Chromium chip as described in the Chromium Next GEM Single Cell 5′ v2 (DUAL) kit.

### Immunofluorescence of fetal uterine organoids

Fetal uterine organoids were grown and treated as described above in µ-Slide 8 Wells (ibidi, 80801). Organoids were fixed in 4% paraformaldehyde for 45 min at room temperature and washed several times in PBS. Cells were permeabilized and blocked for 2 h in 2% Triton-X + 5% FBS in PBST. Organoids were washed in PBS before incubation with primary antibodies. Antibodies were incubated in an antibody dilution buffer (0.25% Triton-X + 1% FBS in PBST) at 4 °C overnight. Organoids were stained with TRITC-conjugated phalloidin (Thermo Fisher Scientific, R37112, dilution according to the manufacturer’s instructions), Alexa 488-conjugated ZO-1 (Invitrogen, 339188; 1:200 dilution) and APC-conjugated EpCAM (BD biosciences, 347200; 1:200 dilution). Organoids were washed 3 times for 45 min each in PBS and then mounted in ibidi mounting medium (ibidi, 50011).

Organoids were imaged with a Perkin Elmer Opera Phenix High-Content Screening system in confocal mode with 10-μm *z* step size, using a ×20 (NA 0.16, 0.299 μm pixel^–1^) water-immersion objective. The following channels were use: DAPI, ex. of 375 nm and em. of 435–480 nm); Alexa 488, ex. of 488 nm and em. of 500–550 nm; TRITC, ex. of 561 nm and em. of 570–630 nm; and APC, ex. of 640 nm and em. of 650–760 nm.

### Analysis of scRNA-seq data

#### Per-library analyses

For each sequenced scRNA-seq library, we performed read alignment to the human reference genome (GRCh38 2020-A), and mRNA quantification and initial quality control using STARsolo^69^ with default parameters. Ambient RNA contamination was inferred and subtracted from the original expression matrix using the deep generative model CellBender^70^. For multiplexed libraries, Souporcell^71^ was then applied to deconvolve the genotypes and to assign cells to their respective donors. Owing to the scarcity of human cell-type markers, each library was first analysed independently before integrating them to have a way of formally evaluating the quality of the integration. In brief, we used Scrublet^72^ for cell-doublet calling with a two-step diffusion doublet identification, as previously described^73^. Genes expressed by fewer than 3 cells were excluded, as were cells expressing fewer than 1,500 genes, more than 20% mitochondrial genes or with more than 40% of the scrublet score.

After converting the expression space to log [CPM/100 + 1], the anndata object was transposed to the gene space to identify cell cycling genes in a data-driven manner, as previously described^73,74^. Principal component analysis, neighbour identification and partition-based Leiden clustering^75^ were performed on the gene space, and then the members of the gene cluster, including known cycling genes (*CDK1*, *MKI67*, *CCNB2* and *PCNA*), were flagged as the data-derived cell cycling genes and discarded in the downstream analysis. Back in the cell space, we identified highly variable genes, performed principal component analysis, computed the neighbourhood graph, Leiden clustering^75^ and UMAP^76^ for visualization in two dimensions. The per-library computational analysis workflow described so far was wrapped in a Nextflow^77^ pipeline with two processes to enable parallelization and reproducibility.

To identify genes characteristic of each cluster, we performed term frequency–inverse document frequency, a method borrowed from natural-language processing that reflects how important a word (gene) is to a document (cluster) in a corpus (dataset), as implemented in the R library SoupX^78^. Annotations were only finalized when analysing spatially resolved transcriptomics data (both 10x Visium and ISS). A detailed explanation of the cell types identified alongside their marker genes is provided in Supplementary Note 1.

#### Integration of scRNA-seq libraries

After having annotated each sample separately and realizing the significant differences in cell-type composition across samples, we generated three integrated views that best preserved the biological heterogeneity of this system: ≤10 PCW male and female samples together (when the sexually unmatched ducts are still present and the first signs of regionalization of the ducts appear); >10 PCW male samples; >10 PCW female samples. The variational autoencoder-based method scVI^79^, trained on the 7,500 most highly variable genes and with 30 latent variables and 2 hidden layers, was then applied to mitigate batch effects across donors in each of the three views. To evaluate the integrated manifold, we then overlaid the per-sample annotations and confirmed that the biological signal was preserved while correcting for donor-specific effects. Moreover, for a more quantitative evaluation of the integration results, we computed the Shannon entropy per Leiden cluster of the per-sample cell-type annotations as well as the donor and sex labels, following a previously described method^80^. Clusters with a donor label entropy equal to 0 (that is, donor-specific clusters) were removed from further analysis. Each cluster was then annotated on the basis of majority voting (≥40%) of the cell-type labels. Clusters showing high cell-type label entropy (that is, <40% of cells in the cluster having the same cell-type label) were further investigated and annotated according to their most expressed term frequency–inverse document frequency markers.

Finally, for visualization purposes only, all libraries were integrated with scVI (7,500 highly variable genes, 60 latent variables), and cell-type labels were overlaid from the per-view annotations. Variations in cell-type proportions across developmental time were visualized with an area plot.

#### Per-organ analyses

The cellular and molecular features of the establishment of sexual dimorphism in each organ of the developing human reproductive tract were investigated by performing subanalyses on the following relevant cell types:

- All cell types in male and female external genitalia during the MPW (8–14 PCW)
- Coelomic epithelium, Müllerian duct epithelium and mesenchyme during the period of Müllerian duct emergence (6–8 PCW)
- Mesenchymal and epithelial cells from the differentiating Müllerian and Wolffian ducts (>10 PCW), resulting in four subanalyses (Müllerian-derived mesenchyme, Müllerian-derived epithelium, Wolffian-derived mesenchyme and Wolffian-derived epithelium).

In all these per-organ analyses, preprocessing was carried out analogously to what is described in the per-library analysis section, and Harmony^81^ (theta = 0) was used to correct for batch effects.

#### Human–mouse comparison of external genitalia

We leveraged the availability of an annotated scRNA-seq dataset of mouse genital tubercle (comprising two male and female samples for each of three developmental stages: embryonic day 14.5 (E14.5), E16.6 and and E18.5)^23^ to identify the transcriptional regulators underpinning sexual dimorphism in the corpus spongiosum in each species. To define a shared feature space, we first took the set of orthologous genes expressed in at least ten cells in both species. From the set of common orthologous genes, we then computed the top 4,000 highly variable genes in each species and identified their intersection (around 2,700 genes). Using the intersection of highly variable genes, batch effects owing to different donors or mice were corrected by means of Harmony^81^, and a Milo^82^ object was computed on each species’ batch-corrected embedding. Each neighbourhood from a species was matched to its *k* (*k* = 30) closest neighbourhoods in the other species (in both the mouse-to-human and human-to-mouse directions). The final matches were formed by identifying the mutually nearest pairs of neighbourhoods that appeared in both directions^83^. Each matched neighbourhood was then annotated by majority voting of the labels of the cells in the neighbourhood. Matching across cell-type labels was visualized with an alluvial plot. We then considered the unique combination of matching cell-type labels as the harmonized cell-type annotations across species and focused on the mouse cells that matched the human corpus spongiosum label for differential expression analysis between male and female individuals.

#### Differential expression analysis in the human and mouse genital tubercle

We conducted differential expression analysis between male and female samples in the human and mouse corpus spongiosum and human urethral epithelium using PyDESeq2^84^. Only samples between 8 and 14 PCW (the so-called MPW) were included in the analysis. Genes mapping to the Y chromosome were excluded from differential expression testing. Results of the differential expression analysis were visualized with a Volcano plot showing the genes with |log[FC > 1]| and adjusted *P* < 0.05.

#### Cell–cell interaction analysis from scRNA-seq data

Sexually dimorphic cell–cell interactions between the mesenchymal corpus spongiosum and the urethral epithelium during the MPW (8–14 PCW) were inferred using CellphoneDB (v.5.1)^85^ using method 3 (differential expression analysis). After splitting both cell clusters into male and female, differentially expressed genes for each cell type–sex combination were identified using the FindAllMarkers() function in Seurat^86^, with only positive markers being considered. Only genes expressed in at least 10% of the cells in a cell type–sex combination were considered for this analysis. The search for interaction was restricted to cell types in the same sex (which was passed as input to the method in the ‘microenvironments’ file).

Wolffian-to-Müllerian duct signalling during the developmental time window of Müllerian duct emergence (6–8 PCW) was also explored with CellphoneDB (v.5.1)^85^ using method 3. Differentially expressed genes per cell type (that is, Wolffian mesenchyme, Wolffian epithelium, Müllerian mesenchyme and Müllerian epithelium) were similarly computed using the FindAllMarkers() function in Seurat^86^ (with only positive markers being considered), and only genes expressed in at least 10% of the cells in a cell type were considered for this analysis. All cell types belong to the same ‘microenvironment’.

#### Trajectory inference and differential expression along trajectories

The trajectory inference method Slingshot^87^ was applied to recover the lineages originating from the coelomic epithelium during Müllerian duct emergence (6–8 PCW). The pseudotime ordering of the cells along with the weighted assignment of each cell to the three lineages were then used as input for TradeSeq^88^ to extract genes that were differentially expressed along a lineage or between lineages with the associationTest() function.

#### Inference of clinically approved drugs potentially disrupting reproductive epithelia

Using drug2cell^89^, we derived drug scores for compounds in the CHEMBL database by averaging the expression levels of target genes in each cell on the three views of the dataset independently. We then performed a Wilcoxon rank-sum test to identify significant differences in drug scores between each reproductive epithelium (that is, Müllerian duct epithelium, Wolffian duct epithelium, urogenital sinus epithelium and urethral epithelium in <10 PCW female and male embryos; fallopian tube epithelium, uterocervix epithelium, upper vagina epithelium, vaginal plate epithelium and urethral epithelium in >10 PCW female fetuses; epididymis epithelium, vas deferens epithelium, prostate epithelium and urethral epithelium in >10 PCW fetuses) and all other reproductive-specific cells in the dataset. Results were filtered based on adjusted *P* values (<0.01), log fold changes (>2) and rank scores to select the most significant drugs associated with the target cell type. An additional filtering step was performed to exclude drugs for which target genes were not specific to the target cell type and required that the targets were expressed in at least 10% of cells in the target cell type.

#### In vivo–in vitro comparison

In vivo epithelial cells from the uterovaginal canal were used to train a CellTypist^90^ model with the top 200 genes per cell type (as ranked by their absolute regression coefficients associated with each cell type) as features. The trained model was then used to predict the cell-type labels in the untreated organoids, and the predicted probabilities were visualized using a dot plot.

To assess changes in cellular abundance following perturbation, Milo^82^ was used to construct a *k*-nearest neighbour (kNN) graph on embeddings integrated using Harmony^81^ for untreated versus BPA-treated and untreated versus BBP-treated organoids. Differential abundance testing was performed by assigning cells to neighbourhoods and applying a generalized linear model to compare the proportion of BPA-treated or BBP-treated cells relative to untreated controls, accounting for differences in cellular sampling. Differential expression analysis was conducted by comparing transcriptomic profiles of cells in differentially abundant neighbourhoods to all remaining neighbourhoods in each dataset. Genes with |log[FC]| > 0.5 and adjusted *P* < 0.05 were considered significant, and results were visualized using a volcano plot.

Gene set enrichment analysis was performed using EnrichR^91^ to identify biological pathways associated with BPA-induced or BBP-induced gene expression changes. Upregulated genes were compared against the MSigDB Hallmark 2020^92^ gene sets, and significantly enriched pathways (adjusted *P* < 0.05) were visualized using a bar plot.

### Analysis of scATAC–seq data

#### Per-library analyses

scATAC–seq libraries were processed (read filtering, alignment, barcode counting and cell calling) with 10x Genomics Cell Ranger ATAC pipeline v.2 using the pre-built 10x GRCh38 genome (v.3.1.0) as a reference. Similar to RNA, we analysed each ATAC library independently until cell-type annotation to evaluate the quality of the subsequent integrations using the ArchR framework^93^. Cells with fewer than 3,500 fragments or a minimum transcription start site enrichment of 10 were filtered out, as were cells deemed as doublets. Dimensionality reduction on the tile matrix was performed with Latent Semantic Indexing, and the low-dimensional variables were then used to compute the neighbourhood graph, partition-based Leiden clustering^75^ and UMAP visualization^76^.

To annotate cells, we used canonical correlation analysis to match the gene activity score matrix of each scATAC–seq library with the integrated gene-expression space of the corresponding view (for example, if the scATAC–seq library came from a female sample older than 10 PCW, we used the >10 PCW female-integrated scRNA-seq dataset). For optimal reproducibility and parallelization, the per-sample scATAC–seq analyses were also wrapped in a Nextflow script.

#### Integration of scATAC–seq libraries

The individually annotated samples were then integrated by re-computing latent semantic indexing on the concatenated tile matrices and using mutual nearest neighbours^83^ to correct for batch effects. Mutual nearest neighbours has proven to be highly effective for scATAC–seq (for which we do not have as many samples as scRNA–seq), as it enables us to specify the order of integration and hence mitigate batch effects in a more biologically informed fashion.

#### Integrative analysis of scRNA-seq and scATAC–seq data

Combining information from transcriptomics and chromatin accessibility data enables the prioritization of transcription factors that are likely to be active in each cell type, along with the identification of putative regulatory relationships between transcription factors and target genes. We sought to do this when investigating the regulatory landscape underlying the process of urethral canalization in males, Müllerian duct emergence from the coelomic epithelium, and patterning of the mesenchyme and epithelium during Müllerian and Wolffian duct differentiation.

Using the fragment files and cell annotations obtained through label transfer from the scRNA-seq data, pseudobulks were generated per cell type. Cell-type-specific peaks were called using MACS2^94^ as implemented in the pycistopic workflow^95^. A set of consensus peaks was derived through iterative overlapping, and the resulting matrix of cells by consensus peaks was used as input to topic modelling with latent dirichlet allocation. Latent dirichlet allocation models were selected according to the metrics provided in pycistopic. Harmony^81^ was used to correct for the donor effect. The manifold was clustered with the Leiden algorithm^75^ and differentially accessible regions were computed between the Leiden clusters. The union of the differentially accessible regions and the regions contained in topics (obtained by binarizing the region-topic probabilities) served as the set of regions used to find transcription factor-binding motifs with pycistarget^95^.

To infer enhancer-driven gene regulatory networks, scRNA-seq and scATAC–seq data were eventually combined into a pseudo-multiome dataset using SCENIC+^95^. In essence, SCENIC+ generates metacells that contain cells from both data modalities by randomly sampling cells of the same cell-type label. Within a 150-kb region around each gene, gradient-boosting machines and correlation analysis were used to infer the relationships between enhancers and genes, as well as between transcription factors and genes. This approach enabled the identification of enhancers that are associated with the regulation of specific target genes and the inference of transcription factors that potentially contribute to the regulation of these genes.

### Analysis of 10x Visium data

#### Per-library analyses

Visium data consist of FASTQ sequencing files and a bright-field microscopy image stained with H&E per capture area. The Space Ranger (v.2.0.0) software provided by 10x Genomics was used to align the barcoded spot pattern to the H&E tissue image and to differentiate tissue from background. The resulting spot-by-transcript abundance matrix was analysed using the package squidpy.

#### Estimation of cell-type abundances per Visium spot using matched scRNA-seq data

To deconvolve the transcriptional signal coming from each Visium spot into an estimated abundance of each cell type present in the >10 PCW female and male views of the scRNA-seq dataset, we applied the Bayesian model cell2location^96^. In brief, cell2location first estimates reference cell-type signatures from the dissociated scRNA-seq data using negative binomial regression. It then decomposes the spatially resolved Visium RNA count matrices into the reference cell-type signatures.

#### Annotation of anatomical and histological features

We used the microscopy H&E images to annotate anatomical structures and histological features independently of gene expression. Using the package TissueTag^33^, anatomical features were manually labelled, whereas histological features were inferred using a random forest classifier trained on a few manually labelled points. Estimated cell-type abundances per spot in each anatomical structure (or histological feature) were then averaged, and an enrichment score of cell type per anatomical structure (or histological feature) was computed.

#### Scoring of the fetal fallopian tube decreasing signature in adult data

To evaluate whether the rostrocaudal decreasing pattern of expression of the 15 genes identified in the fetal fallopian tube was preserved into adulthood, we generated 10x Visium spatial transcriptomics data from three areas of an adult fallopian tube (fimbriae, ampulla and isthmus) and scored the epithelial spots in each capture area for the average expression of this gene signature. In brief, we used the scanpy^97^ function sc.tl.score_genes() to compute the score and then performed the Jonckheere’s trend test^98^ with the alternative hypothesis ‘decreasing’ (2000 permutations) to quantify the significance of the trend. The same approach was used to test the rostrocaudal increasing pattern of expression.

### Analysis of ISS data

#### Probe selection

To locate cell types and states as they appear and disappear during development of the reproductive tract (especially during early stages of development, which cannot be assayed with spot-based spatial transcriptomics), we designed a panel of 171 genes. The most unique marker genes per cell type were selected using term frequency–inverse document frequency, and the resulting panel was evaluated for completeness using geneBasis^99^. To evaluate completeness at the cell level, geneBasis checks for preservation of a cell’s neighbourhood in the kNN graph built with all the highly variable genes and the kNN graph constructed with the gene panel by comparing the distance between each cell and these two sets of nearest neighbours. geneBasis was applied to evaluate the same gene panel on the three views of the scRNA-seq data (≤10 PCW male and female embryos, >10 PCW male fetuses and >10 PCW female fetuses) separately. However, as thoracic HOX genes are not cited in the literature as relevant to the differentiation of the reproductive tract, these were not included in the original gene panel. We therefore decided to swap four of the genes in the original panels (*DPP4*, *DPP6*, *CRISP3* and *DPP10*) for four HOX genes (*HOXC4*, *HOXC6*, *HOXA7* and *HOXC10*) and ran ISS on some samples with this resulting panel instead (Supplementary Table 3).

#### Preprocessing

ISS data were preprocessed with a computational pipeline implemented by T.L. in Nextflow. In brief, the first step of the pipeline involved integrating the tiles along the *z* axis using maximal projection for each channel and then stitching them together along the remaining *x* and *y* spatial axes. Because the tissue moves slightly between sequencing rounds, image registration was required to correct for spatial misalignment of the fluorescent spots. This registration was achieved using nonlinear optical flow to align the small, Gaussian-like spots in the images. Once the images were stitched and corrected for misalignment, we used the DAPI signal to segment nuclei with CellPose^100^. For computational efficiency, images were first sliced into smaller 10,000 × 10,000 pixels patches. Even though there was no membrane protein staining, cell segmentation could be obtained by expanding the segmented nuclei by about 10 or 15 pixels to mimic the cytoplasm. Finally, spots appearing across registered coding channels were detected and their intensities were extracted. The intensity values for each spot over imaging cycles were then decoded on the basis of the collection of barcodes in the codebook provided by CARTANA with the PoSTcode algorithm^101^. To minimize false positives, PoSTcode inflates the codebook with an additional background barcode. The stacks of image values, representing the intensities across different channels and imaging cycles, were modelled using a matrix-variate Gaussian mixture model, assuming correlations between channels and imaging cycles. Decoded spots were ultimately assigned to segmented cells, which resulted in a gene-expression matrix used for downstream analyses.

#### Cell-type annotation of ISS data based on matched scRNA-seq data

Although the ISS gene panel was designed to maximize cell-type recovery, the throughput of the assay was still too limited to confidently assign cell-type identities solely on the basis of examining the measured gene expression. We therefore leveraged the full transcriptome resolution of the scRNA-seq dataset to increase the confidence in cell-type assignment using an approach based on kNN graphs implemented in the iss-patcher library^102^. Using this approach, we annotated the cells of ISS samples in a per-view fashion (for example, ISS samples for ≤10 PCW were annotated using the ≤10 PCW scRNA-seq reference). In early (≤10 PCW) samples, ISS cells for which the anatomical annotation was ‘gonad’ were excluded from the label-transfer algorithm because there are no gonad cells in the scRNA-seq reference data (by design).

#### Annotation of anatomical structures

The experimental workflow of ISS currently does not involve the acquisition of a H&E image of the sample. We overcame the limitation of not having a H&E image by generating a ‘virtual’ RGB image from the gene-expression profiles of three highly expressed markers (one per R, G and B channel). Major anatomical landmarks were therefore annotated on the virtual RGB image based on the morphology and the H&E image of the consecutive section. Histological landmarks were not annotated, as they necessitate the texture information captured by H&E staining only. By combining the information about the cell-type label of each ISS cell and its annotated anatomical location in the tissue architecture, we then computed an enrichment score (*z* score) of each cell type in each anatomical location (separately per view of the data). Such *z* score enrichment was computed using only the ISS cells with a cell-type label fraction above 0.8 (meaning that 80% of the scRNA-seq neighbours are annotated with the same cell-type label). The per-view enrichment scores were visualized using a heatmap.

#### Computational representation of the Müllerian and Wolffian rostrocaudal axes

Detailed information about the rationale and implementation for the computational representation of the Müllerian and Wolffian rostrocaudal axes can be found in Supplementary Notes 2, 3 and 4; here, we provide a summary.

To study the spatial gene-expression patterns along the developing female reproductive tract, we constructed the Müllerian rostrocaudal axis by measuring distances from key anatomical landmarks in spatially resolved transcriptomics data (10x Visium Cytassist and ISS), as described in the OrganAxis framework^33^. The axis spans from the fallopian fimbriae to the Müllerian vagina–vaginal plate junction, thereby capturing the differentiation of the Müllerian ducts. In cases when the full length of the uterovaginal canal could not be captured in a single section owing to the limitations of the 10x Visium Cytassist platform’s capture area, consecutive tissue sections were computationally stitched together^103^. This stitching involved manually overlapping consecutive sections using the image processing software Fiji (https://imagej.net/plugins/trakem2/) and applying affine transformations to align the sections, which were then concatenated into a single dataset. The resulting Müllerian rostrocaudal axis was normalized and rescaled, which enabled consistent comparison of gene-expression patterns across different samples of the female reproductive tract.

To extend our spatial analysis to single-cell resolution, we projected the Müllerian rostrocaudal axis onto scRNA-seq data by leveraging the single-cell resolution provided by ISS technology. We restricted our analysis to mesenchymal and epithelial compartments, which are key to understanding the development of the female reproductive tract. Using a modified kNN approach implemented in the iss-patcher^102^ library, each cell in the scRNA-seq dataset was assigned a position along the Müllerian rostrocaudal axis based on its proximity to cells in the ISS data. We then used the TradeSeq^88^ framework to model gene expression along the measured (10x Visium Cytassist) and imputed (scRNA-seq) Müllerian rostrocaudal axis, treating the axis analogously to pseudotime. Genes that showed significant changes in expression along the axis were identified using stringent criteria (*P* < 0.001, log[FC] > 0.5), and we prioritized those with specific expression in mesenchymal or epithelial cells.

In parallel, to investigate gene-expression patterns along the male reproductive tract, we constructed the Wolffian rostrocaudal axis, spanning the length of the epididymis and the initial segment of the vas deferens. This axis was derived using data exclusively from 10x Visium Cytassist, as we lacked sufficient ISS male samples for robust imputation of the axis onto scRNA-seq. The axis was similarly defined by calculating the normalized distance between the efferent ductules and the vas deferens. It was then rescaled to maintain consistency with the Müllerian rostrocaudal axis to facilitate comparative analyses. We used the TradeSeq^88^ framework to model gene expression continuously along the Wolffian rostrocaudal axis and used the same significance thresholds as for the Müllerian rostrocaudal axis to prioritize genes for which expression changes along the differentiating Wolffian ducts.

Prioritized spatially variable mesenchymal and epithelial genes in the imputed Müllerian rostrocaudal axis and measured Wolffian rostrocaudal axis were then used to filter the transcription factors and cell–cell communication events that probably drive Müllerian and Wolffian regionalization during fetal development.

### Reporting summary

Further information on research design is available in the Nature Portfolio Reporting Summary linked to this article.

## Online content

Any methods, additional references, Nature Portfolio reporting summaries, source data, extended data, supplementary information, acknowledgements, peer review information; details of author contributions and competing interests; and statements of data and code availability are available at 10.1038/s41586-025-09875-2.

## Supplementary information

Supplementary Information Supplementary Note Figures, Supplementary Table legends and Supplementary Notes 1–5: 1, cell-type annotations; 2, Müllerian rostrocaudal axis; 3: Wolffian rostrocaudal axis; 4, intra-organ regionalization of fallopian tube and epididymis; and 5, extended discussion.

Reporting Summary

Supplementary Fig. 1 Cell-type characterization of early (≤10 PCW) female and male reproductive tract samples.

Supplementary Fig. 2 Cell-type characterization of late (>10 PCW) female reproductive tract samples.

Supplementary Fig. 3 Cell-type characterization of late (>10 PCW) male reproductive tract samples.

Supplementary Tables Supplementary Tables 1–10.

## Source data

Source Data Fig. 4

Source Data Fig. 5

Source Data Extended Data Fig. 8

Source Data Extended Data Fig. 10

## Data Availability

All raw and processed sequencing and imaging data generated in this study have been deposited in public repositories. Sequencing data are available at ArrayExpress under the following accessions: E-MTAB-15475 for scRNA-seq; E-MTAB-15457 for scRNA-seq from organoids; E-MTAB-15479 for scATAC–seq; and E-MTAB-15471 for 10x Visium spatial transcriptomics. Imaging data, including ISS, RNAscope, immunofluorescence and H&E, are available through the BioImage Archive (accession S-BIAD2224). All datasets are publicly accessible. scRNA-seq data used to generate the figures in this paper can also be accessed and downloaded via our web portal: www.reproductivecellatlas.org. Publicly available datasets used in this study were downloaded from ref. ^23^ (Gene Expression Omnibus identifier GSE174712). Source data are provided with this paper.

## Code availability

The code used to perform the analyses presented in the paper can be found at GitHub (https://github.com/ventolab/Human-ReproductiveTract-Development-Atlas).
