# Supplementary material for: Spatiotemporal cellular map of the developing human reproductive tract
Source: Nature. 2025 Dec 17;650(8101):428–37. doi: 10.1038/s41586-025-09875-2 (PMC12893920; doi:10.1038/s41586-025-09875-2)
Supplement: Supplementary file 1 — Supplementary Note Figures, Supplementary Table legends and Supplementary Notes 1–5: 1, cell-type annotations; 2, Müllerian rostrocaudal axis; 3: Wolffian rostrocaudal axis; 4, intra-organ regionalization of fallopian tube and epididymis; and 5, extended discussion. [file 41586_2025_9875_MOESM1_ESM.docx]

SI

[**Supplementary Figure legends 1**](#_9w9f4ijbw2we)

[**Supplementary Table legends 2**](#_6059nzi0g1sm)

[**Supplementary Note 1: Cell type annotations 4**](#_xos5ibx8hgmz)

[1.1 Literature based preliminary annotations 4](#_69eouzib2m60)

[1.2 Spatial mapping to refine annotations 8](#_o9n55oq315bj)

[1.3 Characterisation of the Müllerian ducts and their derivatives 9](#_k9hp7rgu2vtc)

[1.4 Characterisation of Wolffian ducts and their derivatives 13](#_buf21xplkkse)

[1.5 Characterisation of urogenital sinus derivatives 14](#_23s24fuit3xv)

[1.6 Characterisation of genital tubercle derivatives 15](#_fbbcdbjcy9v7)

[**Supplementary Note 2: Müllerian rostro-caudal axis 17**](#_xcv4rcx1oaiy)

[2.1 Deriving the axis from spatially-resolved transcriptomics data 17](#_a0qy3my4lpuc)

[2.2 Projecting the axis onto scRNA-seq data 19](#_nv3epmy9qa5w)

[2.3 Prioritising spatially-variable genes along the axis 20](#_ywab9n2lwabo)

[2.3.1 Müllerian-derived mesenchyme 20](#_t7x29j24f4mu)

[2.3.2 Müllerian-derived epithelium 22](#_bhphmnkyxvvz)

[2.4 Prioritising spatially-variable mesenchymal-epithelial interactions along the axis 22](#_2k3phsee1qw)

[**Supplementary Note 3: Wolffian rostro-caudal axis 23**](#_vy9poetkyduq)

[**Supplementary Note 4: Intra-organ regionalisation of fallopian tube and epididymis 24**](#_sb2wd096hwt9)

[**Supplementary Note 5: Discussion 25**](#_6rpbnm1i2wa3)

[**Supplementary References 28**](#_5lyabz2eemeo)

#

#

# Supplementary Figure legends

**Supplementary Fig. 1. Cell type characterisation of early (<= 10 PCW) female and male reproductive tract samples.** **a,** Batch-corrected Uniform Manifold Approximation and Projection (UMAP) embedding of female and male samples <= 10 PCW profiled with scRNA-seq (n = 185,373 cells) coloured by stage (measured in PCW), sex, donor, and cell type. **b,** Dot plot showing the variance-scaled, log-transformed expression of genes (x-axis) characteristic of the annotated cell types (y-axis) detected in female and male samples <= 10 PCW. Top-layer groups marker genes by developing organs. **c,** Dot plots showing the variance-scaled, log-transformed expression of genes (x-axis) characteristic of the annotated cell types (y-axis) in male and female samples <= 10 PCW as measured by means of (left) *In Situ* Sequencing (*ISS*) and (right) scRNA-seq to evaluate the specificity of our *ISS* gene panel.

**Supplementary Fig. 2. Cell type characterisation of late (> 10 PCW) female reproductive tract samples.** **a,** Batch corrected Uniform Manifold Approximation and Projection (UMAP) embedding of female samples > 10 PCW profiled with scRNA-seq (n = 227,932 cells) coloured by stage (measured in PCW), donor and cell type. **b,** Dot plot showing the variance-scaled, log-transformed expression of genes (x-axis) characteristic of the annotated cell types (y-axis) detected in female samples > 10 PCW. Top-layer groups marker genes by developing organs. **c,** Dot plots showing the variance-scaled, log-transformed expression of genes (x-axis) characteristic of the annotated cell types (y-axis) in female samples > 10 PCW as measured by means of (left) *In Situ* Sequencing (*ISS*) and (right) scRNA-seq to evaluate the specificity of our *ISS* gene panel.

**Supplementary Fig. 3. Cell type characterisation of late (> 10 PCW) male reproductive tract samples.** **a,** Batch corrected Uniform Manifold Approximation and Projection (UMAP) embedding of male samples > 10 PCW profiled with scRNA-seq (n = 125,437 cells) coloured by stage (measured in PCW), donor and cell type. **b,** Dot plot showing the variance-scaled, log-transformed expression of genes (x-axis) characteristic of the annotated cell types (y-axis) detected in male samples >10 PCW. Top-layer groups marker genes by developing organs. **c,** Dot plots showing the variance-scaled, log-transformed expression of genes (x-axis) characteristic of the annotated cell types (y-axis) in male samples > 10 PCW as measured by means of (left) *In Situ* Sequencing (*ISS*) and (right) scRNA-seq to evaluate the specificity of our *ISS* gene panel.

# Supplementary Table legends

**Supplementary Table 1**. Metadata of samples. **(A)** 10x scRNA-seq libraries. **(B)** *10x* scATAC-seq and *10x* cell-coupled snRNA/ATAC-seq multiomic libraries. **(C)** *10x Visium* spatially-resolved transcriptomics libraries. **(D)** *In Situ* Sequencing (*ISS*) spatially-resolved transcriptomics libraries. Columns across tables indicate: Sample ID = 10x reaction; Donor ID = donor ID; Sex = female or male determined by karyotype and sequencing; Developmental stage (PCW) = post-conceptional weeks; Developmental stage (CS) = Carnegie Stage, if appropriate; Location = spatial location within the reproductive tract; Notes = additional information about each library, where appropriate. Columns specific to scRNA-seq libraries indicate: Multiplexed = if more than one donor were multiplexed. Columns specific to scATAC-seq libraries indicate: Multiome = if the 10x cell-coupled snRNA/ATACseq multiomic kit was used. Columns specific to *10x Visium* libraries indicated: Technology = *Visium* or *Visium Cytassist*; Slide = slide number; Position = capture area on the slide. Columns specific to *ISS* libraries include: Gene panel = original or HOX replacement panel.

**Supplementary Table 2.** Quality control of samples. **(A)** Summary statistics from STARSolo for each *10x* scRNA-seq library in our fetal reproductive tract atlas. **(B)** Summary statistics from *10x* Cell Ranger ATAC 2.0.0 (scATAC-seq) and *10x* Cell Ranger ARC 2.0.0 (multiomics) for each *10x* ATAC library in our fetal reproductive tract atlas. **(C)** Summary statistics from *10x* SpaceRanger 2.1.0 for each 10x Visium and *Visium Cytassist* library.

**Supplementary Table 3.** *ISS* gene panel and RNAscope probes. **(A)** Gene name and ENSEMBL ID for the genes selected in our ISS panel (both original and HOX replacement). **(B)** Information about the probes used for smFISH imaging experiments.

**Supplementary Table 4**. Marker genes per cell type. **(A)** Marker genes computed with TF-IDF for each cell type annotated in <= 10 PCW female and male scRNA-seq samples. **(B)** Marker genes computed with TF-IDF for each cell type annotated in >10 PCW female scRNA-seq samples. **(C)** Marker genes computed with TF-IDF for each cell type annotated in >10 PCW male scRNA-seq samples.

**Supplementary Table 5**. Temporally-variable genes during Müllerian duct formation. Output of *TradeSeq*’s *associationTest()* that tests whether the coefficients of the smoothed splines for each gene are significantly different from each other (i.e. the gene trend is associated with pseudotime).

**Supplementary Table 6.** *CellPhoneDB* interactions (Wolffian-to-Müllerian duct signalling during Müllerian formation). *CellPhoneDB* results for the cell-cell communication analysis between Wolffian and Müllerian duct during Müllerian duct formation (6-8 PCW). Interactions are filtered to include only those where all the members of the interaction are expressed in at least 10% cells and at least one member is a DEGs between cell types with a log2-fold change above 0.5 and adjusted p-value < 0.001.

**Supplementary Table 7**. Spatially-variable genes along the Müllerian and Wolffian rostro-caudal axes. **(A)** Output of *TradeSeq*’s *associationTest()* that tests whether the coefficients of the smoothed splines for each gene are significantly different from each other (i.e. the gene trend is associated with the axis) in Müllerian-derived mesenchymal *10x Visium Cytassist* spots. **(B)** Output of *TradeSeq*’s *associationTest()* in Müllerian-derived mesenchymal scRNA-seq cells. **(C)** Output of *TradeSeq*’s *associationTest()* in Müllerian- derived epithelial *10x Visium Cytassist* spots. **(D)** Output of *TradeSeq*’s *associationTest()* in Müllerian-derived epithelial scRNA-seq cells. **(E)** Output of *TradeSeq*’s associationTest() in Wolffian-derived mesenchymal *10x Visium Cytassist* spots.

**Supplementary Table 8**. Spatially-variable genes along the fallopian tube and epididymal rostro-caudal axes. **(A)** Output of *TradeSeq*’s *associationTest()* that tests whether the coefficients of the smoothed splines for each gene are significantly different from each other (i.e. the gene trend is associated with the axis) in fallopian tube epithelial *10x Visium Cytassist* spots. **(B)** Output of *TradeSeq*’s *associationTest()* in epididymis epithelial *10x Visium Cytassist* spots.

**Supplementary Table 9**. *CellPhoneDB* interactions (*early* *corpus spongiosum*-to-urethral epithelium signalling in the external genitalia). *CellPhoneDB* results for the cell-cell communication analysis between male *early* *corpus spongiosum* and urethral epithelium during the masculinisation programming window (8-14 PCW). Interactions are filtered to include only those where all the members of the interaction are expressed in at least 10% cells and at least one member is a DEGs between males and females with a log2-fold change above 0.5 and adjusted p-value < 0.001.

**Supplementary Table 10**. Clinically approved drugs identified by *drug2cell* as potentially affecting developing reproductive tract epithelia. **(A)** Results of *drug2cell* analysis for <=10 PCW female and male scRNA-seq samples. **(B)** Results of *drug2cell* analysis for >10 PCW female scRNA-seq samples. **(C)** Results of *drug2cell* analysis for >10 PCW male scRNA-seq samples. Across all tables, results were filtered based on adjusted p-value (< 0.01), log-fold changes (> 2), and rank scores to select the most significant drugs associated with the target cell type. An additional filtering step was performed to exclude drugs whose target genes are not specific to the target cell type and require that the targets are expressed in at least 10% of cells in the target cell type.

#

# Supplementary Note 1: Cell type annotations

## 1.1 Literature based preliminary annotations

As the majority of our knowledge of the human reproductive ducts comes from studies using hematoxylin and eosin (H&E) stained sections[^1^](https://paperpile.com/c/zlrttx/TFbHY), and murine markers are not necessarily useful (as we have previously demonstrated in the developing gonads[^2^](https://paperpile.com/c/zlrttx/bDFDH)), we needed to perform cell type annotation de novo. Each scRNA-seq library was initially analysed separately before integration to properly assess the quality of the integration. To find the genes characteristic of each cluster, we performed term frequency - inverse document frequency (*TF-IDF*). This method, implemented in the R library *SoupX*[*^3^*](https://paperpile.com/c/zlrttx/8SeZi), indicates the significance of a word (gene) to a document (cluster) within a corpus (dataset). Clusters were named based on known cell types from the literature if the marker genes matched. If not, we used precise spatial mapping of clusters onto tissue architecture using both *10x Visium* and *In Situ* Sequencing (*ISS*). The table below (**Supplementary Note Table 1**) summarises the marker genes per cell type in the developing reproductive tract, compiled from the literature, which were used to guide the annotations.

| **Cell type** | **Marker** | **Literature sources** |
| --- | --- | --- |
| Urogenital system (general TFs) | *EMX2* | [^4^](https://paperpile.com/c/zlrttx/uF2II) |
|  | *WT1* | [^5,6^](https://paperpile.com/c/zlrttx/bZLD5+4wQnP) |
|  | *GATA2* | [^7,8^](https://paperpile.com/c/zlrttx/5PnDw+MyKp2) |
|  | *LHX1* | [^9,10^](https://paperpile.com/c/zlrttx/Fv6Ey+h9iK3) |
| Coelomic epithelium (mesothelium) | *UPK3B* | [^11^](https://paperpile.com/c/zlrttx/PU5G1) |
|  | *LRRN4* | [^12^](https://paperpile.com/c/zlrttx/uUlOC) |
| Müllerian duct epithelium | *WNT7A* | [^13,14^](https://paperpile.com/c/zlrttx/5bqpE+5Cq8U) |
|  | *SOX17* | [^15,16^](https://paperpile.com/c/zlrttx/bfgQw+qrokP) |
| Wolffian duct epithelium | *WNT9B* | [^17^](https://paperpile.com/c/zlrttx/pDMfB) |
|  | *GATA3* | [^18^](https://paperpile.com/c/zlrttx/bwk6x) |
| Müllerian / Wolffian duct mesenchyme | *WNT4* | [^19^](https://paperpile.com/c/zlrttx/LUZLr) |
|  | *WNT5A* | [^20^](https://paperpile.com/c/zlrttx/NgAx2) |
| Müllerian duct mesenchyme | *AMHR2* | [^21^](https://paperpile.com/c/zlrttx/k2eI) |
| Urogenital sinus epithelium | *SHH* | [^22^](https://paperpile.com/c/zlrttx/lI8mq) |
|  | *FOXA1* | [^23–25^](https://paperpile.com/c/zlrttx/Fl9wO+nlRm7+XP1OO) |
| Urogenital sinus mesenchyme | *TNC* | [^26^](https://paperpile.com/c/zlrttx/ZmsU) |
|  | *FOXF1* | [^27^](https://paperpile.com/c/zlrttx/K7ww) |
| Urethral epithelium | *PSCA* | [^28^](https://paperpile.com/c/zlrttx/oI6i) |
|  | *UPK1A* | [^29^](https://paperpile.com/c/zlrttx/VIlV) |
| Genital epidermis | *KRT14* | [^30^](https://paperpile.com/c/zlrttx/hb7y6) |
| Glans | *DLX5* | [^31,32^](https://paperpile.com/c/zlrttx/cdYvC+9mj4k) |
|  | *MSX1* | [^32^](https://paperpile.com/c/zlrttx/9mj4k) |
| Corpus cavernosum | *SOX9* | [^31,33,34^](https://paperpile.com/c/zlrttx/cdYvC+zgIgN+3kDn6) |
|  | *ZFHX4* | [^32^](https://paperpile.com/c/zlrttx/9mj4k) |
| Corpus spongiosum | *FOXF1* | [^32,35^](https://paperpile.com/c/zlrttx/GKBgq+9mj4k) |
|  | *TCF21* | [^32,35^](https://paperpile.com/c/zlrttx/GKBgq+9mj4k) |
| Prepuce | *SHOX2* | [^36^](https://paperpile.com/c/zlrttx/Jgif) |
| Uterus epithelium | *DLX5* | [^37^](https://paperpile.com/c/zlrttx/lab3V) |
|  | *MSX1* | [^38^](https://paperpile.com/c/zlrttx/rzvUY) |
| Müllerian vagina epithelium | *TP63* | [^39^](https://paperpile.com/c/zlrttx/DeyrF) |
|  | *PAX2* | [^1^](https://paperpile.com/c/zlrttx/TFbHY) |
| Vaginal plate epithelium | *FOXA1* | [^1,40^](https://paperpile.com/c/zlrttx/u3urK+TFbHY) |
|  | *TP63* | [^39^](https://paperpile.com/c/zlrttx/DeyrF) |
| Epididymal epithelium | *SPAG11B* | [^41^](https://paperpile.com/c/zlrttx/ulMrl) |
| Vas deferens epithelium | *WNT9B* | [^42^](https://paperpile.com/c/zlrttx/yd6V) |
| Prostate epithelium | *FOXA1* | [^43^](https://paperpile.com/c/zlrttx/pC7w) |
| Kidney - distal tubule | *TMEM52B* | [^44,45^](https://paperpile.com/c/zlrttx/8zr0G+lN9sp) |
| Kidney - proximal tubule | *GLYAT* | [^46^](https://paperpile.com/c/zlrttx/I2wz) |
| Kidney - loop of Henle | *SLC12A1* | [^47^](https://paperpile.com/c/zlrttx/I3Xci) |
| Kidney - podocyte | *NPHS1* | [^48,49^](https://paperpile.com/c/zlrttx/Jt7mK+86Zun) |
|  | *PTPRO* | [^50^](https://paperpile.com/c/zlrttx/McIEp) |
| Adrenal gland cortex | *SHISA3* | [^51^](https://paperpile.com/c/zlrttx/gryk0) |
|  | *RFXP2* | [^52^](https://paperpile.com/c/zlrttx/yGWcD) |

**Supplementary Note Table 1.** Table reporting major cell type markers taken from the literature alongside the relevant publications. Green highlights cell types found in the sexually undifferentiated stages of reproductive development, while red and blue indicate cell types present in the differentiated female and male reproductive tracts, respectively. Yellow instead showcases cell types which are not part of the developing reproductive system but due to spatial proximity are present in our data.

The final cell type markers shown in the dotplots in **Supplementary Figs. 1b, 2b, 3b** were chosen based on their specificity and robustness across modalities. Genes were prioritised if they reliably (i) distinguished cell types in scRNA-seq, (ii) mapped to distinct spatial domains in *ISS* and *10x Visium*, and (iii) showed consistent expression across multiple samples (**Supplementary Note Fig. 1a-b**). This unbiased approach enabled the discovery of novel markers in reproductive tract development while validating known markers against the literature.


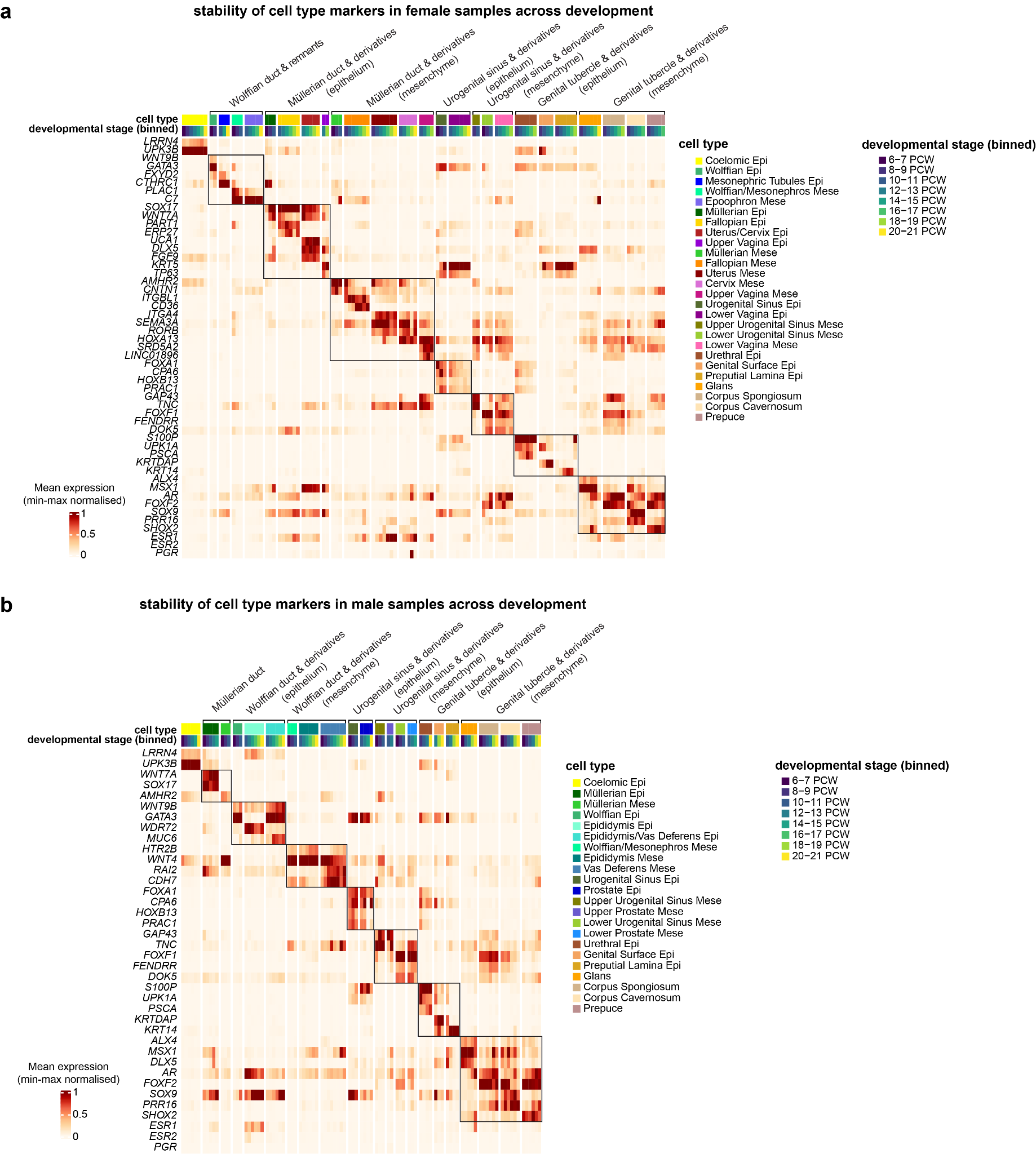


**Supplementary Note Fig. 1. a**, Heatmap showing the expression of the selected marker genes for the epithelial and mesenchymal cell types in the female reproductive tract across all developmental stages (6-21 PCW). **b,** Heatmap showing the expression of the selected marker genes for the epithelial and mesenchymal cell types in the male reproductive tract across all developmental stages (6-21 PCW).

## *1.2 Spatial mapping to refine annotations*

As the developing reproductive system comprises multiple organs and changes quite significantly in size as gestation progresses, each *10x Visium* capture area contains different (combinations of) anatomical structures. When choosing the tissue sections devoted to Visium, the aim was to get representative sections of the whole reproductive system over different developmental timepoints. While optimal for obtaining a comprehensive understanding of the developing reproductive system, this choice of experimental design results in *10x Visium* samples not being immediately comparable. Similarly to *10x Visium*, tissue sections devoted to *ISS* were chosen to capture the morphological diversity of the developing reproductive system over developmental timepoints, with a particular focus on early stages where *10x Visium* cannot be applied due to nuclei being too densely packed and tissue sections being too small. However, by annotating the anatomical/histological structures on each *10x Visium* capture area or *ISS* slide, we were able to pool information from multiple samples that contain the same structures (**Methods**).

For each of the developing female internal genitalia organs, we distinguish mesenchymal cells adjacent to the epithelium from those in the ligament surrounding the organ (**Extended Data Fig. 2d**). A layer of smooth muscle separates the inner mesenchymal cells in contact with the epithelium from the outer mesenchymal cells in the ligament, but the smooth muscle layer is absent in the rostral half of the fallopian tube until 21 PCW (**Extended Data Fig. 2h**, **Supplementary Note Fig. 2a**). This observation is consistent with recent evidence from the adult fallopian tube showing that the isthmus has the greatest percentage of smooth muscle cells[^53^](https://paperpile.com/c/zlrttx/VHm4). Unlike their female counterparts, the male internal genitalia exhibit less histological compartmentalisation; mesenchymal and smooth muscle markers are co-expressed by the same myo-mesenchymal cells directly in contact with the epithelial layer (**Supplementary Note Fig. 2b**).


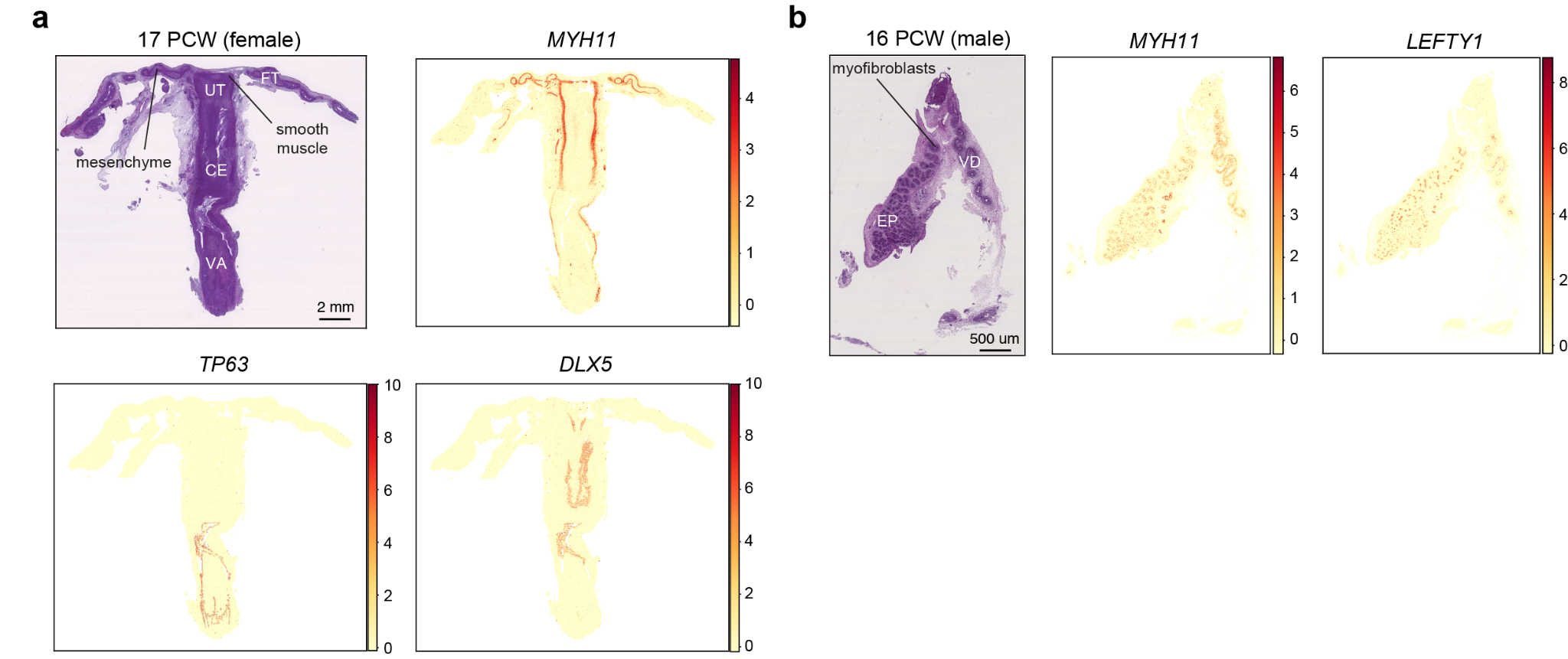


**Supplementary Note Fig. 2. a,** Hematoxylin and eosin (H&E) stained image of a representative section of a 17 PCW female sample profiled with *In Situ* Sequencing (*ISS*) alongside the measured expression of *MYH11* (smooth muscle marker), *TP63* (vaginal stratified epithelium marker), *DLX5* (uterine epithelium marker) (n = 4 biologically independent samples). Scale bar: 2 mm. **b,** H&E stained image of a representative section of a 16 PCW male samples profiled with *ISS* alongside the measured expression of *MYH11* (smooth muscle marker), *LEFTY1* (epididymis and vas deferens epithelium marker) (n = 1 biologically independent sample). Scale bar: 500 μm. CE: cervix; EP: epididymis; FT: fallopian tube; UT: uterus; VA: vagina; VD: vas deferens.

## *1.3 Characterisation of the Müllerian ducts and their derivatives*

By combining literature-derived markers with spatial mapping to both anatomical and histological structures, we were able to characterise with unparalleled resolution the Müllerian epithelial and mesenchymal cells from their emergence in female and male embryos (6-8 PCW) to their female-specific differentiation into the fallopian tube, uterus, cervix, and upper vagina (~9/10 until 21 PCW). Here, we summarise the markers and the spatial coordinates of these cells in these two developmental time windows:

● Müllerian duct (6-8 PCW): both epithelial and mesenchymal progenitors of the Müllerian ducts are specified in the extra-gonadal coelomic epithelium, which is characterised by the expression of *UPK3B*, *LRRN4*, and *GATA2*, and upregulates the transcription factor (TF) *EMX2* (**Supplementary Fig. 1b**).

○ Müllerian epithelial cells emerge from the coelomic epithelium as they upregulate *RXRG*, *PNOC*, and *LYPD1* (**Fig. 2c**, **Supplementary Fig. 1b**). Later along the reconstructed trajectory, they exhibit increased expression of genes characteristic of neuronal and axonal migration (*FGF20*, *SSTR2*, *GDNF*, *CALCA*) (**Fig. 2c**). A bulk RNA-seq study in chicken embryos had already noted the surprising upregulation of neuronal markers during Müllerian elongation but were unable to pinpoint the source of the signal due to the nature of the assay[^54^](https://paperpile.com/c/zlrttx/NnzLm). Using single-molecule fluorescence in situ hybridisation (smFISH) we further confirmed the specificity of *PNOC*, *LYPD1*, *FGF20*, *GDNF*, and *CALCA* to the Müllerian epithelium during the sexually undifferentiated stages of development (**Fig. 2d, Extended Data Fig. 4b**). Notably, while *FGF20* is expressed throughout the length of the Müllerian epithelium, the expression of *PNOC* and *LYPD1* is confined to the rostral region while the expression of *GDNF* and *CALCA* is restricted to the caudal portion. This finding suggests that there might be a spatial, in addition to temporal, aspect to the expression of these newly identified regulators of Müllerian emergence. Given that Müllerian duct formation initiates rostrally and elongates caudally, gene expression patterns along pseudotime may reflect both temporal dynamics and positional identity. During both phases of specification and caudal elongation of the Müllerian duct, the epithelium is also marked by the expression of TFs (*PAX8*, *PAX2*, *LHX1*, *RUNX1*, *RUNX2*, *SOX17*) and signalling molecules (*WNT7A*) that have been extensively described in the literature (**Extended Data Fig. 4h**, **Supplementary Note Table 1**).

○ Müllerian mesenchymal cells arise from the coelomic epithelium by instead upregulating epithelial-to-mesenchymal transition (*CNTN1*, *ZEB2*, *SNAI2*) and migratory (*TMEM163*, *PLXDC1, ZAP70*) genes (**Fig. 2c**). While no specific markers of the human Müllerian mesenchyme had been previously reported, by employing *ISS* and smFISH we were able to validate the specificity of markers like *TMEM163* to this cell type (**Extended Data Fig. 4c-d**).

● Müllerian duct-derived epithelial cells (~9-21 PCW) become evident in female fetuses around 9 PCW, as they downregulate migratory genes (*FGF20*, *SSTR2*, *GDNF*, *CALCA*) and start expressing organ-specific markers (**Supplementary Fig. 2a** and also summarised for convenience in **Supplementary Note Fig. 3a**).

○ Fallopian tube epithelial cells maintain high expression of *WNT7A* and *SOX17* and specifically upregulate *ERP27*, *DAPL1*, and *PART1*. Moreover, they show upregulation of *PNOC* and *LYPD1*, which were previously identified as markers of the early/rostral Müllerian epithelium (**Fig. 2d**). When investigating intra-organ heterogeneity in the fallopian tube epithelium, we were able to further pinpoint the expression of these two genes to the rostral-most and mid portion of the fallopian tube, respectively (**Fig. 3j-k**, **Extended Data Fig. 7j**). Interestingly, the adult fallopian tube continues to express *PNOC* and *LYPD1* preferentially in the fimbriae and ampulla (**Fig. 3l-n**). Some fallopian tube epithelial cells also acquire markers of multi-cilia (*DNAH12*, *DNAI1*), which we do not observe in other organs of the female reproductive tract until 21 PCW (**Supplementary Fig. 4a-b**).

○ Uterocervix epithelial cells keep expressing *WNT7A* and *SOX17* and concurrently upregulate *LGR5*, *DLX5*, *UCA1*, *MSX1*, and *MSX2* . Until the latest stage present in our dataset (21 PCW) we do not report any transcriptomic differences between epithelial cells residing in the developing uterus vs cervix (**Extended Data Fig. 2c**), suggesting that further regionalisation happens later in development and might be regulated by hormones. Notably, when comparing the transcriptomic profile of fetal uterocervix epithelial cells to adult endometrial cells found in the proliferative phase of the menstrual cycle[^55^](https://paperpile.com/c/zlrttx/zgll), we noted that fetal cells express a combination of luminal (*LGR5*, *WNT7A*) and basal (*CDH2*, *AXIN2*, *ALDH1A1*) adult endometrial epithelial cells (**Supplementary Note Fig. 3a**).

○ Upper/Müllerian-derived vagina epithelial cells resemble uterocervix cells in their expression of *WNT7A, SOX17, LGR5, DLX5, UCA1, MSX1*, and *MSX2* but are further characterised upregulation of the master regulator of epithelial stratification *TP63* and *KRT5*. Histologically, they still appear as a simple columnar epithelium, which contrasts the lower vaginal epithelium that present instead as a solid “plate” of stratified epithelium (**Extended Data Fig. 2g**).

● Müllerian-derived mesenchymal cells (~9-21 PCW) also show downregulation of migratory genes (*TMEM163*, *PLXDC1, ZAP70*) accompanied by increased expression of organ-specific markers (**Supplementary Fig. 2a-b**, and also summarised for convenience in **Supplementary Note Fig. 3b**). It is worth noticing that the mesenchymal cells described here are those that are found adjacent to the epithelium and therefore are directly derived by differentiation of the Müllerian mesenchyme (**Extended Data Fig. 2d**).

○ Fallopian tube mesenchymal cells exhibit increased expression of *ITGBL1, CD36, LGR5*, and *TSPAN8*. While *LGR5* is canonically considered an epithelial marker, by using smFISH we were able to clearly show how the expression of *LGR5* changes from mesenchymal to epithelial moving caudally from the fallopian tube to the uterovaginal canal (**Extended Data Fig. 7f**). Moreover, the co-expression of *LGR5* and *TSPAN8* in the fallopian mesenchyme is reminiscent of features of a stem cell niche that have been described in other organs[^56^](https://paperpile.com/c/zlrttx/x2cK) (**Extended Data Fig. 7e**). By modelling gene expression continuously along the Müllerian rostro-caudal axis (details on its derivation in **Supplementary Note 2**), we revealed that the rostral region of the fallopian tube is marked by thoracic *HOX* genes (*HOXA5, HOXC5, HOXC6, HOXA7*) while the caudal portion shows upregulation of *HOXA9*, which had been previously reported as responsible for regionalising the entirety of the fallopian tubes[^57^](https://paperpile.com/c/zlrttx/lb9Z) (**Fig. 3e-g**).

○ Uterine mesenchymal cells are characterised by the expression of *ITGA4*, *RORB*, as well as the lumbosacral *HOX* markers *HOXA10/11* (**Fig. 3e-f**, **Supplementary Fig. 2a-b**).

○ Cervical mesenchymal cells can be distinguished from mesenchymal cells in the uterus by the additional expression of *HOXA13* (**Fig. 3e-f**, **Supplementary Fig. 2a-b**). Fallopian, uterine and cervical mesenchymal cells all share the expression of *EMX2*, *FOXL2*, and *LEPR2*.

○ Upper vagina mesenchymal cells downregulate *EMX2*, *FOXL2*, and *LEPR2*, and instead exhibit increased expression of *AR* and *SRD5A2*, which is the primary enzyme responsible for the conversion of testosterone into its potent derivative dihydrotestosterone (DHT)[^58^](https://paperpile.com/c/zlrttx/YJ2O) (**Supplementary Fig. 2a-b**). While testosterone is not present in the female reproductive system due to the absence of fetal testes, conditions such as congenital adrenal hyperplasia – where there is an abnormally high production of testosterone by the adrenal gland – can result in masculinisation of the lower reproductive tract in XX individuals[^59^](https://paperpile.com/c/zlrttx/4FMs). The expression pattern of these genes raises the question that, unlike the epithelium, which is surely derived from the Müllerian ducts[^1^](https://paperpile.com/c/zlrttx/TFbHY), the mesenchyme of the upper vagina might be derived by the urogenital sinus. However, as lineage tracing information is impossible to obtain in humans, we only document it as an observation.


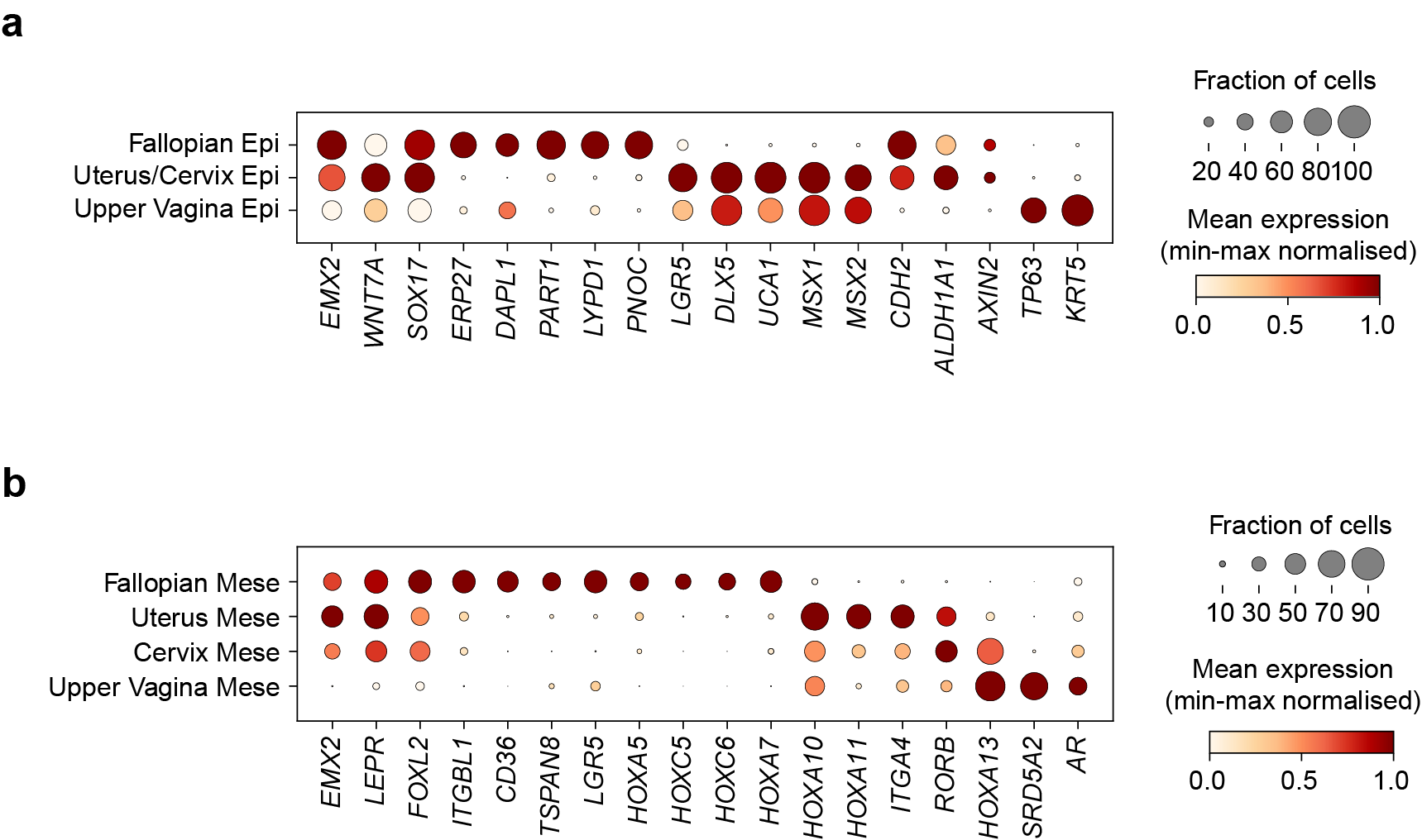


**Supplementary Note Fig. 3**. **a,** Dot plots showing the log-transformed, variance-scaled expression of genes characteristic of Müllerian-derived epithelial cell types identified in our scRNA-seq dataset. **b,** Dot plots showing the log-transformed, variance-scaled expression of genes characteristic of Müllerian-derived epithelial cell types identified in our scRNA-seq dataset.

##

## *1.4 Characterisation of Wolffian ducts and their derivatives*

##

Similarly to the Müllerian ducts, we could also comprehensively characterise the Wolffian epithelial and mesenchymal cells from their undifferentiated stage in female and male embryos (6-8 PCW) to their male-specific differentiation into the epididymis and vas deferens (~9/10 until 21 PCW). Our dataset, however, does not capture the developmental window during which the Wolffian ducts emerge from the intermediate mesoderm (4-6 PCW). Moreover, as the vas deferens gets damaged during dissections, only the epididymis and upper segment of the vas deferens were studied, and therefore we cannot comment on the development of the seminal vesicle. With these limitations in mind, we summarise the markers and spatial coordinates of Wolffian epithelial and mesenchyme cells from 6 to 21 PCW:

● Wolffian epithelial cells (6-8 PCW) are characterised by the expression of well-described TF (*PAX2, PAX8, EMX2, LHX1*) shared with the Müllerian duct as well as the Wolffian-specific TF *GATA3* and signalling molecule *WNT9B* (**Supplementary Fig. 1a-b**).

● Wolffian mesenchymal cells (6-8 PCW) exhibit expression of *PLAC1, ADH1B, ALX1, FOXC2* (**Supplementary Fig. 1a-b**). While no Wolffian-specific markers have been identified previously in the mesenchyme, we were able to validate the cell type specificity of markers like *PLAC1* via *ISS* (**Supplementary Fig. 1c**).

● In Wolffian-derived epithelial cells (~9-21 PCW), the expression of *GATA3* and *WNT9B* then becomes confined to the caudal two-thirds of the epididymis and vas deferens (**Supplementary Fig. 3a-b** and **Extended Data Fig.** **8a-b**), while the efferent ductules acquire a very distinct expression profile characterised by upregulation of *PDZK1, SPAG11A, ESR1, GLYAT,* and *SPINK2* (**Supplementary Fig. 3a-b** and **Extended Data Fig. 11a**)*.* Moreover, consistent with histological reports in adulthood[^60^](https://paperpile.com/c/zlrttx/w8z2P), ciliated epithelial cells were exclusively found in the efferent ductules, while non-ciliated epithelial cells were distributed throughout the fetal epididymis and vas deferens (**Extended Data Fig. 3e**).

● We showed that the Wolffian-derived mesenchymal cells (9-21 PCW) are patterned by *HOX* genes analogously to Müllerian-derived mesenchymal cells, whereby the rostral half of the epididymis upregulates members of the thoracic *HOX* code while the caudal half expresses *HOXA9* (**Extended Data Fig. 5g-j**). The vas deferens mesenchyme is characterised by upregulation of *HOXA10/11* (**Extended Data Fig. 5g-j**). Additionally, the epididymis retains the expression of the early Wolffian duct mesenchymal *PLAC1* and *ADH1B,* while the vas deferens upregulates *CDH7* and *RAI2* (**Supplementary Fig. 3a-b**).

##

## *1.5 Characterisation of urogenital sinus derivatives*

Caudal to the Müllerian and Wolffian ducts, the embryonic urogenital sinus develops in a hormone-independent way as a caudal extension of the hindgut[^61^](https://paperpile.com/c/zlrttx/Pmp5d). The mesenchyme of the urogenital sinus is capable of responding to DHT, leading to the masculinisation of the tissue into the prostate in males. In the absence of DHT, as in XX embryos, the urogenital sinus differentiates into the lower portion of the vagina. Due to damage to the vas deferens during dissections of male samples, it is challenging to identify the prostate as it does not come as attached to the upper reproductive tract and there are not many descriptions of its fetal development in humans that can be used to guide our annotations. Nevertheless, according to the expression of marker genes and the developmental equivalence between the lower vagina and the prostate, we were able to characterise the following cell types:

- Urogenital sinus epithelial cells express *FOXA1*, *SSH*, *TP63*, *AGR2, CPA6* and *GATA3* (**Supplementary Fig. 1a-b**, **Supplementary Table 4**) and can be spatially mapped with very high precision (**Fig. 1d**, **Extended Data Fig. 1h**).
- Urogenital sinus mesenchymal cells surround epithelial cells and are characterised by the expression of *HOXA13* and *ISL1* (**Extended Data Fig. 1h**, **Supplementary Fig. 12a-b**, **Supplementary Table 4**). We further distinguish two sub-populations based on their spatial location within the developing reproductive tract, named upper and lower, which are marked by the expression of *GAP43/TNC/SRD5A2* and *FENDRR/FOXF1*, respectively (**Extended Data Fig. 1h**, **Supplementary Fig. 1a-b**, **Supplementary Table 4**).
- Lower vagina epithelial cells maintain the expression of *FOXA1*, *SSH*, *TP63*, *AGR2, CPA6* and *GATA3,* and further upregulate markers such as *HCAR2* and *HOXB13* (**Extended Data Fig. 2c**, **Supplementary Fig. 2a-b**, **Supplementary Table 4**).
- Lower vagina mesenchymal cells also share expression of *HOXA13* and *ISL1* as well as *FENDRR* and *FOXF1* with the lower urogenital sinus mesenchymal precursors, while also upregulating the BMP ligand *GDF10* (**Extended Data Fig. 2c**, **Supplementary Fig. 2a-b**, **Supplementary Table 4**).
- Prostate epithelial cells, similarly to lower vaginal epithelial cells, maintain the expression of *FOXA1*, *SSH*, *TP63*, *GPX2, CPA6* and *KRT5* (**Supplementary Fig. 3a-b**, **Supplementary Table 4**)
- We distinguish two clusters of prostate mesenchymal cells, which we name upper and lower due to shared expression of marker genes with the upper and lower urogenital sinus mesenchymal cells, respectively (**Supplementary Fig. 3a-b**, **Supplementary Table 4**). It is worth noting that what we are currently annotating as the upper prostate might be the seminal vesicle, which we do not capture in any spatial sample. This hypothesis would be supported by the observation that, in females, the upper vagina (equivalent of the seminal vesicle) expresses the markers of the upper urogenital sinus mesenchyme.

##

## *1.6 Characterisation of genital tubercle derivatives*

The human penis and clitoris arise from the sexually bipotent genital tubercle, which appears around 6 PCW[^62^](https://paperpile.com/c/zlrttx/u0Mrm). During the early, androgen-independent phase of development, the genital tubercle in both male and female human embryos shows comparable size and histological features, as noted by[^30^](https://paperpile.com/c/zlrttx/hb7y6). It comprises structures derived from all three germ layers: the urethral epithelium from the endoderm[^63^](https://paperpile.com/c/zlrttx/XB3j2); the erectile tissues and glans from the mesoderm[^64^](https://paperpile.com/c/zlrttx/PPRnH); and the prepuce along with the genital epidermis from the ectoderm[^65^](https://paperpile.com/c/zlrttx/ZTEnZ). In the later, androgen-dependent phase of development, the action of DHT causes the genital tubercle to develop into the penis, the labioscrotal swellings to fuse forming the scrotum, and the urethral epithelium to undergo canalisation starting from 9.5 PCW[^30^](https://paperpile.com/c/zlrttx/hb7y6). Two recent scRNA-seq studies on the murine genital tubercle[^31,32^](https://paperpile.com/c/zlrttx/cdYvC+9mj4k) (covering both the androgen-independent and androgen-dependent phases of development) did not report any differences at the level of cell type composition between the sexes, suggesting that DHT signalling might exert its action by changing gene expression without resulting in the appearance of new male-specific cell types. Consistent with these findings, our data does not provide evidence for sex-specific cell types in the human genital tubercle/penis/clitoris until 21 PCW, with all organs sharing the following mesenchymal and epithelial cell types:

- Glans (mesenchymal) cells (**Supplementary Figs. 1a-b**, **2a-b**, **3a-b**) express transcription factors *DLX5, MSX1, MSX2*, *ALX4, TFAP2A, SP9* as well as the non-canonical WNT ligand *WNT5A.*
- Corpus cavernosum (mesenchymal) cells (**Supplementary Figs. 1a-b**, **2a-b**, **3a-b**) are characterised by the expression of *ZFHX4, PRR16, PRRX2, SOX9,* and exhibit stage-specific transcriptional changes (**Extended Data Fig. 8a-b**) with expression of *RFLNA* and *GAS2* enriched in early development and expression of *TTHY1* and *SCRG1* enriched in late development.
- Corpus spongiosum (mesenchymal) cells (**Supplementary Figs. 1a-b**, **2a-b**, **3a-b**) express *FOXF1* and *SALL1,* and also exhibit stage-specific transcriptional changes: *GRID2* and *FOXL2* show heightened activity in early stages, while *PDLIM3* and *TCF21* become upregulated in late stages (**Extended Data Fig. 8a-b**). *AR* exhibits the highest expression and cistrome accessibility (as inferred by scATAC-seq) in the corpus spongiosum compared to all other cell types in the differentiating genital tubercle, consistent with its key role in aiding urethral canalisation by moving medially and merging at the penis' midline (**Extended Data Fig. 9d**). Putative effector genes downstream of *AR* that are activated in the corpus spongiosum include *MAFB, CSRP2, CYP1B1, TIPARP*, as identified by our differential expression analysis between males and females (**Fig. 4c**). We also leveraged one of the publicly available mouse scRNA-seq datasets of the external genitalia to investigate potential shared regulators of urethral canalisation across species[^31^](https://paperpile.com/c/zlrttx/cdYvC). Since the dataset did not annotate a cell type equivalent to the *corpus spongiosum*, we first applied mutual nearest neighbours matching to identify the relevant cell population (**Methods**). Differential expression[^66^](https://paperpile.com/c/zlrttx/z59sL) within the murine equivalent of the *corpus spongiosum* between sexes revealed that *Mafb* and *Csrp2* are male-biassed in both species (**Extended Data Fig. 8h-l**).
- Prepuce (mesenchymal) cells (**Supplementary Figs. 1a-b**, **2a-b**, **3a-b**) are characterised by the expression of *SHOX*/*SHOX2*, with the sub-dermal prepuce acquiring the additional expression of *IRX1/IRX2* (**Extended Data Fig. 8a-b**).
- Endodermal-derived urethral epithelial cells (**Supplementary Figs. 1a-b**, **2a-b**, **3a-b**) share the expression of *SSH* and *FOXA1* with the epithelial cells derived from the urogenital sinus (which is also endodermal) but further show upregulation of uroplakins like *UPK3A.* In males, we also reported the upregulation of *SCGB1A1* and *PTPRD* (**Fig. 4d**), which in other tissues function as important mediators of canalised epithelial structures.
- Ectodermal-derived genital epidermis cells (**Supplementary Figs. 1a-b**, **2a-b**, **3a-b**) express *KRT1* and *KRTDAP* while cells in the preputial lamina upregulate *KRT14* and *WNT3*.

# **Supplementary Note 2: Müllerian rostro-caudal axis**

## *2.1 Deriving the axis from spatially-resolved transcriptomics data*

Because of the blurred boundaries during fetal development and our interest in detecting sequential changes in gene expression, we adopted the idea conceptualised in[^67^](https://paperpile.com/c/zlrttx/DL3f) of measuring distances from anatomical landmarks in the tissue to create a continuous spatial axis that spans the developing female reproductive system (hereby called *Müllerian rostro-caudal axis*). As the name implies, the *Müllerian rostro-caudal axis* follows the direction of elongation of the Müllerian ducts from the fallopian fimbriae to the end of the upper (Müllerian-derived) vagina. This approach not only facilitates the aggregation of data across multiple samples, enhancing the robustness of the biological conclusions, but also enables detailed examination of spatial subcompartments in a quantitative manner.

*10x Visium* and *ISS* data are characterised by distinct sampling frequencies (e.g. distance between adjacent spots, expressed in μm), which are independent of the imaging resolution (defined in pixels). However, to construct a *Müllerian rostro-caudal axis* that facilitates comparisons across technologies, one needs to define a common sampling frequency. This is achieved in *TissueTag*[*^67^*](https://paperpile.com/c/zlrttx/DL3f) by using a hexagonal point grid (HPG) with a predetermined sampling frequency, onto which pixel level annotations can be mapped. Hexagonal tiling results in a more uniform distribution of points across a given area, reducing variability in the distance between neighbouring points and offering a closer approximation to isotropy. The desired common sampling frequency can therefore be set as the distance between the centres of adjacent hexagons. Pixel-level annotations from imaging data (H&E image in *10x Visium* or “virtual” RGB image in *ISS*) are then mapped to the nearest vertex in the HPG.

The anatomical landmarks we employed for constructing the *Müllerian rostro-caudal axis* along with the *10x Visium Cytassist* and *ISS* samples that include them are reported in the table below. It is worth noting that we decided to exclude *10x Visium* (not *Cytassist*) samples for the purpose of constructing the *Müllerian rostro-caudal axis* due to their shallow sequencing depth (**Supplementary Note Table 2**). Since the primary usage of the *Müllerian rostro-caudal axis* is to explore gradients of gene expression and cell-cell communication across the female reproductive tract, including samples with shallow sequencing depth of a spot-based technology only increases noise.

| **Anatomical landmark** | **Spatially-resolved transcriptomics samples** |
| --- | --- |
| Fallopian tube fimbriae | HCA_F_RepTsp13902018 (*10x Visium Cytassist*), HCA_F_RepTsp14645816 (*10x Visium Cytassist*),  HCA_F_RepTsp15070470 (*10x Visium Cytassist*),  HCA_F_RepTsp15070471 (*10x Visium Cytassist*),  CM013 (*ISS*),  CM015 (*ISS*),  CM016 (*ISS*) |
| Fallopian-uterine junction (isthmus) | HCA_F_RepTsp13902018 (*10x Visium Cytassist*), HCA_F_RepTsp14645816 (*10x Visium Cytassist*),  HCA_F_RepTsp15070470 (*10x Visium Cytassist*),  HCA_F_RepTsp15070471 (*10x Visium Cytassist*),  CM013 (*ISS*),  CM015 (*ISS*),  CM016 (*ISS*) |
| Uterine fundus | HCA_F_RepTsp13902014 (*10x Visium Cytassist*), HCA_F_RepTsp13902016 (*10x Visium Cytassist*),  CM008 (*ISS*),  CM013 (*ISS*),  CM016 (*ISS*) |
| Uterine-upper (Müllerian-derived) vagina junction | HCA_F_RepTsp13902013 (*10x Visium Cytassist*),  HCA_F_RepTsp13902014 (*10x Visium Cytassist*),  HCA_F_RepTsp13902016 (*10x Visium Cytassist*), HCA_F_RepTsp13902015 (*10x Visium Cytassist*),  CM008 (*ISS*),  CM013 (*ISS*),  CM016 (*ISS*) |
| Upper (Müllerian-derived) vagina-vaginal plate (lower vagina) junction | HCA_F_RepTsp13902013 (*10x Visium Cytassist*), HCA_F_RepTsp13902015 (*10x Visium Cytassist*),  CM008 (*ISS*),  CM013 (*ISS*),  CM016 (*ISS*) |

**Supplementary Note Table 2**. Table summarising the anatomical landmarks used to derive the *Müllerian rostro-caudal axis* alongside the *10x Visium Cytassist* and *ISS* samples that include them.

Once anatomical landmarks have been annotated, distances between any given point on the HPG and each landmark were computed as the average Euclidean distance from the point to *k* nearest neighbours in the landmark. Distances to individual landmarks were then combined in order to obtain the desired continuous axis that spans the developing female reproductive tract. The full *Müllerian rostro-caudal axis* is derived as the concatenation of two axes: the *fallopian tube axis* (which spans the length of the fallopian tube, from the fimbriae to the fallopian-uterine junction) and the *uterovaginal canal axis* (from the uterine fundus to the upper vagina-vaginal plate junction, where mesodermal and endodermal vaginas join) (**Fig. 3a-d**).

However, due to the size limitations of the capture area of the *10x Visium* platform (6.5 x 6.5 mm), it can be unfeasible to capture the entire length of the uterovaginal canal. To overcome this limitation, consecutive sections containing the upper and lower portions of the uterovaginal canal for each sample were placed in two separate capture areas (**Fig. 3c**). For these samples, we computationally stitched the *10x* *Visium* data from the two consecutive capture areas (<https://github.com/Teichlab/visium_stitcher>) and computed the distances from the anatomical landmarks, now all present in the stitched image. Briefly, the H&E images of the consecutive *10x Visium* sections were manually overlapped using the image processing software *Fiji* (<https://imagej.net/plugins/trakem2/>). The transformation matrices that correspond to the manual operations of scaling, rotation and translation needed to obtain the desired overlap were exported from *Fiji* and applied as affine transformations to the H&E images in python. The new pixel positions of the H&E images were then transferred to the spot grid and the individual *10x Visium* *anndata* objects for consecutive sections were concatenated.

We then computed the *fallopian tube axis* and the *uterovaginal canal axis* as the normalised distance between the annotated landmarks. In the case of the *fallopian tube axis*, for instance, each point is assigned a value between -1 and +1, where if a point is near the fallopian fimbriae, it will have a value close to -1, while if it is approximately in between the fallopian tube fimbriae and the fallopian-uterine junction, it will be assigned a value close to 0. For convenience, the *fallopian tube axis* was rescaled from -2 (fimbriae) to 0 (fallopian-uterine junction), while the *uterovaginal canal axis* was rescaled from 0 (uterine fundus) to +4 (upper vagina-vaginal plate junction) (**Fig. 3d**). Analogous to the approach taken to derive the *Müllerian rostro-caudal axis* in *10x Visium* data, distances between landmarks annotated in the “virtual” RGB are used to compute the axis in *ISS* data.

## *2.2 Projecting the axis onto scRNA-seq data*

The Müllerian longitudinal axis in *ISS* data is of limited use for biological discovery because *ISS* only captures the expression of 171 genes. However, the single-cell resolved nature of the *ISS* technology makes it amenable to integration with scRNA-seq data via *kNN* graphs, as described in **Methods**. We therefore reasoned that we could leverage the same concept used for cell type annotation of *ISS* cells from scRNA-seq data to instead impute the position along the *Müllerian rostro-caudal axis* in scRNA-seq cells based on *ISS* data.

Given that the *Müllerian rostro-caudal axis* is constructed to investigate continuous changes in gene expression in the mesenchymal and epithelial compartments of the developing female internal genitalia, we subsetted both the *ISS* and scRNA-seq datasets to only cells belonging to these two compartments. Then, by modifying the code implemented in the pachter library (<https://iss-patcher.readthedocs.io/en/latest/>), the *ISS* dataset was set as the reference in which the *kNN* graph is initialised, and was used to find the nearest *ISS* neighbours (*k* = 15) for each scRNA-seq cell based on Euclidean distance. Finally, the average of the *Müllerian rostro-caudal axis* value of the 15 nearest *ISS* neighbours was taken as the imputed axis value for each scRNA-seq cell (**Extended Data Fig. 5c-d**). The following section further describes how the accuracy of the imputed axis was evaluated.

## *2.3 Prioritising spatially-variable genes along the axis*

Mesenchymal patterning of the Müllerian duct into its organ derivatives is known to be orchestrated by the sequential, rostro-caudal expression of a cluster of lumbosacral *HOX* code genes (*HOXA9/10/11/13*), which are presumably conserved across vertebrate species[^68^](https://paperpile.com/c/zlrttx/4nnzW). In addition to the *HOX* code, there might be other transcription factors that contribute to the spatial regionalisation of the Müllerian duct. Once the mesenchyme has differentiated, it signals to the adjacent epithelium how to differentiate accordingly, as has been demonstrated by tissue-recombinant experiments[^69^](https://paperpile.com/c/zlrttx/RPK4D). To investigate this patterning process in the human female reproductive tract, we adapted the *TradeSeq*[*^70^*](https://paperpile.com/c/zlrttx/0WSqF) framework and modelled gene expression along the measured and imputed *Müllerian rostro-caudal axis* for *10x Visium Cytassist* and scRNA-seq data, respectively. The continuous-valued *Müllerian rostro-caudal axi*s was therefore treated analogously to pseudotime.

### 2.3.1 Müllerian-derived mesenchyme

For each spot in the *10x Visium Cytassist* samples, we first computed the most abundant cell type annotation from the output of the deconvolution method *cell2location*[*^71^*](https://paperpile.com/c/zlrttx/gaThy) along with the corresponding fraction (**Methods**). Then, spots whose most abundant cell type was one of the relevant mesenchymal cell types (i.e. fallopian tube, uterus, cervix and upper vagina mesenchyme) and whose corresponding fraction was >= 0.6 were selected. We further filtered the resulting spots to keep only those also annotated as “mesenchyme” based on histological features. Mitochondrial, ribosomal and haemoglobin genes were removed from the mesenchymal spots x genes matrix before using it as input to *TradeSeq*[*^70^*](https://paperpile.com/c/zlrttx/0WSqF). Likewise, cells annotated as the relevant mesenchymal cell types were selected from scRNA-seq samples, and mitochondrial, ribosomal and haemoglobin genes were removed from the cells x genes matrix. Finally, in both datasets (*10x Visium Cytassist* and scRNA-seq) the *Müllerian rostro-caudal axis* was binned in 10 equally spaced bins, and mesenchymal spots/cells were downsampled to have equal coverage along the axis.

*TradeSeq* spline modelling was then performed independently on the *10x Visium Cytassist* and scRNA-seq mesenchymal matrices (knots = 10) (**Supplementary Table 8**). In both cases, all spots/cells were assigned a weight of 1 in association to the *Müllerian rostro-caudal axis*, as this is the only “lineage”. Using the *associationTest()*, which tests the null hypothesis that all smoother coefficients are equal to each other, we selected the genes whose average expression is significantly changing along the *Müllerian rostro-caudal axis* (p-value <= 0.001 and Wald statistics > 500). Fitted values for each selected gene across 100 equally spaced points along the axis were then extracted from the *TradeSeq*[*^70^*](https://paperpile.com/c/zlrttx/0WSqF) output.

To evaluate the agreement of the spatially variable genes identified in *10x Visium Cytassist* and scRNA-seq, we first took the intersection of the selected genes for each *TradeSeq* analysis. Then, for the genes that were identified as spatially variable in both technologies, we computed the cosine similarity of their fitted values along the *Müllerian rostro-caudal axis.* This analysis revealed that the fitted smoothers largely match between technologies, with approximately 80% of intersecting genes having a cosine similarity value >= 0.9 (**Extended Data Fig. 5e**). Such results also validate the accuracy of the imputed *Müllerian rostro-caudal axis* in scRNA-seq data.

We then further investigated the nature of the genes that were picked up as spatially variable by only one technology. Genes selected as spatially variable only by means of *10x Visium Cytassist* tend to be non-mesenchymal specific genes (e.g. immune or endothelial specific genes) that likely reflect contamination of the spot deemed as “mesenchymal” by other cell types. Conversely, genes that were identified as spatially variable only in the scRNA-seq dataset are bona fide mesenchymal-specific genes mostly expressed in the fallopian tube (e.g. *LGR5*, *TSPAN8*). Such genes are likely not selected in the *10x Visium Cytassist* dataset because the fallopian tube has a thin layer of mesenchyme and it is therefore more difficult to obtain “pure” mesenchymal spots in this developing organ. Based on this characterisation, we therefore decided to retain as spatially variable mesenchymal genes the union of the common and scRNA-seq specific genes.

The resulting list of spatially variable genes in the Müllerian-derived mesenchyme was then intersected with a curated list of human transcription factors to identify putative regulators responsible for mesenchymal patterning beyond the *HOX* code. The average, min-max normalised expression of such spatially variable mesenchymal transcription factors per bin of the imputed *Müllerian rostro-caudal axis* was visualised using a heatmap (**Fig. 3h**).

### 2.3.2 Müllerian-derived epithelium

Spatially variable genes and transcription factors in the developing Müllerian duct epithelium were prioritised following the strategy adopted for the mesenchyme described in the previous section (with the same parameters and thresholds). Briefly, epithelial spots in *10x Visium Cytassist* data were selected based on histological feature annotations and estimated fraction of deconvoluted cell type by *cell2location*. Mitochondrial, ribosomal and haemoglobin genes were removed from the epithelial spot/cell x genes matrices used as input to *TradeSeq*[*^70^*](https://paperpile.com/c/zlrttx/0WSqF), and *TradeSeq*’s *associationTest()* was used to select genes whose expression varies significantly along the *Müllerian rostro-caudal axis*.

Similar considerations as those outlined for the mesenchyme explain the discrepancy in the spatially variable genes identified in *10x Visium Cytassis*t and scRNA-seq data, and hence we decided to again take the union of the common and scRNA-seq specific genes as the final list of *bona fide* spatially variable genes along the differentiating Müllerian duct epithelium. Spatially variable transcription factors along the axis were visualised with a heatmap of their expression averaged and min-max normalised per bin (**Extended Data Fig. 6k**).

## *2.4 Prioritising spatially-variable mesenchymal-epithelial interactions along the axis*

Mesenchymal-epithelial interactions are what drive the epithelium of the developing female reproductive tract to acquire its organ-specific phenotype based on spatial context[^69^](https://paperpile.com/c/zlrttx/RPK4D). However, epithelial-derived signals are also important for mesenchymal cells’ growth and stability, and hence communication is considered to be bidirectional[^69^](https://paperpile.com/c/zlrttx/RPK4D). We therefore sought to investigate how these interactions between the mesenchymal and epithelial compartment vary along the length of the *Müllerian rostro-caudal axis*. Only the scRNA-seq data was used for this analysis.

First, we binned the *Müllerian rostro-caudal axis* into 10 equally spaced bins. Among the spatially variable genes identified in the mesenchymal compartment, we retained those that appear as interacting partners in the cell-cell communication database *CellPhoneDB* v5[^72^](https://paperpile.com/c/zlrttx/8a07). Then, for each interaction listed in *CellPhoneDB* v5[^72^](https://paperpile.com/c/zlrttx/8a07) in which one interacting partner is a spatially variable gene in the mesenchyme, we perform the following filtering steps to evaluate if it can be considered a spatially variable interaction along the *Müllerian rostro-caudal axis*  (**Fig. 3i**, **Extended Data Fig. 6e,g**).

1. Compute the average expression of the mesenchymal spatially-variable gene per bin in the *Müllerian rostro-caudal axis*;
2. Min-max normalise the average expression per bin;
3. If there are at least 2 consecutive bins that have a normalised expression >= 0.4, proceed to **4**, and flag the bins that satisfy the condition as having the mesenchymal partner upregulated.
4. In the epithelial compartment, compute the proportion of cells in each bin of the axis that express the corresponding partner (or all subunits if multimeric);
5. If there are at least 2 consecutive bins where >= 20% of epithelial cells express the interacting partner (or all subunits if multimeric) and which are also flagged as having the mesenchymal partner upregulated (as computed in **3**), then consider the mesenchymal-to-epithelial interaction is being spatially variable along the *Müllerian rostro-caudal axis*.

# **Supplementary Note 3: Wolffian rostro-caudal axis**

Analogous to the *Müllerian rostro-caudal axis*, the *Wolffian rostro-caudal axis* was conceptualised to span the length of the epididymis and the initial segment of the vas deferens (due to damage to the vas deferens during sample dissections we do not have access to the caudal part of vas deferens nor seminal vesicle). The chosen anatomical landmarks were the efferent ductules and the initial segment of the vas deferens, and the *Wolffian rostro-caudal axis* was computed as the normalised Euclidean distance between the two (**Supplementary Note Table 3**). The axis values were re-scaled to -2 to +2 for consistency with the *Müllerian rostro-caudal axis*. Only *10x Visium Cytassist* samples were used for the *Wolffian rostro-caudal axis* as we only have one suitable *ISS* sample and hence the imputation of the axis onto scRNA-seq would not be robust.

| **Anatomical landmark** | ***10x Visium Cytassist* samples** |
| --- | --- |
| Efferent ductules | HCA_F_RepTsp14707486,  HCA_F_RepTsp14733322,  HCA_F_RepTsp15395356 |
| Vas deferens | HCA_F_RepTsp14707486*,*  HCA_F_RepTsp14733322*,*  HCA_F_RepTsp15395356 |

**Supplementary Note Table 3**. Table summarising the anatomical landmarks used to derive the *Wolffian rostro-caudal axis* alongside the *10x Visium Cytassist* that include them.

Spatially-variable genes along the *Wolffian rostro-caudal axis* were prioritised using an approach similar to that applied to the measured *Müllerian rostro-caudal axis* in *10x Visium* *Cytassist* data (**Supplementary Note 2.3**). Briefly, we first divided the *Wolffian rostro-caudal axis* into 6 equally spaced bins, and mesenchymal and epithelial spots were downsampled independently to ensure an equal number of spots per bin. Using *TradeSeq*[*^70^*](https://paperpile.com/c/zlrttx/0WSqF), we then modelled gene expression continuously along the axis in the mesenchymal and epithelial spots independently using 6 knots (**Supplementary Table 8**). Genes with significant expression changes along the Wolffian rostro-caudal axis were identified using *TradeSeq*'s *associationTest(),* selecting those with a p-value < 0.001 and a logFC > 0.5. To further refine the resulting list to bona fide mesenchymal or epithelial spatially-variable genes, only genes with expression specific to the mesenchyme or epithelium were retained by comparing the expression of these spatially-variable genes against all other cell types in the scRNA-seq dataset. This step is particularly relevant in male (as compared to female) samples as the epididymis and vas deferens are very convoluted and hence the number of “pure” mesenchymal or epithelial spots is lower.

We prioritised the mesenchymal-epithelial interactions that are likely to drive epithelial differentiation during the process of Wolffian duct regionalisation using the same approach described in **Supplementary Note 2.4** but based on *10x Visium Cytassist* data instead of scRNA-seq data (**Extended Data Figs. 6i-j**).

# **Supplementary Note 4: Intra-organ regionalisation of fallopian tube and epididymis**

Both the *fallopian* and *epididymal* *axes* were computed as part of the *Müllerian* and *Wolffian* *rostro-caudal axes*, respectively, and were utilised to investigate intra-organ differences in gene expression in the non-ciliated epithelium. The non-ciliated epithelium is the cellular compartment that exhibits morphological and functional regionalisation in adulthood, reflecting the need for specialised secretions into the lumen of the organ to enable the various steps of sperm maturation (in the epididymis[^60^](https://paperpile.com/c/zlrttx/w8z2P)) and capacitation (in the fallopian tube[^73,74^](https://paperpile.com/c/zlrttx/DHKpH+5QxYW)). Moreover, non-ciliated epithelial cells from the fallopian fimbriae have recently been identified as the cell-of-origin of the majority of cases of high-grade serous ovarian cancer[^75^](https://paperpile.com/c/zlrttx/qFpFN). We therefore used the rostro-caudal axis framework to see if we could see evidence of such intra-organ regionalisation in the non-ciliated epithelium of the fallopian tube and epididymis already during fetal development.

The *fallopian* and *epididymal axes* were divided into 3 equally spaced bins and non-ciliated epithelial spots were downsampled to obtain an equal number of spots per bin. Spatially-variable genes were prioritised using the *associationTest()* in *TradeSeq*[*^70^*](https://paperpile.com/c/zlrttx/0WSqF) (5 knots) and selecting those with p-value < 0.001 and logFC > 0.5 (**Supplementary Table 9**). Only *10x Visium Cytassist* data was used in this case as differences in gene expression within these organs are subtle and hence the imputation in scRNA-seq might lack accuracy. However, as is the case for the *Wolffian rostro-caudal axis* (**Supplementary Note 3**) this means that some genes identified as spatially variable might be from cell types other than the non-ciliated epithelium. We therefore used the scRNA-seq to compare the average expression of each prioritised gene in secretory epithelial cells vs each other cell type to make sure that the expression was indeed specific to the non-ciliated epithelium (**Fig. 3j**, **Extended Data Fig. 7k**).

# Supplementary Note 5: Discussion

Our findings provide a foundational framework for understanding the signalling cues, rostro-caudal positional identity, and gene expression dynamics that guide the lineage-specification of reproductive tract tissues in both females and males. This work contributes to closing the knowledge gap in human reproductive tract development and opens new avenues for translational research in reproductive health.

In early development, we identified novel male-specific genes likely involved in Müllerian duct regression, whose specific expression we further validated via smFISH. Our findings reinforce the role of WNT inhibitors, as reported in murine functional studies[^76,77^](https://paperpile.com/c/zlrttx/R2jEK+641UP), and provide a molecular foundation for autophagy as an underlying mechanism in this process, a hypothesis previously suggested based on electron microscopy observations[^78^](https://paperpile.com/c/zlrttx/lBFkD).

Following the regression of the unmatched ducts, the differentiation of sex-specific organs from the remaining ducts relies on the precise establishment of spatial boundaries between the organs. To determine what TFs and cell-cell communication events drive ductal regionalisation in humans, we computationally derived *Müllerian* and *Wolffian rostro-caudal axes* using our spatial transcriptomics data and by adapting the *OrganAxis* framework[^67^](https://paperpile.com/c/zlrttx/DL3f). This approach was necessary as subtle differences in gene expression were not detectable in single-cell transcriptomic data of dissociated samples and the anatomical intra-organ regionalisation was not yet evident at these developmental stages. Our analysis revealed that thoracic *HOX* genes are expressed in the upper fallopian tubes and epididymis, challenging the traditional view that mesenchymal regionalisation is governed solely by lumbosacral *HOX* genes. Moreover, we uncovered novel TFs potentially regulating mesenchymal differentiation, some of which are shared between Müllerian and Wolffian-derived mesenchymal cells (e.g., *MEIS2* and *RORB* in the uterus and upper vas deferens), while others are specific to one sex (e.g. *GATA6/PROX1* in the fallopian tube and *FOXC2/ALX1* in the epididymis). This finding suggests that female and male organs at analogous rostro-caudal positions share common regulatory programs, while their specific roles demand unique transcriptional regulators, though further research is needed to fully understand the interplay between these mechanisms. Our comparison, however, is limited by the inability to fully reconstruct the Wolffian duct axis due to damage to the vas deferens during dissections.

We discovered key paracrine mesenchymal-derived signals along the Müllerian and Wolffian duct axes. For instance, *BMP4/7* are spatially confined to the upper vagina, whereas *GDF7* is more broadly expressed in the uterocervix and upper vagina, indicating distinct roles for *BMP* ligands across the female reproductive tract. This aligns with observations in the adult endometrium, where *GDF7*, but not *BMP4/7*, signalling is present[^55^](https://paperpile.com/c/zlrttx/zgll). Future studies utilising high-throughput, three-dimensional gene expression profiling will be invaluable for determining whether these spatial signalling dynamics are established during the early phases of Müllerian duct development. Our findings can be incorporated into emerging *in vitro* models of the female and male reproductive tracts[^79,80^](https://paperpile.com/c/zlrttx/wd8Ch+57E2s), which are particularly needed due to differences in gene expression between murine and human reproductive organs, as well as the significant delay in advancing reproductive models compared to other bodily systems.

Furthermore, we identified the genes likely driving the regionalisation of the fetal fallopian tube and epididymal non-ciliated epithelium, revealing that gene expression gradients crucial for sperm maturation in adulthood are already established prenatally. This contrasts with the uterocervical epithelium, which remains transcriptionally homogeneous until at least 21 PCW, suggesting that its adult structure may depend on hormonal influences later in life. While intra-organ regionalisation has been well-documented in tissues like the gut[^81^](https://paperpile.com/c/zlrttx/BYwzv) and liver[^82,83^](https://paperpile.com/c/zlrttx/sW0o2+eowqu), and there is evidence suggesting that this process begins during fetal development in mouse gut[^84^](https://paperpile.com/c/zlrttx/Aywh8), it has not been extensively explored in female reproductive tissues, especially in the fallopian tubes. Our findings not only enhance our understanding of basic biology but also have implications for fertility conditions affecting the fallopian tubes that may be determined *in utero*. Moreover, among the transcriptomic signatures upregulated in both fetal and adult fallopian fimbriae, we uncovered genes such as *PNOC* and *CLDN6*, which are diagnostic markers and therapeutic targets of high-grade serous ovarian cancer (HGSOC)[^85,86^](https://paperpile.com/c/zlrttx/v8LeP+t519h), a malignancy known to originate in the adult fimbriae[^75^](https://paperpile.com/c/zlrttx/qFpFN). This highlights the potential of utilising fetal data, where continuous spatial modelling of gene expression is feasible due to reduced sample size, to uncover new genes implicated in HGSOC.

To investigate sexual dimorphism in the external genitalia, we focused on the downstream genes regulating androgen-driven urethral canalisation in males during the masculinisation programming window, a process disrupted in hypospadias[^30,87^](https://paperpile.com/c/zlrttx/T7wiC+hb7y6). We found male-biased gene expression in both the mesenchymal *corpus spongiosum* and urethral epithelium, as well as sexually dimorphic interactions between these cell types. Among the male-biased genes in the *corpus spongiosum*, we identified *MAFB*, which is the only mesenchymal, androgen-responsive gene potentially involved in urethral canalisation that has been validated with a functional study in mice[^88^](https://paperpile.com/c/zlrttx/Bi52C). Given that surgical reconstruction remains the primary treatment for hypospadias, but is limited by insufficient graft material, our insights into the molecular drivers of urethral canalisation could guide tissue engineering efforts to develop more effective graft materials[^89,90^](https://paperpile.com/c/zlrttx/36FEM+huZEM).

Lastly, we used our atlas to assess *in silico* the potential toxicity of endocrine disruptors found in everyday products and commonly prescribed drugs on reproductive development, where many congenital abnormalities remain unexplained. For instance, *ESR1* was upregulated in the human uterovaginal and epididymis epithelia, consistently with the known trans-generational effects of its agonist BPA, which impairs vaginal and epididymal development in rodents[^91,92^](https://paperpile.com/c/zlrttx/z369m+nkR2u). Consistent with these predictions, fetal uterine epithelial organoids exposed to BPA and BBP upregulated *PGR* and other estrogen-responsive genes, indicating that the fetal uterine epithelium has the potential to respond to exogenous estrogenic compounds, mimicking transcriptomic changes seen in the adult proliferative phase. Future work combining organoid models with functional assays is needed to validate these predictions and reveal the full extent of their impact on reproductive development.

#

# Supplementary References

1. [Robboy, S. J., Kurita, T., Baskin, L. & Cunha, G. R. New insights into human female reproductive tract development. *Differentiation* **97**, 9–22 (2017).](http://paperpile.com/b/zlrttx/TFbHY)

2. [Garcia-Alonso, L. *et al.* Single-cell roadmap of human gonadal development. *Nature* **607**, 540–547 (2022).](http://paperpile.com/b/zlrttx/bDFDH)

3. [Young, M. D. & Behjati, S. SoupX removes ambient RNA contamination from droplet-based single-cell RNA sequencing data. *Gigascience* **9**, giaa151 (2020).](http://paperpile.com/b/zlrttx/8SeZi)

4. [Miyamoto, N., Yoshida, M., Kuratani, S., Matsuo, I. & Aizawa, S. Defects of urogenital development in mice lacking Emx2. *Development* **124**, 1653–1664 (1997).](http://paperpile.com/b/zlrttx/uF2II)

5. [Pelletier, J. *et al.* Expression of the Wilms’ tumor gene WT1 in the murine urogenital system. *Genes Dev.* **5**, 1345–1356 (1991).](http://paperpile.com/b/zlrttx/bZLD5)

6. [Pelletier, J. *et al.* WT1 mutations contribute to abnormal genital system development and hereditary Wilms’ tumour. *Nature* **353**, 431–434 (1991).](http://paperpile.com/b/zlrttx/4wQnP)

7. [Khandekar, M., Suzuki, N., Lewton, J., Yamamoto, M. & Engel, J. D. Multiple, distant Gata2 enhancers specify temporally and tissue-specific patterning in the developing urogenital system. *Mol. Cell. Biol.* **24**, 10263–10276 (2004).](http://paperpile.com/b/zlrttx/5PnDw)

8. [Zhou, Y. *et al.* Rescue of the embryonic lethal hematopoietic defect reveals a critical role for GATA-2 in urogenital development. *EMBO J.* **17**, 6689–6700 (1998).](http://paperpile.com/b/zlrttx/MyKp2)

9. [Orvis, G. D. & Behringer, R. R. Cellular mechanisms of Müllerian duct formation in the mouse. *Dev. Biol.* **306**, 493–504 (2007).](http://paperpile.com/b/zlrttx/Fv6Ey)

10. [Kobayashi, A., Shawlot, W., Kania, A. & Behringer, R. R. Requirement of Lim1 for female reproductive tract development. *Development* **131**, 539–549 (2004).](http://paperpile.com/b/zlrttx/h9iK3)

11. [Rudat, C. *et al.* Upk3b is dispensable for development and integrity of urothelium and mesothelium. *PLoS One* **9**, e112112 (2014).](http://paperpile.com/b/zlrttx/PU5G1)

12. [Huang, H. *et al.* Mesothelial cell-derived antigen-presenting cancer-associated fibroblasts induce expansion of regulatory T cells in pancreatic cancer. *Cancer Cell* **40**, 656–673.e7 (2022).](http://paperpile.com/b/zlrttx/uUlOC)

13. [Parr, B. A. & McMahon, A. P. Sexually dimorphic development of the mammalian reproductive tract requires Wnt-7a. *Nature* **395**, 707–710 (1998).](http://paperpile.com/b/zlrttx/5bqpE)

14. [Miller, C. & Sassoon, D. A. Wnt-7a maintains appropriate uterine patterning during the development of the mouse female reproductive tract. *Development* **125**, 3201–3211 (1998).](http://paperpile.com/b/zlrttx/5Cq8U)

15. [Dinh, H. Q. *et al.* Single-cell transcriptomics identifies gene expression networks driving differentiation and tumorigenesis in the human fallopian tube. *Cell Rep.* **35**, 108978 (2021).](http://paperpile.com/b/zlrttx/bfgQw)

16. [Taelman, J. *et al.* Characterization of the human fetal gonad and reproductive tract by single-cell transcriptomics. *Dev. Cell* **59**, 529–544.e5 (2024).](http://paperpile.com/b/zlrttx/qrokP)

17. [Carroll, T. J., Park, J.-S., Hayashi, S., Majumdar, A. & McMahon, A. P. Wnt9b plays a central role in the regulation of mesenchymal to epithelial transitions underlying organogenesis of the mammalian urogenital system. *Dev. Cell* **9**, 283–292 (2005).](http://paperpile.com/b/zlrttx/pDMfB)

18. [Grote, D. *et al.* Gata3 acts downstream of beta-catenin signaling to prevent ectopic metanephric kidney induction. *PLoS Genet.* **4**, e1000316 (2008).](http://paperpile.com/b/zlrttx/bwk6x)

19. [Vainio, S., Heikkilä, M., Kispert, A., Chin, N. & McMahon, A. P. Female development in mammals is regulated by Wnt-4 signalling. *Nature* **397**, 405–409 (1999).](http://paperpile.com/b/zlrttx/LUZLr)

20. [Mericskay, M., Kitajewski, J. & Sassoon, D. Wnt5a is required for proper epithelial-mesenchymal interactions in the uterus. *Development* **131**, 2061–2072 (2004).](http://paperpile.com/b/zlrttx/NgAx2)

21. [Arango, N. A. *et al.* A mesenchymal perspective of Müllerian duct differentiation and regression in Amhr2-lacZ mice. *Mol Reprod Dev* **75**, 1154–1162 (2008).](http://paperpile.com/b/zlrttx/k2eI)

22. [Podlasek, C. A., Barnett, D. H., Clemens, J. Q., Bak, P. M. & Bushman, W. Prostate development requires Sonic hedgehog expressed by the urogenital sinus epithelium. *Dev. Biol.* **209**, 28–39 (1999).](http://paperpile.com/b/zlrttx/lI8mq)

23. [Gao, N. *et al.* Forkhead box A1 regulates prostate ductal morphogenesis and promotes epithelial cell maturation. *Development* **132**, 3431–3443 (2005).](http://paperpile.com/b/zlrttx/Fl9wO)

24. [Shen, J. *et al.* Immunohistochemical expression analysis of the human fetal lower urogenital tract. *Differentiation* **103**, 100–119 (2018).](http://paperpile.com/b/zlrttx/nlRm7)

25. [Diez-Roux, G. *et al.* A high-resolution anatomical atlas of the transcriptome in the mouse embryo. *PLoS Biol.* **9**, (2011).](http://paperpile.com/b/zlrttx/XP1OO)

26. [Takeda, H., Oike, Y. & Sakakura, T. Immunofluorescent localization of tenascin during development of the mouse urogenital sinus: possible involvement in genital duct morphogenesis. *Differentiation* **39**, 131–138 (1988).](http://paperpile.com/b/zlrttx/ZmsU)

27. [Lee, D.-H. *et al.* Androgen action in cell fate and communication during prostate development at single-cell resolution. *Development* **148**, (2021).](http://paperpile.com/b/zlrttx/K7ww)

28. [Nayerpour Dizaj, T. *et al.* Significance of PSCA as a novel prognostic marker and therapeutic target for cancer. *Cancer Cell Int* **24**, 135 (2024).](http://paperpile.com/b/zlrttx/oI6i)

29. [Hustler, A. *et al.* Differential transcription factor expression by human epithelial cells of buccal and urothelial derivation. *Exp Cell Res* **369**, 284–294 (2018).](http://paperpile.com/b/zlrttx/VIlV)

30. [Baskin, L. *et al.* Development of the human penis and clitoris. *Differentiation* **103**, 74–85 (2018).](http://paperpile.com/b/zlrttx/hb7y6)

31. [Amato, C. M. & Yao, H. H.-C. Developmental and sexual dimorphic atlas of the prenatal mouse external genitalia at the single-cell level. *Proc. Natl. Acad. Sci. U. S. A.* **118**, (2021).](http://paperpile.com/b/zlrttx/cdYvC)

32. [Armfield, B. A. & Cohn, M. J. Single cell transcriptomic analysis of external genitalia reveals complex and sexually dimorphic cell populations in the early genital tubercle. *Dev. Biol.* **477**, 145–154 (2021).](http://paperpile.com/b/zlrttx/9mj4k)

33. [Kajimoto, M. *et al.* Androgen/Wnt/β-catenin signal axis augments cell proliferation of the mouse erectile tissue, corpus cavernosum. *Congenit. Anom.*  **62**, 123–133 (2022).](http://paperpile.com/b/zlrttx/zgIgN)

34. [Sreenivasan, R. *et al.* Altered SOX9 genital tubercle enhancer region in hypospadias. *J. Steroid Biochem. Mol. Biol.* **170**, 28–38 (2017).](http://paperpile.com/b/zlrttx/3kDn6)

35. [Yin, Y., Haller, M., Li, T. & Ma, L. Development of an in-vitro high-throughput screening system to identify modulators of genitalia development. *PNAS Nexus* **2**, gac300 (2023).](http://paperpile.com/b/zlrttx/GKBgq)

36. [Chiu, H. S. *et al.* Comparative gene expression analysis of genital tubercle development reveals a putative appendicular Wnt7 network for the epidermal differentiation. *Dev Biol* **344**, 1071–1087 (2010).](http://paperpile.com/b/zlrttx/Jgif)

37. [Bellessort, B. *et al.* Dlx5 and Dlx6 control uterine adenogenesis during post-natal maturation: possible consequences for endometriosis. *Hum. Mol. Genet.* **25**, 97–108 (2016).](http://paperpile.com/b/zlrttx/lab3V)

38. [Pavlova, A., Boutin, E., Cunha, G. & Sassoon, D. Msx1 (Hox-7.1) in the adult mouse uterus: cellular interactions underlying regulation of expression. *Development* **120**, 335–345 (1994).](http://paperpile.com/b/zlrttx/rzvUY)

39. [Kurita, T. & Cunha, G. R. Roles of p63 in differentiation of Müllerian duct epithelial cells. *Ann. N. Y. Acad. Sci.* **948**, 9–12 (2001).](http://paperpile.com/b/zlrttx/DeyrF)

40. [Cunha, G. R. *et al.* Development of the human female reproductive tract. *Differentiation* **103**, 46–65 (2018).](http://paperpile.com/b/zlrttx/u3urK)

41. [Leir, S.-H., Yin, S., Kerschner, J. L., Cosme, W. & Harris, A. An atlas of human proximal epididymis reveals cell-specific functions and distinct roles for CFTR. *Life Sci Alliance* **3**, (2020).](http://paperpile.com/b/zlrttx/ulMrl)

42. [Plyler, Z. E. *et al.* Non-obstructive vas deferens and epididymis loss in cystic fibrosis rats. *Mech Dev* **155**, 15–26 (2019).](http://paperpile.com/b/zlrttx/yd6V)

43. [Mirosevich, J., Gao, N. & Matusik, R. J. Expression of Foxa transcription factors in the developing and adult murine prostate. *Prostate* **62**, 339–352 (2005).](http://paperpile.com/b/zlrttx/pC7w)

44. [Combes, A. N. *et al.* Single cell analysis of the developing mouse kidney provides deeper insight into marker gene expression and ligand-receptor crosstalk. *Development* **146**, (2019).](http://paperpile.com/b/zlrttx/8zr0G)

45. [Bais, A. S. *et al.* Single-cell RNA sequencing reveals differential cell cycle activity in key cell populations during nephrogenesis. *Sci. Rep.* **11**, 22434 (2021).](http://paperpile.com/b/zlrttx/lN9sp)

46. [Hochane, M. *et al.* Single-cell transcriptomics reveals gene expression dynamics of human fetal kidney development. *PLoS Biol* **17**, e3000152 (2019).](http://paperpile.com/b/zlrttx/I2wz)

47. [Markadieu, N. & Delpire, E. Physiology and pathophysiology of SLC12A1/2 transporters. *Pflugers Arch.* **466**, 91–105 (2014).](http://paperpile.com/b/zlrttx/I3Xci)

48. [Doné, S. C. *et al.* Nephrin is involved in podocyte maturation but not survival during glomerular development. *Kidney Int.* **73**, 697–704 (2008).](http://paperpile.com/b/zlrttx/Jt7mK)

49. [Kestilä, M. *et al.* Positionally cloned gene for a novel glomerular protein--nephrin--is mutated in congenital nephrotic syndrome. *Mol. Cell* **1**, 575–582 (1998).](http://paperpile.com/b/zlrttx/86Zun)

50. [Wharram, B. L. *et al.* Altered podocyte structure in GLEPP1 (Ptpro)-deficient mice associated with hypertension and low glomerular filtration rate. *J. Clin. Invest.* **106**, 1281–1290 (2000).](http://paperpile.com/b/zlrttx/McIEp)

51. [Neirijnck, Y. *et al.* Single-cell transcriptomic profiling redefines the origin and specification of early adrenogonadal progenitors. *Cell Rep.* **42**, 112191 (2023).](http://paperpile.com/b/zlrttx/gryk0)

52. [Le Floch, E. *et al.* Identification of risk loci for primary aldosteronism in genome-wide association studies. *Nat. Commun.* **13**, 5198 (2022).](http://paperpile.com/b/zlrttx/yGWcD)

53. [Weigert, M. *et al.* A cell atlas of the human fallopian tube throughout the menstrual cycle and menopause. *Nat Commun* **16**, 372 (2025).](http://paperpile.com/b/zlrttx/VHm4)

54. [Roly, Z. Y. *et al.* Transcriptional landscape of the embryonic chicken Müllerian duct. *BMC Genomics* **21**, 688 (2020).](http://paperpile.com/b/zlrttx/NnzLm)

55. [Marečková, M. *et al.* An integrated single-cell reference atlas of the human endometrium. *Nat. Genet.* **56**, 1925–1937 (2024).](http://paperpile.com/b/zlrttx/zgll)

56. [Fu, N. Y. *et al.* Identification of quiescent and spatially restricted mammary stem cells that are hormone responsive. *Nat Cell Biol* **19**, 164–176 (2017).](http://paperpile.com/b/zlrttx/x2cK)

57. [Taylor, H. S., Vanden Heuvel, G. B. & Igarashi, P. A conserved Hox axis in the mouse and human female reproductive system: late establishment and persistent adult expression of the Hoxa cluster genes. *Biol Reprod* **57**, 1338–1345 (1997).](http://paperpile.com/b/zlrttx/lb9Z)

58. [Wilson, J. D., Griffin, J. E. & Russell, D. W. Steroid 5 alpha-reductase 2 deficiency. *Endocr. Rev.* **14**, 577–593 (1993).](http://paperpile.com/b/zlrttx/YJ2O)

59. [Speiser, P. W. *et al.* Congenital adrenal hyperplasia due to steroid 21-hydroxylase deficiency: An Endocrine Society clinical practice guideline. *J. Clin. Endocrinol. Metab.* **103**, 4043–4088 (2018).](http://paperpile.com/b/zlrttx/4FMs)

60. [Sullivan, R., Légaré, C., Lamontagne-Proulx, J., Breton, S. & Soulet, D. Revisiting structure/functions of the human epididymis. *Andrology* **7**, 748–757 (2019).](http://paperpile.com/b/zlrttx/w8z2P)

61. [Suzuki, K. *et al.* Embryonic development of mouse external genitalia: insights into a unique mode of organogenesis. *Evol. Dev.* **4**, 133–141 (2002).](http://paperpile.com/b/zlrttx/Pmp5d)

62. [Grumbach, M. M. & Ducharme, J. R. The effects of androgens on fetal sexual development: androgen-induced female pseudohermaphrodism. *Fertil. Steril.* **11**, 157–180 (1960).](http://paperpile.com/b/zlrttx/u0Mrm)

63. [Hynes, P. J. & Fraher, J. P. The development of the male genitourinary system: II. The origin and formation of the urethral plate. *Br. J. Plast. Surg.* **57**, 112–121 (2004).](http://paperpile.com/b/zlrttx/XB3j2)

64. [Baskin, L. S. *et al.* Anatomical studies of the human clitoris. *J. Urol.* **162**, 1015–1020 (1999).](http://paperpile.com/b/zlrttx/PPRnH)

65. [Liu, X. *et al.* Human glans and preputial development. *Differentiation* **103**, 86–99 (2018).](http://paperpile.com/b/zlrttx/ZTEnZ)

66. [Love, M. I., Huber, W. & Anders, S. Moderated estimation of fold change and dispersion for RNA-seq data with DESeq2. *Genome Biol.* **15**, 1–21 (2014).](http://paperpile.com/b/zlrttx/z59sL)

67. [Yayon, N. *et al.* A spatial human thymus cell atlas mapped to a continuous tissue axis. *Nature* **635**, 708–718 (2024).](http://paperpile.com/b/zlrttx/DL3f)

68. [Du, H. & Taylor, H. S. The Role of Hox Genes in Female Reproductive Tract Development, Adult Function, and Fertility. *Cold Spring Harb. Perspect. Med.* **6**, (2016).](http://paperpile.com/b/zlrttx/4nnzW)

69. [Cunha, G. R. *et al.* Mesenchymal-epithelial interactions in sex differentiation. *Hum. Genet.* **58**, 68–77 (1981).](http://paperpile.com/b/zlrttx/RPK4D)

70. [Van den Berge, K. *et al.* Trajectory-based differential expression analysis for single-cell sequencing data. *Nat. Commun.* **11**, 1201 (2020).](http://paperpile.com/b/zlrttx/0WSqF)

71. [Kleshchevnikov, V. *et al.* Cell2location maps fine-grained cell types in spatial transcriptomics. *Nat. Biotechnol.* **40**, 661–671 (2022).](http://paperpile.com/b/zlrttx/gaThy)

72. [Troulé, K. *et al.* CellPhoneDB v5: inferring cell-cell communication from single-cell multiomics data. *Nat. Protoc.* (2025) doi:](http://paperpile.com/b/zlrttx/8a07)[10.1038/s41596-024-01137-1](http://dx.doi.org/10.1038/s41596-024-01137-1)[.](http://paperpile.com/b/zlrttx/8a07)

73. [Ulrich, N. D. *et al.* Cellular heterogeneity of human fallopian tubes in normal and hydrosalpinx disease states identified using scRNA-seq. *Dev. Cell* **57**, 914–929.e7 (2022).](http://paperpile.com/b/zlrttx/DHKpH)

74. [Lengyel, E. *et al.* A molecular atlas of the human postmenopausal fallopian tube and ovary from single-cell RNA and ATAC sequencing. *Cell Rep.* **41**, 111838 (2022).](http://paperpile.com/b/zlrttx/5QxYW)

75. [Erickson, B. K., Conner, M. G. & Landen, C. N., Jr. The role of the fallopian tube in the origin of ovarian cancer. *Am. J. Obstet. Gynecol.* **209**, 409–414 (2013).](http://paperpile.com/b/zlrttx/qFpFN)

76. [Park, J. H. *et al.* Induction of WNT inhibitory factor 1 expression by Müllerian inhibiting substance/AntiMullerian hormone in the Müllerian duct mesenchyme is linked to Müllerian duct regression. *Dev. Biol.* **386**, 227 (2014).](http://paperpile.com/b/zlrttx/R2jEK)

77. [Cate, R. L. Anti-Müllerian Hormone Signal Transduction involved in Müllerian Duct Regression. *Front. Endocrinol.*  **13**, 905324 (2022).](http://paperpile.com/b/zlrttx/641UP)

78. [Dyche, W. J. A comparative study of the differentiation and involution of the mullerian duct and wolffian duct in the male and female fetal mouse. *J. Morphol.* **162**, 175–209 (1979).](http://paperpile.com/b/zlrttx/lBFkD)

79. [Chumduri, C. & Turco, M. Y. Organoids of the female reproductive tract. *J. Mol. Med.*  **99**, 531–553 (2021).](http://paperpile.com/b/zlrttx/wd8Ch)

80. [Cyr, D. G. & Pinel, L. Emerging organoid models to study the epididymis in male reproductive toxicology. *Reprod. Toxicol.* **112**, 88–99 (2022).](http://paperpile.com/b/zlrttx/57E2s)

81. [Harnik, Y. *et al.* A spatial expression atlas of the adult human proximal small intestine. *Nature* (2024) doi:](http://paperpile.com/b/zlrttx/BYwzv)[10.1038/s41586-024-07793-3](http://dx.doi.org/10.1038/s41586-024-07793-3)[.](http://paperpile.com/b/zlrttx/BYwzv)

82. [Ben-Moshe, S. *et al.* Spatial sorting enables comprehensive characterization of liver zonation. *Nat Metab* **1**, 899–911 (2019).](http://paperpile.com/b/zlrttx/sW0o2)

83. [Hildebrandt, F. *et al.* Spatial Transcriptomics to define transcriptional patterns of zonation and structural components in the mouse liver. *Nat. Commun.* **12**, 7046 (2021).](http://paperpile.com/b/zlrttx/eowqu)

84. [Maimets, M. *et al.* Mesenchymal-epithelial crosstalk shapes intestinal regionalisation via Wnt and Shh signalling. *Nat. Commun.* **13**, 715 (2022).](http://paperpile.com/b/zlrttx/Aywh8)

85. [Hibbs, K. *et al.* Differential Gene Expression in Ovarian Carcinoma : Identification of Potential Biomarkers. *Am. J. Pathol.* **165**, 397 (2004).](http://paperpile.com/b/zlrttx/v8LeP)

86. [McDermott, M. S. J. *et al.* Preclinical Efficacy of the Antibody-Drug Conjugate CLDN6-23-ADC for the Treatment of CLDN6-Positive Solid Tumors. *Clin Cancer Res* **29**, 2131–2143 (2023).](http://paperpile.com/b/zlrttx/t519h)

87. [Blaschko, S. D., Cunha, G. R. & Baskin, L. S. Molecular mechanisms of external genitalia development. *Differentiation* **84**, 261–268 (2012).](http://paperpile.com/b/zlrttx/T7wiC)

88. [Suzuki, K. *et al.* Sexually dimorphic expression of Mafb regulates masculinization of the embryonic urethral formation. *Proc. Natl. Acad. Sci. U. S. A.* **111**, 16407–16412 (2014).](http://paperpile.com/b/zlrttx/Bi52C)

89. [Abbas, T. O., Mahdi, E., Hasan, A., AlAnsari, A. & Pennisi, C. P. Current Status of Tissue Engineering in the Management of Severe Hypospadias. *Front Pediatr* **5**, 283 (2017).](http://paperpile.com/b/zlrttx/36FEM)

90. [Chan, Y. Y. *et al.* The current state of tissue engineering in the management of hypospadias. *Nat. Rev. Urol.* **17**, 162–175 (2020).](http://paperpile.com/b/zlrttx/huZEM)

91. [Lee, H.-R. *et al.* Molecular mechanism(s) of endocrine-disrupting chemicals and their potent oestrogenicity in diverse cells and tissues that express oestrogen receptors. *J. Cell. Mol. Med.* **17**, 1–11 (2013).](http://paperpile.com/b/zlrttx/z369m)

92. [Ogo, F. M. *et al.* Bisphenol A Exposure Impairs Epididymal Development during the Peripubertal Period of Rats: Inflammatory Profile and Tissue Changes. *Basic Clin. Pharmacol. Toxicol.* **122**, 262–270 (2018).](http://paperpile.com/b/zlrttx/nkR2u)
