# Supplementary material for: Spatiotemporal cellular map of the developing human reproductive tract
Source: Nature. 2025 Dec 17;650(8101):428–37. doi: 10.1038/s41586-025-09875-2 (PMC12893920; doi:10.1038/s41586-025-09875-2)
Supplement: Supplementary file 2 — Reporting Summary [file 41586_2025_9875_MOESM2_ESM.pdf]

nature portfolio

Corresponding author(s): Luz Garcia Alonso, John C Marioni, Roser Vento Tormo

Last updated by author(s): Sep 3, 2025

Reporting Summary

Nature Portfolio wishes to improve the reproducibility of the work that we publish. This form provides structure for consistency and transparency in reporting. For further information on Nature Portfolio policies, see our Editorial Policies and the Editorial Policy Checklist.

Statistics

For all statistical analyses, confirm that the following items are present in the figure legend, table legend, main text, or Methods section.

n/a	Confirmed	
☐	☒	The exact sample size (*n*) for each experimental group/condition, given as a discrete number and unit of measurement
☐	☒	A statement on whether measurements were taken from distinct samples or whether the same sample was measured repeatedly
☐	☒	The statistical test(s) used AND whether they are one- or two-sided<br>*Only common tests should be described solely by name; describe more complex techniques in the Methods section.*
☐	☒	A description of all covariates tested
☐	☒	A description of any assumptions or corrections, such as tests of normality and adjustment for multiple comparisons
☐	☒	A full description of the statistical parameters including central tendency (e.g. means) or other basic estimates (e.g. regression coefficient) AND variation (e.g. standard deviation) or associated estimates of uncertainty (e.g. confidence intervals)
☐	☒	For null hypothesis testing, the test statistic (e.g. *F*, *t*, *r*) with confidence intervals, effect sizes, degrees of freedom and *P* value noted<br>*Give P values as exact values whenever suitable.*
☒	☐	For Bayesian analysis, information on the choice of priors and Markov chain Monte Carlo settings
☒	☐	For hierarchical and complex designs, identification of the appropriate level for tests and full reporting of outcomes
☒	☐	Estimates of effect sizes (e.g. Cohen's *d*, Pearson's *r*), indicating how they were calculated

Our web collection on statistics for biologists contains articles on many of the points above.

Software and code

Policy information about availability of computer code

Data collection	No software was used to collect publicly available data. The only publicly available dataset used in this study was downloaded manually from https://www.ncbi.nlm.nih.gov/geo/query/acc.cgi?acc=GSE174712. This information is also included in the "Data Availability" statement of the manuscript.
Data analysis	We list the major libraries for data analysis below:<br>- scRNA-seq data mapping and pre-processing: STARsolo with STAR v2.7.9a; soup removal: CellBender v0.1.0; genotype deconvolution: Souporcell v2.5<br>- scATAC-seq data mapping and pre-processing: CellRanger-atac v2.0.0; CellRanger-arc v2.0.0<br>- Python libraries:<br>general: pandas v1.5.3, numpy v.1.23.0, scipy v1.10.0, scikit-learn v1.2.2, seaborn v0.13.2, matplotlib v3.6.3; single-cell processing: scanpy v1.8.2, anndata v0.8.0, doublet removal: scrublet v0.2.3, automatic cell annotation for organoids: celltypist v1.6.2, data integration and embedding: scvi-tools 0.19.0; knn mapping: iss-patcher v0.1.0; differential expression: PyDESEQ2 v0.4.4<br>- R libraries:<br>general: Matrix v1.6.4, matrixStats v1.2.0, dplyr v.1.1.4, tidyr v.1.3.1, reshape2 v1.4.4, BiocNeighbours v1.20.2, BiocParallel v1.36.0, reticulate v1.35.0; visualisation: ggplot2 v3.5.0, ggrastr v1.0.2, gridExtra v2.3, RColorBrewer v1.1.3; single-cell RNA-seq processing: Seurat v4.3.0, SeuratObject v4.1.4, SingleCellExperiment v1.12.0, Batchelor v1.18.1; data integration and embedding: Harmony v1.2.3; marker identification: SoupX v1.6.2; trajectory inference: Slingshot v1.8.0, tradeSeq v1.4.0; differential abundance: MiloR v1.2.0. single-cell ATAC-seq processing: ArchR v1.0.2<br>- spatial data processing: SpaceRanger v2.1.0; cell2location v0.1.3; squidpy: v1.2.0.<br>- image processing: datashader v0.16.1, holoviews 1.18.3, scikit-image v0.22.0, cellpose v2.1.1, Fiji 1.54j<br>The code used to perform the analyses presented in the manuscript can be found at https://github.com/ventolab/Human-ReproductiveTract-

Development-Atlas.

For manuscripts utilizing custom algorithms or software that are central to the research but not yet described in published literature, software must be made available to editors and reviewers. We strongly encourage code deposition in a community repository (e.g. GitHub). See the Nature Portfolio guidelines for submitting code & software for further information.

Data

Policy information about availability of data

All manuscripts must include a data availability statement. This statement should provide the following information, where applicable:

- Accession codes, unique identifiers, or web links for publicly available datasets
- A description of any restrictions on data availability
- For clinical datasets or third party data, please ensure that the statement adheres to our policy

All raw and processed sequencing and imaging data generated in this study have been deposited in public repositories. Sequencing data are available via ArrayExpress under the following accessions: scRNA-seq (E-MTAB-15475), scRNA-seq from organoids (E-MTAB-15457), scATAC-seq (E-MTAB-15479), and 10x Visium spatial transcriptomics (E-MTAB-15471). Imaging data, including ISS, RNAscope, immunofluorescence, and H&E, are available through the BioImage Archive (accession S-BIAD2224). All datasets are publicly accessible. scRNA-seq data used to generate the figures in this manuscript can also be accessed and downloaded via our web portal: www.reproductivecellatlas.org. Publicly available datasets used in this manuscript were downloaded from ref. 56 (GEO: GSE174712). Source data are provided with this paper.

Research involving human participants, their data, or biological material

Policy information about studies with human participants or human data. See also policy information about sex, gender (identity/presentation), and sexual orientation and race, ethnicity and racism.

Reporting on sex and gender	- scRNA-seq: 47 female and 26 male fetal donors were profiled;<br>- scATAC-seq: 18 female and 11 male fetal donors were profiled;<br>- 10x Visium: 12 female and 8 male fetal donors were profiled. 1 female adult donor was profiled.<br>- In Situ Sequencing: 4 female and 3 male fetal donors were profiled. We could not assign the sex of two donors (Carnagie Stages 18 and 19, respectively) because the reproductive tract is still sexually undifferentiated and we do not have access to their sex chromosome information like in sequencing data)
Reporting on race, ethnicity, or other socially relevant groupings	Data on race, ethnicity and socioeconomic status was not collected
Population characteristics	For the samples used to put together the reference single-cell atlas, age and sex were recorded and used as covariates using the sample integrations:<br>1) scRNA-seq: fetal donors ranged in age from 6 to 21 post-conception weeks and the sex breakdown is reported in the box above<br>2)) scATAC-seq: fetal donors ranged in age from 6 to 21 post-conception weeks and the sex breakdown is reported in the box above
Recruitment	Fetal samples were donated voluntarily by women who have had a termination of pregnancy from clinics collaborating with the Human Developmental Biology Resource, UK, and the NIHR Cambridge Biomedical Research Centre.
Ethics oversight	All tissue samples used for this study were obtained with written informed consent from all participants in accordance with the guidelines in The Declaration of Helsinki 2000. The human embryonic and fetal material was provided by the Joint MRC / Wellcome Trust (grant# MR/R006237/1 and MR/X008304/1) Human Developmental Biology Resource (HDBR, http://www.hdbr.org), with appropriate maternal written consent and approval from the Fulham Research Ethics Committee (REC reference 18/LO/0822 and 23-LO/0312) and Newcastle & North Tyneside 1 Research Ethics Committee (REC reference 18/NE/0290). The HDBR is regulated by the UK Human Tissue Authority (HTA; www.hta.gov.uk) and operates in accordance with the relevant HTA Codes of Practice. This research was also supported by the NIHR Cambridge Biomedical Research Centre (NIHR203312). The views expressed are those of the authors and not necessarily those of the NIHR or the Department of Health and Social Care.

Note that full information on the approval of the study protocol must also be provided in the manuscript.

Field-specific reporting

Please select the one below that is the best fit for your research. If you are not sure, read the appropriate sections before making your selection.

☒ Life sciences ☐ Behavioural & social sciences ☐ Ecological, evolutionary & environmental sciences

For a reference copy of the document with all sections, see nature.com/documents/nr-reporting-summary-flat.pdf

Life sciences study design

All studies must disclose on these points even when the disclosure is negative.

Sample size	No sample size calculation was performed, sample size was determined by the time availability to collect samples.<br>- scRNA-seq: 73 fetal donors;<br>- scATAC-seq: 29 fetal donors;<br>- 10x Visium: 20 fetal donors, 1 adult donor;<br>- In Situ Sequencing: 9 fetal donors.<br>The sample size is sufficient as we have representation of all post-conception weeks between 6 and 21 in at least one technology. For the majority of stages, we have used multiple technologies to profile the samples. This is the largest study of its kind in the field of developmental reproductive biology
Data exclusions	No samples were excluded from the study.
Replication	Validation using alternative approaches was performed to confirm the reproducibility of the findings, including RNAscope (n = 9 donors), In Situ Sequencing experiments (n = 9 donors), immunofluorescence (n = 2 organoid lines). All attempts of replication were successful.
Randomization	Randomization was not performed as it is not applicable to this project (not a case-control study)
Blinding	Blinding was not performed as it is not applicable to this project (not a case-control study)

Reporting for specific materials, systems and methods

We require information from authors about some types of materials, experimental systems and methods used in many studies. Here, indicate whether each material, system or method listed is relevant to your study. If you are not sure if a list item applies to your research, read the appropriate section before selecting a response.

Materials & experimental systems

n/a	Involved in the study	
☐	☒	Antibodies
☒	☐	Eukaryotic cell lines
☒	☐	Palaeontology and archaeology
☒	☐	Animals and other organisms
☒	☐	Clinical data
☒	☐	Dual use research of concern
☒	☐	Plants

Methods

n/a	Involved in the study	
☒	☐	ChIP-seq
☒	☐	Flow cytometry
☒	☐	MRI-based neuroimaging

Antibodies

Antibodies used	TRITC-conjugated Phalloidin (Thermo Fisher Scientific, R37112),<br>Alexa 488- conjugated ZO-1 (Invitrogen, 339188)<br>APC-conjugated EpCAM (BD biosciences, 347200)<br><br>Organoids were washed in PBS prior to incubation with primary antibodies. Antibodies were incubated in an antibody dilution buffer (0.25% Triton-X + 1% FBS in PBST) at 4°C overnight. Organoids were stained with TRITC-conjugated Phalloidin (Thermo Fisher Scientific, R37112, dilution according to manufacturer instructions), Alexa 488-conjugated ZO-1 (Invitrogen, 339188; 1:200 dilution), APC-conjugated EpCAM (BD biosciences, 347200; 1:200 dilution).
Validation	ZO-1 Monoclonal Antibody (ZO1-1A12), Alexa Fluor™ 488<br>Manufacturer: ThermoFisher Scientific<br>Species Reactivity: Dog, Human<br>Host: Mouse<br>Class: Monoclonal<br>Clone: ZO1-1A12<br>Immunogen: Human recombinant ZO-1 fusion protein encompassing amino acids 334-634<br>Conjugate: Alexa Fluor 488<br>Validation: This Antibody was validated by ThermoFisher Scientific<br>BD™ APC Mouse Anti-Human EpCAM<br>Manufacturer: BD Biosciences<br>Species: Human<br>Host: Mouse<br>Class: Monoclonal<br>Clone: EBA-1<br>Immunogen: human epithelial adhesion molecule (EpCAM) that has been identified as a ~40-kilodalton<br>Conjugate: APC

Validation: This Antibody was validated by BD Biosciences
ReadyProbes™ Reagent F-Actin Phalloidin ConjugatesManufacturer: BD Biosciences
ActinRed 555 ReadyProbes Reagent is a selective, high-affinity F-actin probe conjugated to the red-orange fluorescent dye tetramethylrhodamine (TRITC). Phalloidin is a bi-cyclic peptide, commonly used in imaging applications to selectively label F-actin.

Plants

Seed stocks

Report on the source of all seed stocks or other plant material used. If applicable, state the seed stock centre and catalogue number. If plant specimens were collected from the field, describe the collection location, date and sampling procedures.

Novel plant genotypes

Describe the methods by which all novel plant genotypes were produced. This includes those generated by transgenic approaches, gene editing, chemical/radiation-based mutagenesis and hybridization. For transgenic lines, describe the transformation method, the number of independent lines analyzed and the generation upon which experiments were performed. For gene-edited lines, describe the editor used, the endogenous sequence targeted for editing, the targeting guide RNA sequence (if applicable) and how the editor was applied.

Authentication

Describe any authentication procedures for each seed stock used or novel genotype generated. Describe any experiments used to assess the effect of a mutation and, where applicable, how potential secondary effects (e.g. second site T-DNA insertions, mosiacism, off-target gene editing) were examined.
