# Supplementary figures and images for: Spatiotemporal cellular map of the developing human reproductive tract
Source: Nature. 2025 Dec 17;650(8101):428–37. doi: 10.1038/s41586-025-09875-2 (PMC12893920; doi:10.1038/s41586-025-09875-2)

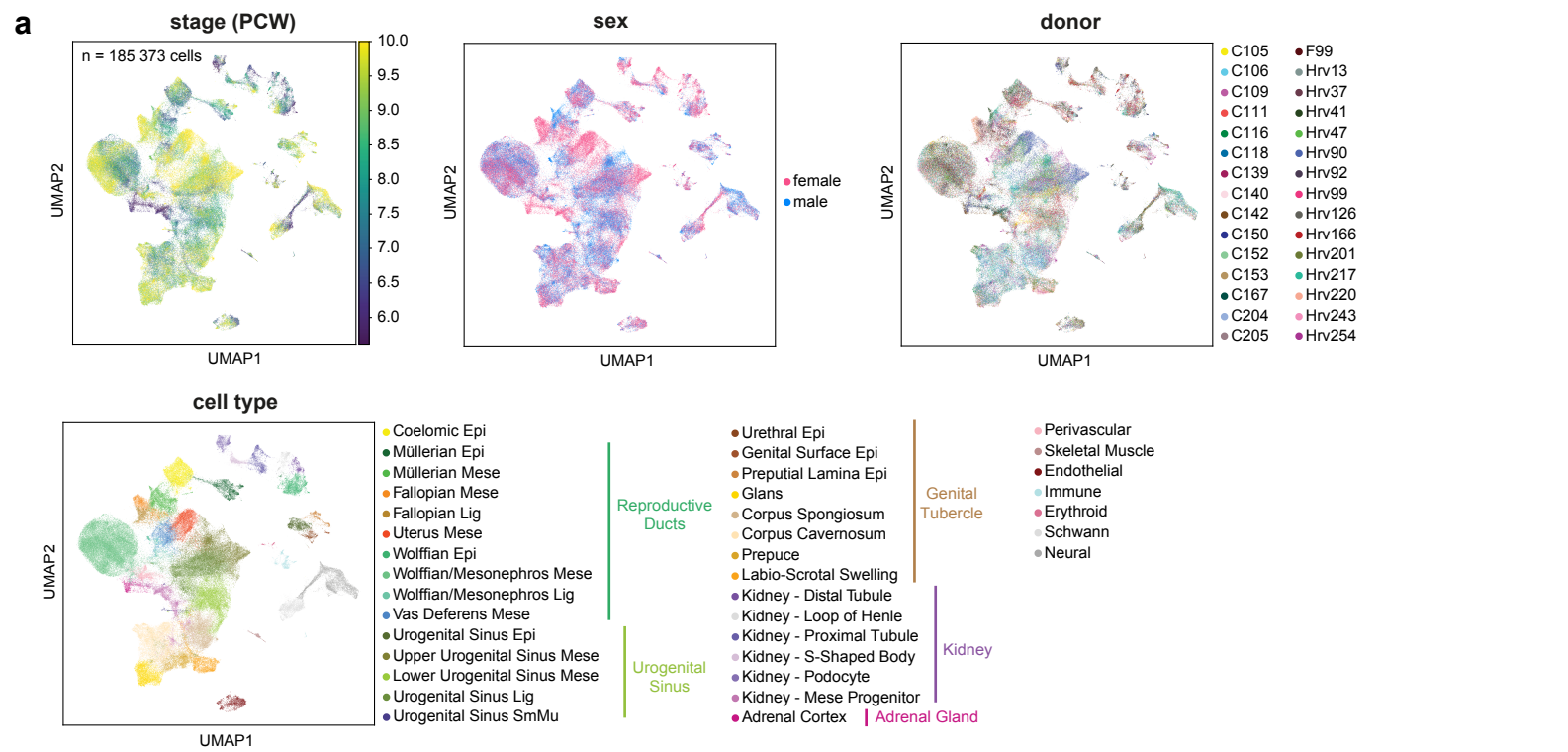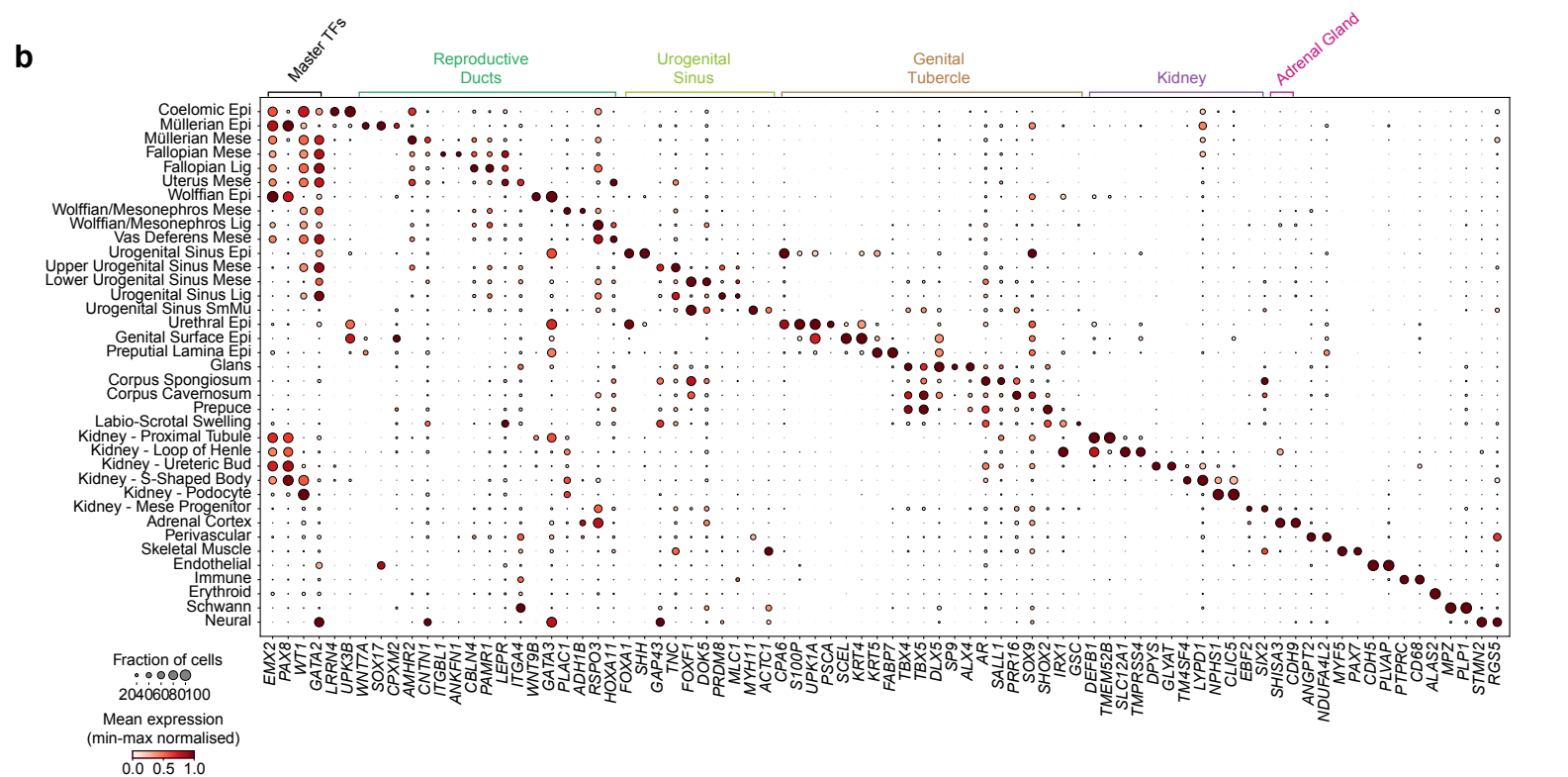

Supplement: Supplementary file 3 — Cell-type characterization of early (≤10 PCW) female and male reproductive tract samples. [file 41586_2025_9875_MOESM3_ESM.pdf]

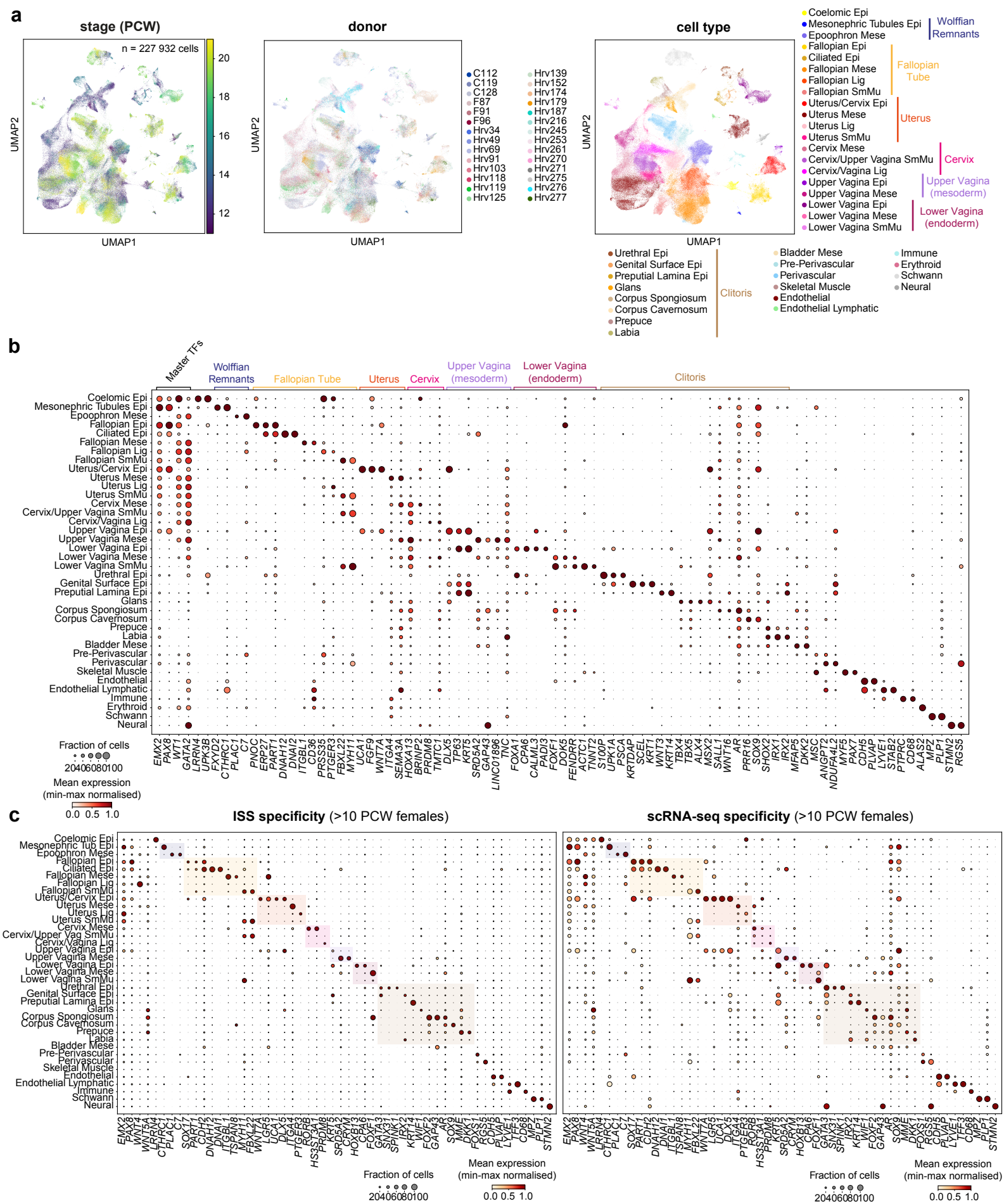

Supplement: Supplementary file 4 — Cell-type characterization of late (>10 PCW) female reproductive tract samples. [file 41586_2025_9875_MOESM4_ESM.pdf]

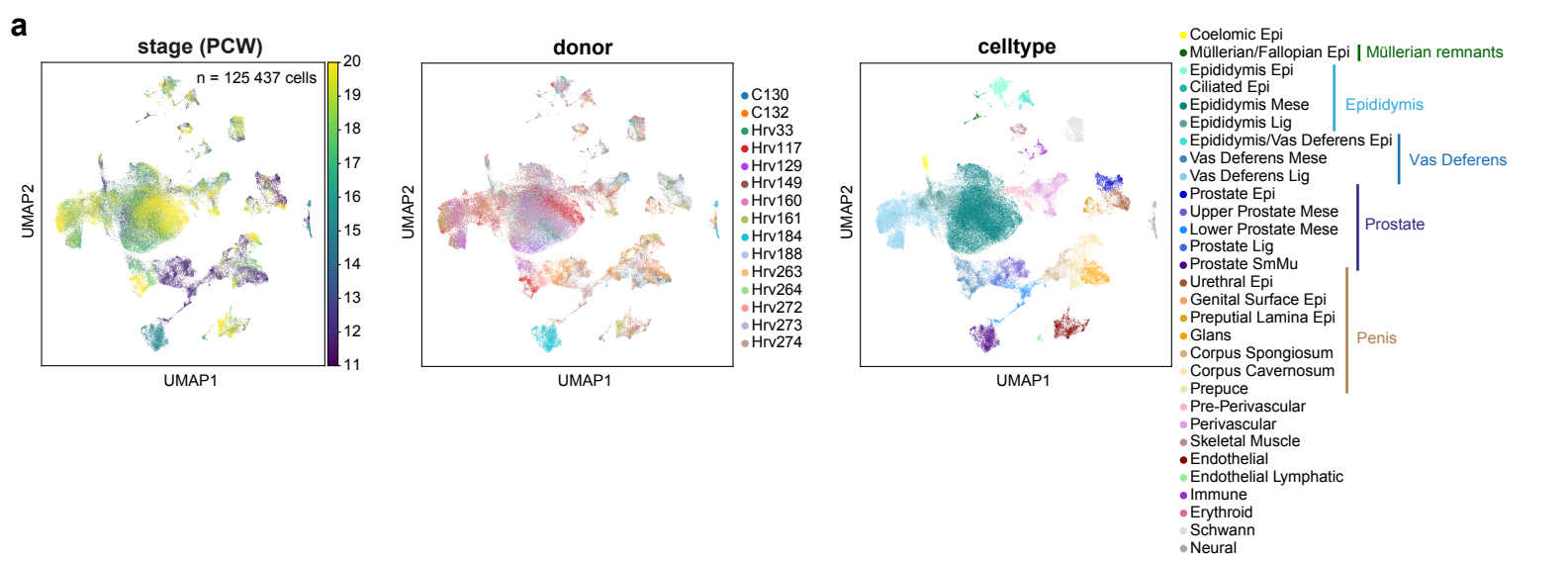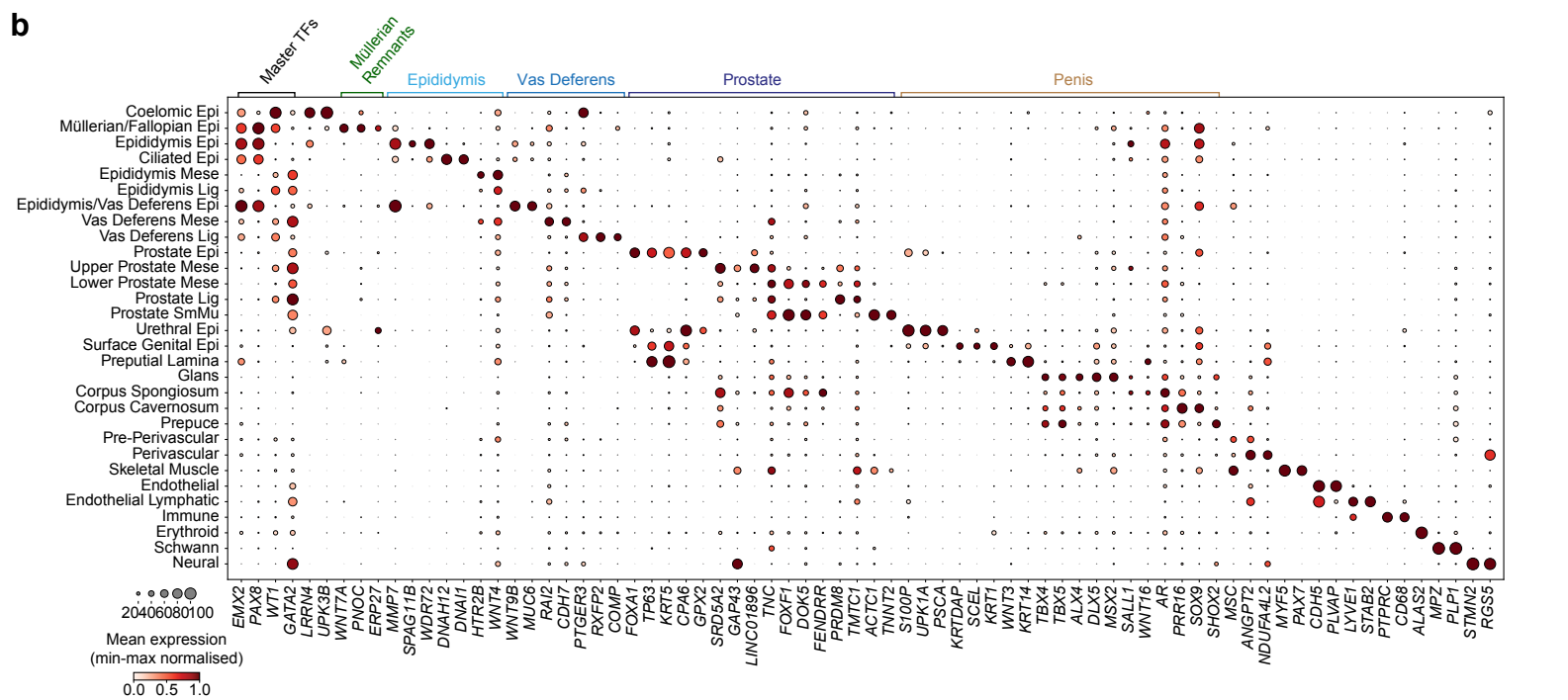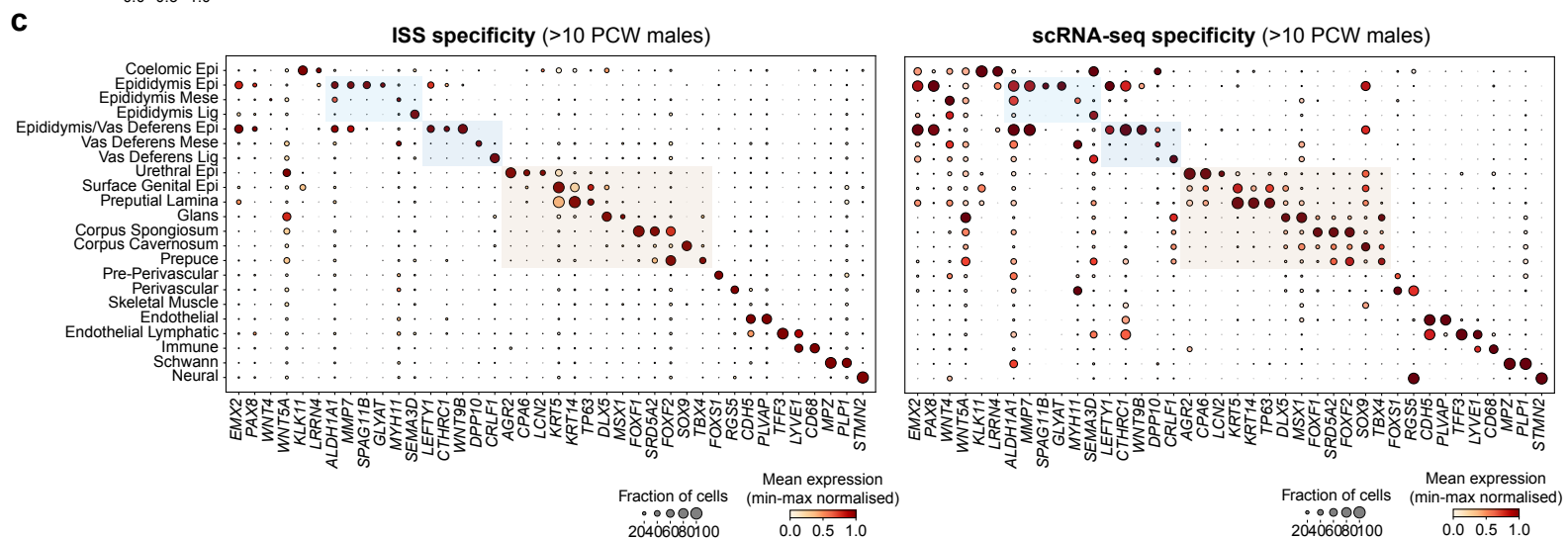

Supplement: Supplementary file 5 — Cell-type characterization of late (>10 PCW) male reproductive tract samples. [file 41586_2025_9875_MOESM5_ESM.pdf]
