# Supplementary material for: Spatiotemporal cellular map of the developing human reproductive tract
Source: Nature. 2025 Dec 17;650(8101):428–37. doi: 10.1038/s41586-025-09875-2 (PMC12893920; doi:10.1038/s41586-025-09875-2)
Supplement: Supplementary file 6 — Supplementary Tables 1–10. [file 41586_2025_9875_MOESM6_ESM.zip › 2024-08-16755D-s6/Supplementary-Tables.docx]

**SI GUIDE - Spatiotemporal cellular map of the developing human reproductive tract**

**Supplementary Table 1**

Sample metadata – see SI for details.

**Supplementary Table 2**

Quality control metrics per sample – see SI for details.

**Supplementary Table 3**

*ISS* gene panel and RNAscope probes – see SI for details.

**Supplementary Table 4**

Marker genes per cell type – see SI for details.

**Supplementary Table 5**

Temporally-variable genes during Müllerian duct formation – see SI for details.

**Supplementary Table 6**

*CellPhoneDB* interactions (Wolffian-to-Müllerian duct signalling during Müllerian formation) – see SIfor details.

**Supplementary Table 7**

Spatially-variable genes along the Müllerian and Wolffian rostro-caudal axes – see SI for details.

**Supplementary Table 8**

Spatially-variable genes along the fallopian tube and epididymal rostro-caudal axes – see SI for details.

**Supplementary Table 9**

*CellPhoneDB* interactions (*early* *corpus spongiosum*-to-urethral epithelium signalling in the external genitalia) – see SI for details.

**Supplementary Table 10**

Clinically approved drugs identified by *drug2cell* as potentially affecting developing reproductive tract epithelia – see SI for details.
